# Gene regulatory network inference in long-lived *C. elegans* reveals modular properties that are predictive of novel aging genes

**DOI:** 10.1016/j.isci.2021.103663

**Published:** 2021-12-20

**Authors:** Manusnan Suriyalaksh, Celia Raimondi, Abraham Mains, Anne Segonds-Pichon, Shahzabe Mukhtar, Sharlene Murdoch, Rebeca Aldunate, Felix Krueger, Roger Guimerà, Simon Andrews, Marta Sales-Pardo, Olivia Casanueva

**Affiliations:** 1 Babraham Institute, Babraham, Cambridge CB22 3AT, UK; 2 Escuela de Biotecnología, Facultad de Ciencias, Universidad Santo Tomas, Santiago, Chile; 3 ICREA, Barcelona 08010, Catalonia, Spain; 4 Department of Chemical Engineering, Universitat Rovira i Virgili, Tarragona 43007, Catalonia, Spain

**Keywords:** Genetics, Genomics, Bioinformatics

## Abstract

We design a “wisdom-of-the-crowds” GRN inference pipeline and couple it to complex network analysis to understand the organizational principles governing gene regulation in long-lived *glp-1*/Notch *Caenorhabditis elegans*. The GRN has three layers (input, core, and output) and is topologically equivalent to bow-tie/hourglass structures prevalent among metabolic networks. To assess the functional importance of structural layers, we screened 80% of regulators and discovered 50 new aging genes, 86% with human orthologues. Genes essential for longevity—including ones involved in insulin-like signaling (ILS)—are at the core, indicating that GRN's structure is predictive of functionality. We used *in vivo* reporters and a novel functional network covering 5,497 genetic interactions to make mechanistic predictions. We used genetic epistasis to test some of these predictions, uncovering a novel transcriptional regulator, *sup-37*, that works alongside DAF-16/FOXO. We present a framework with predictive power that can accelerate discovery in *C. elegans* and potentially humans.

## Highlights

• Gene-regulatory inference provides global network of long-lived animals
• The large-scale topology of the network has an hourglass structure
• Membership to the core of the hourglass is a good predictor of functionality
• Discovered 50 novel aging genes, including *sup-37*, a DAF-16 dependent gene

## Graphical abstract

**Figure:**
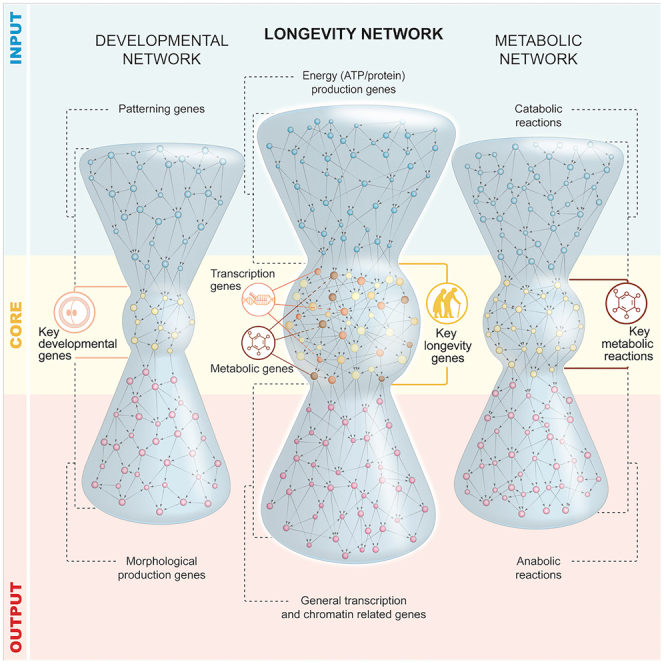

## Introduction

Reductionist single-gene perturbation approaches using *Caenorhabditis elegan*s as a model organism have led to fundamental discoveries in aging (Kappeler et al., 2008; Kenyon, 2011). Two important pathways include the highly conserved insulin-like signaling (ILS) pathway (Kappeler et al., 2008; Kenyon, 2011) and the signals from the germline shown to regulate lifespan in worms, flies, and mammals (Hsin and Kenyon, 1999; Flatt et al., 2008; Benedusi et al., 2015). Although both pathways converge on the key transcription factor DAF-16/FOXO, hundreds of other genes contribute to longevity (Kenyon, 2010). The complexity of the aging process calls for the use of *in silico* systems approaches that can provide a framework to understand the organizational principles governing gene regulatory interactions in aging animals and that can be used to predict aging genes.

Several studies have constructed gene regulatory networks (GRN) from genetic epistasis experiments (Gunsalus and Rhrissorrakrai, 2011; Costanzo et al., 2016; Kuzmin et al., 2018). The most comprehensive study in *C. elegans* queried 65,000 functional interactions and identified 350 genetic interactions (Lehner et al., 2006). Although aging could be studied using an epistasis-based strategy, the time required to probe genome-wide interactions using lifespan assays severely limits the coverage of the network. In fact, a fundamental hurdle in the field is that lifespan assays are time-consuming, even in short-lived *C. elegans* (Soltow et al., 2010; De Magalhães, 2014). Luckily, the high coverage of transcriptomics data opens the door to genome-wide network construction and offers the possibility of studying aging from a systems point of view. During the last decade, GRN inference (NI) methods have used time-series transcriptomics data to infer functional relationships between genes (Greenfield et al., 2013; Siahpirani and Roy, 2016) and have led to the successful identification of lifespan-modulating genes in dietary restricted *C. elegans* (Hou et al., 2016). However, a genome-wide, comprehensive GRN is still lacking in the aging field.

As a consequence, there is no clear systems view of the organization of the regulatory processes affecting aging nor of those that can lead to a longer life. GRNs are often viewed as hierarchical structures with a pyramidal shape in which a few top regulators control downstream genes in a linear fashion with a few interaction loops (Yu and Gerstein, 2006; Erwin and Davidson, 2009). However, the complexity of the aging process—and of the longevity pathways that counteracts it—suggests that a hierarchical, linear structure may not be a suitable description. Previous work has considered biological networks in general as well as GRNs as an information processing system (Csete and Doyle, 2002; Friedlander et al., 2015). Under this framework the flow of information in biological networks resembles an hourglass or bow-tie structure, where complex input signals are integrated by a small core layer and de-coded information is further relayed into a large output layer (Csete and Doyle, 2002; Friedlander et al., 2015).

Several developmental GRNs (i.e., *drosophila* embryonic epidermal patterning) have hourglass structures in which the core “selector” genes integrate information from patterning genes in input layers and relay information into an output layer (Csete and Doyle, 2002; Stern and Orgogozo, 2009; Friedlander et al., 2015). In other bow-tie structures such as metabolic networks, *input* substrates are converted into *output* products via a *core* made up primarily of carriers and precursors (Ma and Zeng, 2003; Csete and Doyle, 2004). The layered topology of these networks has been shown to have both functional and evolutionary implications. In developmental GRNs, modulated expression of the core genes has the largest phenotypic impact and is composed of genes that are hotspots for the evolution of novel phenotypes (Mann and Carroll, 2002; Stern and Orgogozo, 2009). In metabolic networks, the existence of a *core* results in a reduction of enzyme requirements (decreasing genome size) to convert substrates into products (Csete and Doyle, 2004; Tanaka et al., 2005; Zhao et al., 2006).

In this study, we advance the state-of-the-art by developing a “wisdom-of-the-crowds” GRN inference approach coupled to complex network analysis tools to understand the organizational principles governing gene regulation in long-lived animals and to unveil new insights into key longevity pathways. We used temporally resolved transcriptomics data obtained from Notch receptor *glp-1(e2144)ts* mutants, together with a manually curated contextual database, to infer the first genome-wide GRN of the germline longevity pathway *C. elegans*. We used a stochastic block model Bayesian inference approach to find the large-scale organization of the GRN and discovered a layered structure that is topologically equivalent to bow-tie/hourglass networks, referred to as *input-core*-*output* network. To test the functional significance in the context of aging, we performed a genetic screen on 80% of the regulators distributed throughout the network layers and identified 50 novel lifespan regulators, 86% of which have human orthologues and 36% of which are associated with human diseases. The majority of the genes and known key aging modulators such as DAF-16/FOXO and the insulin receptor DAF-2, are enriched in *core* modules, confirming network topology as a good predictor of functionality.

Gene ontology analysis reveals that biological function is heterogeneous across network layers. The input is enriched in genes that control the energetic status (such as ATP and protein production) and this information is relayed to intermediary genes at the core that control downstream transcriptional programs. This flow of information is in line with the known regulatory roles played by ILS which responds to energetic/nutritional stressors by activating homeostatic programs (Murphy et al., 2003; Kenyon, 2010). We generated a data-rich map using both *in vivo* reporters of metabolic targets and gene expression assays which reveal an intricate relationship among fat accumulation, SOD enzymes, and lifespan, as well as a set of novel regulators sharing an intermediary pathway with DAF-16/FOXO and ILS. The systems approach finally led to the identification of pathway dependency of a novel lifespan regulator that modulates lifespan through DAF-16/FOXO, confirmed by epistasis analysis. We have thus applied a powerful approach to studying aging from a systems point of view, which has unveiled that the organization of regulatory interactions has a core of interconnected genes that modulates aging processes. Our work opens the window to a new generation of studies to unravel the systemic complexity of aging processes in *C. elegans* and pave the road for similar approaches in humans.

## Results

### Network inference of genome-wide gene regulation for long-lived *C. elegans*

Our aim was to build a network of gene interactions from transcriptomics data of the long-lived *glp-1(e2144)ts* mutant (referred to as *glp-1(ts)* hereafter) (STAR Methods). We selected *glp-1(ts)* mutants for two reasons. First, animals are devoided of F1 embryos providing reliable transcriptional profiles from aging somatic tissues without the contamination of younger tissues from F1 embryos. Second, the biology of the germline longevity pathway is under-explored compared to other longevity pathways (Lemieux and Ashrafi, 2016).

**Figure 1:**
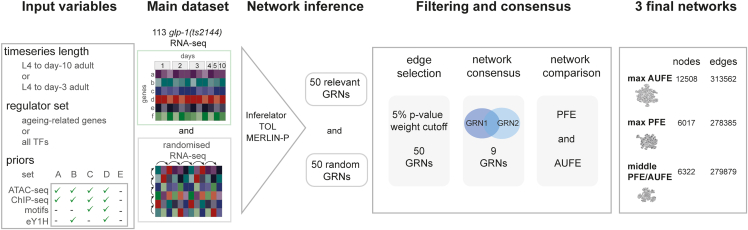
Wisdom-of-the-crowds gene regulatory network inference pipeline. Main datasets 113 time series transcriptomics datasets obtained from *glp-1(e2144)ts* long-lived sterile mutants (Figure S1A). We consider two time series lengths: L4 until day 10 time series and a shorter time series from L4 until day 3—the time frame of maximal gene-expression variability, known to improve results of network inferences (Muldoon et al., 2019). We generated randomized time series data by shuffling both the time and gene dimensions. **Input variables.** We considered three different input variables to NI tools: time series length, regulator set and five different sets of priors as shown (Tables S2 and S17; STAR Methods). Network inference. For each input combination, we ran the three network inference tools: Inferelator, MERLIN + P, and Time-lagged Ordered Lasso (TOL) and obtained 50 gene regulatory networks (GRN). For each GRN, we also obtained the distribution of edge scores from a network inferred from the randomized data. **Filtering and consensus.** We filtered edges using the distribution of edge scores built from randomized data, and set a cutoff at 5% significance. We then grouped networks based on their edge similarity, and obtained a consensus network per group (9 in total) (Tables S4 andS5). We evaluated all 9 networks and all possible combinations of them using functional data as gold standard (Table S3); and precision fold enrichment (PFE) and area under fold enrichment curve (AUFE) as metrics (Figure S1E; STAR Methods). **Final networks.** We selected three final GRNs which scored relatively high for both PFE and AUFE. The figure shows the numbers of nodes and edges for each of the selected GRNs (Table S6).

Adopting a network inference (NI) approach, we inferred genome-wide GRNs from a high-density transcriptomics time series of 12,884 genes of *glp-1(ts)* animals from larval stage 4 (L4) until day 10 of adulthood (a total of 113 libraries) (see STAR Methods and Figure S1A). To obtain reliable GRNs, we devised an NI pipeline based on a “wisdom-of-the-crowds” approach (Marbach et al., 2012; Hill et al., 2016) which considers the GRNs obtained using multiple NI tools; and in addition, expanded the approach by also considering several combinations of input information and introducing a statistical filtering approach to extract signals from noisy transcriptomics data. In what follows, we detail the NI pipeline illustrated in Figure 1 (STAR Methods): from the selection of algorithms and input data, to the filtering, consensus-building and evaluation steps used to build GRNs.

Benchmark studies suggest that inferred GRNs improve by combining results from several algorithms (Marbach et al., 2012; Hill et al., 2016) and by adding existing knowledge of regulatory interactions as a *prior* in the inference process (Marbach et al., 2012; Siahpirani and Roy, 2016). Therefore, we considered three NI tools that can incorporate priors in the inference process (STAR Methods): Inferelator (Arrieta-Ortiz et al., 2015), MERLIN-P (Siahpirani and Roy, 2016) and Time-lagged Ordered Lasso (Nguyen and Braun, 2018) (TOL). Unlike yeast (Costanzo et al., 2016; Kuzmin et al., 2018), *C. elegans* lacks a comprehensive contextual database of regulatory interactions for adult animals to be used as priors. We, therefore, manually curated 380,023 interactions from 289 young adult wild-type (WT) *C. elegans* datasets (Tables 1 and S1; STAR Methods). As prior information we used physical data inferred from techniques providing direct binding information of regulators to DNA (i.e. ChIP-Seq, eY1H and motif analyses; Table S2) and filtered them with adult-specific, ATAC-seq open regions to ensure high accuracy (Figure S1B; STAR Methods; Miraldi et al., 2019, Pique-Regi et al., 2011). As a gold standard (WT-GS) we used functional data obtained from loss or gain of function interventions (Table S3).

**Table 1:** Summary of mechanistically inferred TF-gene, and gene-gene interaction database

Data source	Data type	Number of unique TFs	Number of TF-gene interactions	Number of TF-gene interactions after ATAC-seq filters	Number of TF-gene interactions applicable to our dataset	Number of final unique TF/gene requlators
modERN CIS-BP Fuxman Bass,2016	ChiP-seq	118	185,977	101,597	49,340	57
	Motif	202	479,330	132,732	78,260	273
	eY1H	366	21,714	18,214	13,501	334
		**Total unique applicable TF**				495/892^a^ = 55.49%
Knockout experi-ments	RNA-seq/Microarray		127,480		97,900	126
		**Total unique requlators (TFs and genes)**				621

a According to Kudron et al. (2018), *C. elegans* has 958 predicted TF genes. However, many of these have been classified as RNA-binding or chromatin-remodeling factors, leaving 892 sequence-specific TFs.

Network inference tools take three inputs: (1) Regulators: a set of genes whose expression can affect the expression of other genes; (2) gene expression time series including potential target genes whose expression levels can be affected by regulator genes; and (3) *priors*: known regulatory interactions between regulators and targets. The inference process returns a network of directed regulatory interactions (edges) between regulators and targets. Note that some input regulators may appear only as targets in the final GRN.

To assess the effect of the input information on output GRNs, we considered different combinations of input regulators (2,795 genes, which are either in GenAge database (Tacutu et al., 2018) (22.6%), known transcription factors in *C. elegans* (25.8%), or display high-variability in their expression (51.6%); STAR Methods), time series length, and sets of priors; and inferred a total of 50 GRNs with binary, directed edges from regulators to targets (Table S4). Despite the fact that NI approaches aim to minimize the number of regulatory interactions (for instance, by explicitly incorporating regularization terms in the regression), the inferred GRNs are very dense. On average each regulator has more than 653.6 targets, almost two-fold the maximum number of regulators per target reported in ModERN’s ChIP-seq datasets of 350 (Kudron et al., 2018), suggesting that the number of interactions is overestimated. Each of the NI methods produces different scores for each predicted interaction and there is no standard objective criterion to filter edges that is not based on *ad hoc* knowledge (Arrieta-Ortiz et al., 2015). Therefore, to identify spurious interactions, we compared the edge scores of each network to the distribution of edge scores obtained from a randomized time-series for each input combination and retained interactions that fall below the 5% significance level, reducing to almost a third of the number of targets per regulator (224 on average).

We analyzed the accuracy of inferred networks against WT-GS and found that none of the GRNs performs systematically better than the others (Figures S1C and S1D). Therefore, as a final step in our pipeline, we considered all 50 GRNs to build consensus networks. First, we identified nine groups of networks based on edge overlap (Figure S1E; STAR Methods). Then, for each group we built a consensus network by keeping all unique edges and evaluated the nine consensus networks and all possible combinations of them (Table S5). We finally selected three networks that had relatively high scores when tested against WT-GS (Figure S1F, Table S6; STAR Methods). The selected networks contain only 49.6% of the original input regulators. Encouragingly, the set of regulators in the selected networks have a significantly larger fraction of known aging genes (Tacutu et al., 2018) (33.3%; enrichment of 10% with respect to the input set; pvalue<0.0001), and of human orthologues (60.9%; enrichment of 16% with respect to the input set; pvalue<0.0001).

In the GRN inference field, “wisdom-of-the-crowds” refers to the combination of outputs from multiple NI algorithms. Our pipeline embodies a broader combinatorial approach that considers not only different NI methods, but also different sets of priors, regulators and length of time series input, which are further combined into consensus networks. Our results show that this broad “wisdom-of-the-crowds” combinatorial approach improves the accuracy of inferred GRNs in metazoans (Figures S1D–S1F).

### *In vivo* and *in silico* validations of the GRNs

We used *in silico* and *in vivo* approaches to assess the accuracy of the selected networks. To measure local accuracy, we tested *in vivo* whether the expression levels of predicted target genes are affected when the predicted regulator is perturbed. Using RNA interference, we knocked down ten random regulators (common to the three networks) and quantified expression levels of the predicted targets (36.6 targets per gene on average) by high throughput RT-qPCR. To better assess regulator-target relationships, we exploited the inherent technical variability of the RNAi technique (Kamath and Ahringer, 2003) (Figures S2A and S2B) and calculated the Pearson correlation coefficient (PCC) between the changes in expression levels of regulator-target pairs from at least six replicates (Figure S2B, Table S7, STAR Methods, https://s-andrews.github.io/wormgrn/qpcr/)

**Figure 2:**
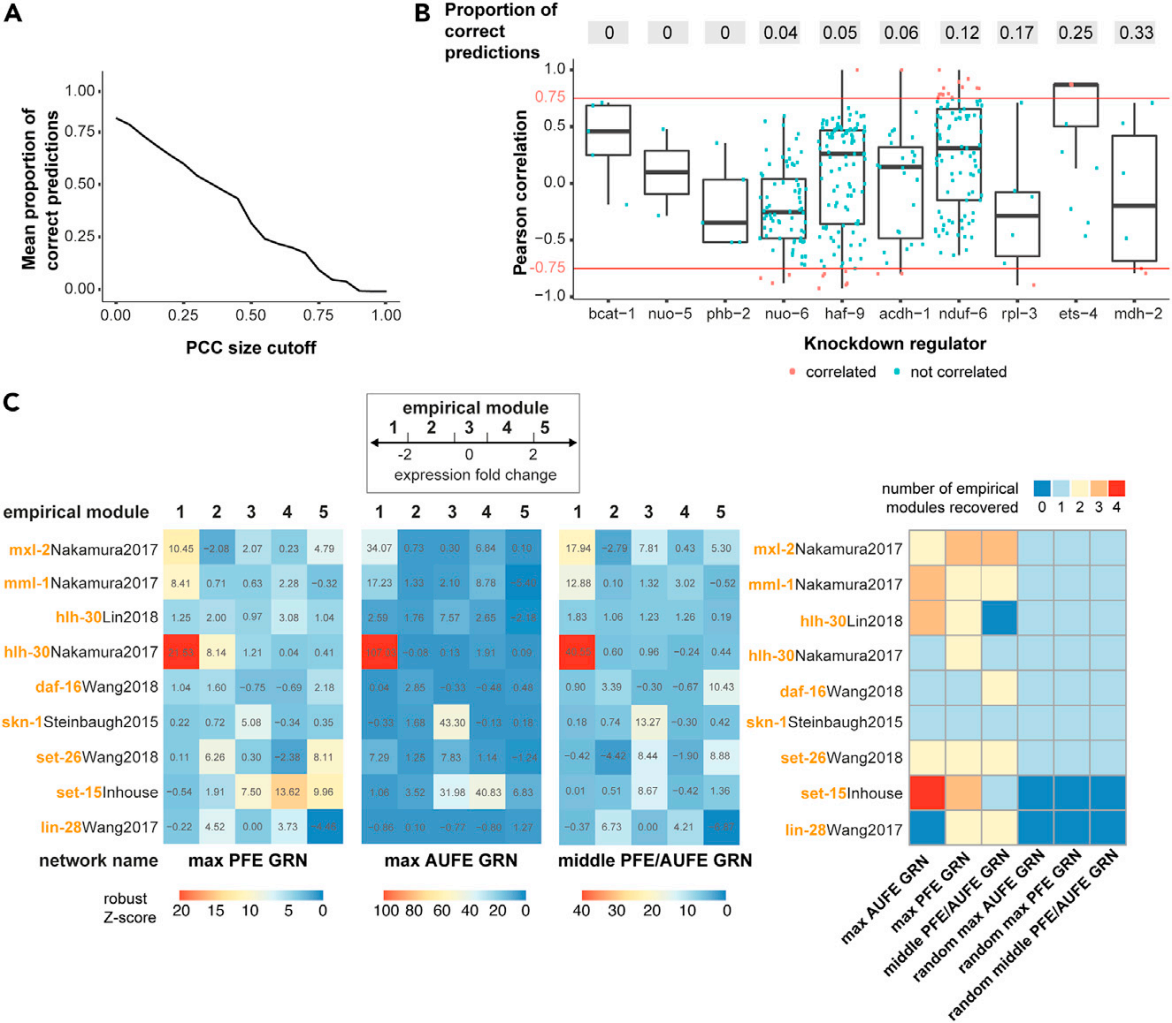
*In vivo* and *in silico* validation of the inferred gene regulatory networks (A and B)*In vivo* validation. We knocked down ten regulators selected at random using RNAi expressing bacteria from larval stage 4 (STAR Methods). We calculated PCC between the relative change in expression levels of regulator and target pairs (ddCt) obtained from at least six independent biological replicates. We considered an interaction correctly predicted *in vivo* when the size of its PCC is higher than corresponding PCC cutoff values. (A) Mean proportion of correctly predicted interactions of the final 3 networks as a function of Pearson's correlation (PCC) size cutoff. (B) Correctly predicted *in vivo* interactions for each regulator. Each dot represents the PCC of the ddCt values between one target and one knockdown regulator (Xaxis). The red lines indicate the PCC cut-off (PCC≥ |0.75|). At the top, we show the proportion of correctly predicted interactions per regulator (Table S7). (C) *In silico* validation of network topology. We compared structural modules in the GRNs to five empirical gene modules, obtained from RNA-seq datasets specific to *glp-1(e2144)ts* (Table S9; **main text**, STAR Methods). For each one of the GRNs, we display the maximum robust Z-scores of Jaccard similarity index between each empirical module and a structural module. A significant robust *Z* score (robust *Z* score> 1.96, pvalue of 0.05 based on a right-tailed Fisher's exact test) indicates that an empirical module was recovered by a structural module. The right most plot summarizes the number of empirical gene modules recovered by each inferred network and modules recovered by randomizing gene membership to structural modules with the same number of genes.

To determine whether the knockdown (KD) of a regulator affects a target, we chose the PCC cut-off value at the inflection point of a curve obtained when plotting the average precision versus cut-off values (PCC = 0.75, Figure 2A). Overall, 61.71% of the interactions in the inferred GRNs (including positive and negative interactions) are recovered *in vivo*; however, the overall precision for positive interactions is equal to 11.74% (mean precision of positive interactions for KD regulators equal to 10.21%; AUPR = 0.193) (Figure 2B, Table S7). These results suggest that inferred GRNs have a high number of false positive interactions, but the number of false negative interactions is very low. Indeed, the observed accuracy at recovering interactions is significantly larger than the random expectation obtained by rewiring the positive interactions in the inferred networks (*Z* score = 7.95), but the precision is not (*Z* score = −1.70). Nonetheless, the precision is higher than the expected precision of a random network between the initial set of regulators and targets, which is equal to 0.9% (STAR Methods, for similar analyses see Marbach et al., 2012; Siahpirani and Roy, 2016; Miraldi et al., 2019). We also find that as we increase the number of tested targets per regulator, the number of correctly predicted edges for each regulator fluctuates less and approaches the mean precision (Figure S2C). The apparent convergence toward the mean suggests that despite this being a very partial validation (we only tested targets for 10 out of 1396 regulators), the errors we observe are unbiased (Figure S2C).

From these observations, we hypothesize that if errors affect all nodes in the same way, the global structure of GRNs should still capture the biologically relevant organization. To test this hypothesis, we described the global structure of GRNs by grouping genes into structural modules and then assessing whether structural modules in the GRNs are significantly correlated with empirical modules obtained from orthogonal RNA-Seq data. To obtain structural modules, we used stochastic block models (SBM), which assume there exist underlying groups of genes with the same connectivity patterns that give rise to the observed network. Following a Bayesian approach, we selected the SBM that best describes each GRN to define its structural modules (Peixoto, 2014; Vallès-Català et al., 2018) (Tables S8A–S8C; STAR Methods). To obtain empirical modules, we curated published transcriptomics datasets of young adult *glp-1(ts)* animals in response to loss of function of regulators (GLP-GS, Table S9). Because we inferred our networks from the observed changes in gene expression, we considered as empirical modules the groups of genes that respond to a genetic modification in a similar manner, and divided genes into five empirical modules according to the levels of observed gene expression changes (Figure 2C; STAR Methods).

We use two different approaches: a topological approach, using the Jaccard similarity index, and an information-theoretic approach, using the adjusted mutual information. Using the Jaccard similarity index we find that structural modules recover at least three empirical modules in 5 of the 9 GLP-GS datasets, and on average 2.8 modules across the three selected networks–more than the (at most) one module we expect to recover if we distribute genes in groups at random (Figure 2C; STAR Methods). Using the adjusted mutual information, we find statistically significant correlations between structural modules and empirical modules (Figure S2D; STAR Methods). The significant similarities we observe indicate that the global structure of the inferred GRNs is biologically meaningful.

### Topological analysis of the aging network defines input, core and output layers

**Figure 3:**
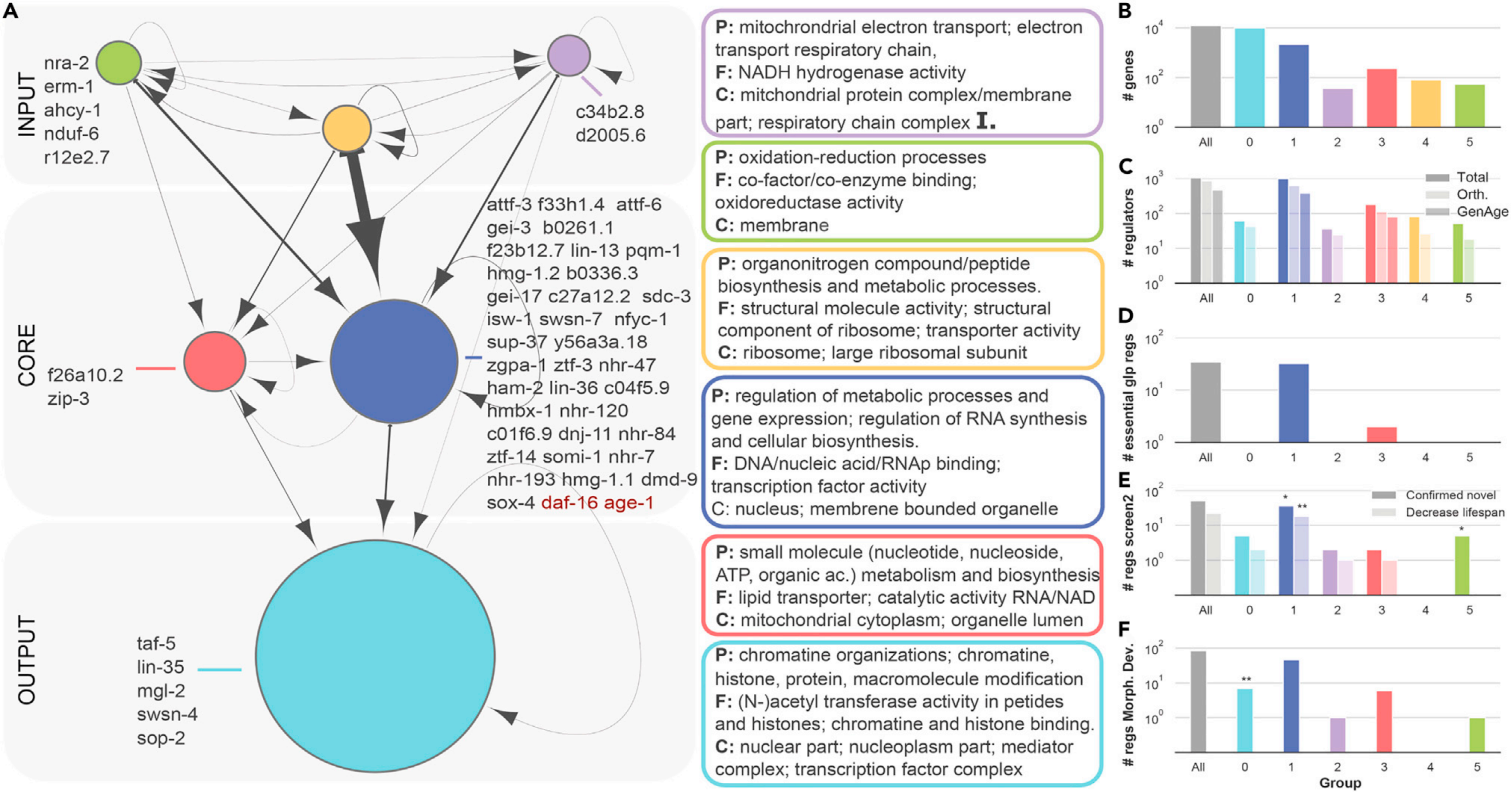
The global organization of the gene-regulation network reveals an input-core-output structure with aging modulators at its core (A) Global organization of the network and GO enrichment for each structural module. Each node represents a module. We obtained modules using a Bayesian model selection approach with hierarchical stochastic block models (Table S8; STAR Methods). The area of the nodes is proportional to the number of genes it comprises (shown in b); edge thickness is proportional to its weight (we only represent edges with weight >260-- see STAR Methods for a table of edges). The network has three main layers: input, core and output. We list novel aging regulators in each module; we include *daf-16* and a*ge-1* (red) for reference. Boxes show GO enrichment terms of the regulators in each module following the color code of the network nodes (P: Process, (F) Function, (C) Component). (B) Number of genes in each module. Bars follow the same color code of nodes in (A). Gray bars show network totals. (C) Number of regulators, number of regulators with a human orthologue and number of regulators in GenAge. (D) Number of known essential genes in *glp-1 C. elegans* (Table S11) that appear as regulators in the network. (E) Number of tested and confirmed aging genes in the second screen (see text). ∗,∗∗ show enrichment with respect to the random expectation of hits given the number of non-GenAge regulators in each module (∗ pvalue <0.1, ∗∗ pvalue <0.05). (F) Number of regulating genes that cause defects in body morphology (from Kamath and Ahringer, 2003). These results are for the largest consensus GRN (max AUFE) and are consistent with the results of the other networks (Figures S3 and S4).

Our analysis shows that NI tools overestimate the number of interactions in an unbiased way, and that despite the presence of errors at a local level, the large-scale structure of the networks is well-correlated with biological function. We therefore focus on analyses of the structural modules, the regulatory interactions among them as well as their content. In what follows, we discuss results for the largest GRN we inferred (Table S6A), but the same holds true for the remaining GRNs (Figures 3A–3F, S3, and S4, Tables S6B and S6C). We find that the network structure is topologically equivalent (99.8% of the interactions follow this pattern; STAR Methods) to a “bow-tie” or “hourglass” structure with three regulatory layers: *input*, *core*, and *output* (Figures 3A, S3A, andS4A; STAR Methods). As in hourglass/bow-tie structures observed in biology (Friedlander et al., 2015), modules in the *input* layer (top regulators) regulate any other modules but are exempt from regulation from below; modules in the *core* are regulated by modules in the *input* layer and regulate modules in the *output* layer; finally, modules in the *output* layer do not regulate any other modules.

In this *input-core-output* network, we observe that regulators with human orthologues are evenly distributed across modules, suggesting a conserved structure (Figures 3C, S3C, and S4C). In an hourglass model, topology is predictive of functionality. For example, genes essential for epidermal patterning in *drosophila* are concentrated at the “core” typically the smallest of all layers (Csete and Doyle, 2002; Friedlander et al., 2015). In a bow-tie model, the same holds true. In metabolism, the existence of a bow-tie knot of common carriers and precursors that link metabolic substrates to metabolic products allows the robust regulation of metabolic processes (Csete and Doyle, 2004). The *input-core-output* network is more reminiscent of metabolic networks in that the core is thick, containing a larger number of genes than the thin waist of hourglass developmental networks (Figures 3B, S3B, and S4B). The thick core of the *input-core-output* network thus raises a question regarding its functional importance in an aging context.

### Genetic screen identifies novel and conserved aging genes

**Figure 4:**
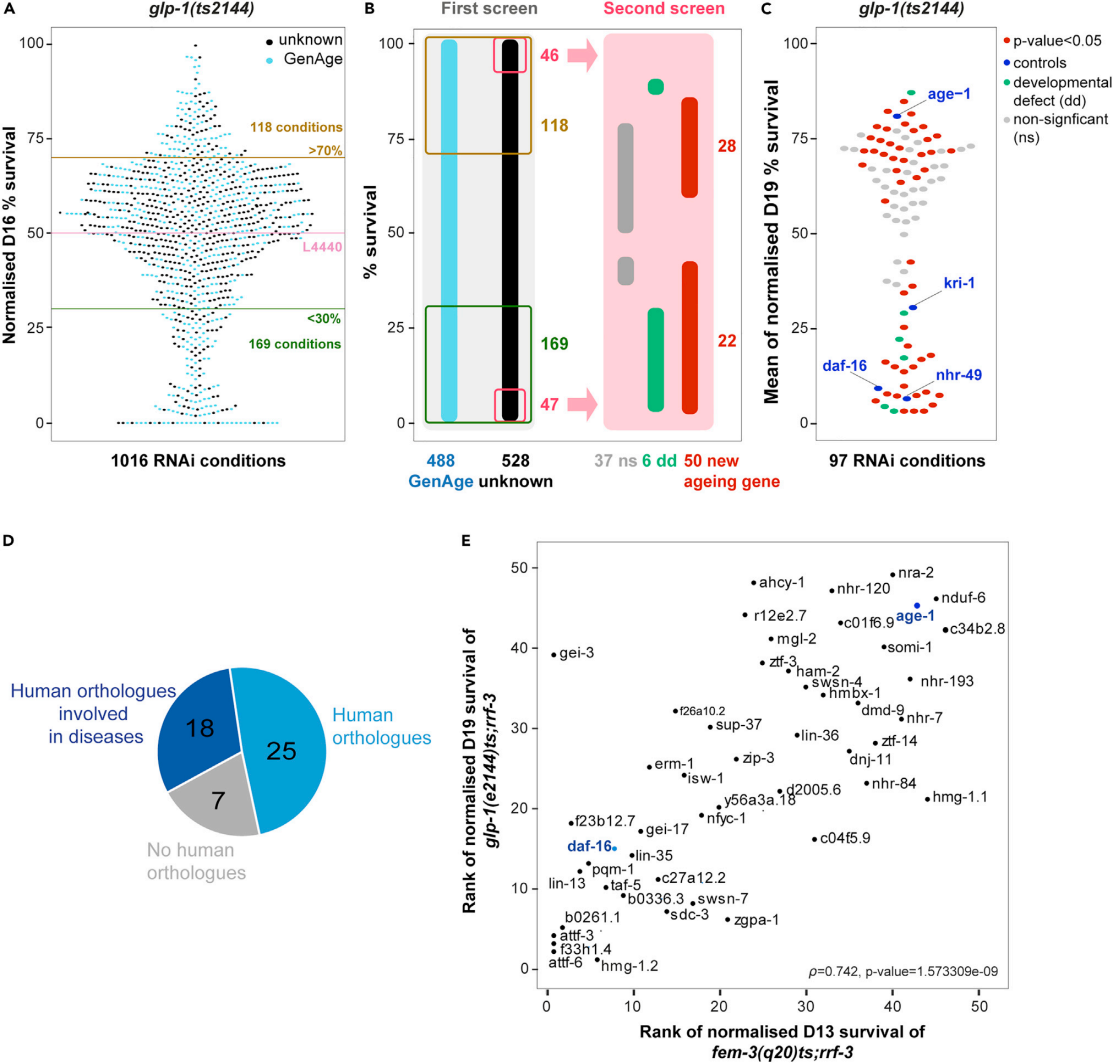
Two-step RNAi screening uncovers 50 novel modulators of the germline longevity pathway, many of them conserved in human and involved in the modulation of wild type lifespan (A) Normalized percentage of survival at day 16 (D16) of adulthood for *glp-1(e2144)ts;rrf-3* worms grown in the presence of RNAi from larval stage 1 (L1). Data has been normalized to the survival of *glp-1(e2144)ts* at D16 of adulthood fed with bacteria expressing an empty vector (L4440) which corresponds to ∼50% survival (Table S10). Blue dots indicate knockdown of genes which have previously been linked to aging according to GenAge. (B) Two-step screening strategy and summary of the results. The left gray box depicts the first screen (A) where 1016 regulators were tested--48% of them are already classified as aging genes (GenAge). Among the genes which showed significant *glp-1(e2144)ts;rrf-3* lifespan changes and were not in GenAge, 93 candidates with largest change were re-screened using high-resolution lifespan assays (pink box). The second screen monitored worms at twelve time points in triplicate (STAR Methods). Numbers indicate the number of genes following the color code of the boxes. (C) Normalized mean survival for *glp-1(e2144)ts;rrf-3* worms treated with RNAi from L1 stage. Mean survival was calculated by averaging the percentage of survival at D19 of adulthood of three biological replicates. Normalization was relative to the mean survival of control animals fed with bacteria expressing L4440 at D19 adulthood. pvalues were calculated using a logrank test for each replicate and combined using Fisher's method. Bonferroni was applied for multiple comparison correction (Table S12). (D) The pie chart represents the proportion of genes in the 50 newly-discovered aging genes with a human orthologue and/or a human disease linkage. (E) Correlation between the rank of normalized mean survival of *glp-1(e2144)ts;rrf-3* worms and *fem-3(q20)* treated with bacteria expressing 50 dsRNAs from L1 stage. (Table S14A). We ranked genes according to the mean percentage of survival of long-lived *glp-1(e2144)ts* at D19, and the mean percentage of survival of normal lived *fem-3(q20)ts* at day 13 (D13). The RNAi conditions correspond to the novel aging genes. We used Spearman's ⍴ to quantify the correlation between the ranked lists of genes (⍴=0.742, pvalue <2 x 10^−9^).

To experimentally test the functional roles of the three structural layers, we performed a two-step blind screen on 80% of the regulators randomly distributed across all modules. First, we measured survival rate at day-16 of adulthood after knocking down gene function on 1,120 regulators (Figure S5A, Table S10; STAR Methods). We validated the screen performance by blindly finding two independent RNAi clones of *daf-16 (daf-16i)* and *age-1 (age-1i),* two well-established regulators of *glp-1(ts)* lifespan (Lin et al., 2001; Berman and Kenyon, 2006) (Figures S5B and S5C). In this screen we find that 26% of the knockdowns (287 genes; 169 shorter, and 118 longer lifespan) altered the mean survival of *glp-1(ts)* mutants by at least 20% and that among those, 62% (179 genes) have already been linked to wild-type aging in GenAge (Figure 4A), a significant enrichment compared to the original set of regulators (pvalue<0.0001, STAR Methods).

Next, we selected 93 candidates that showed the largest lifespan change and had not previously been linked to aging (Figure 4B, Table S11), and assayed the lifespan of them by monitoring the percentage of survival of *glp-1(ts)* worms treated with RNAi from larval stage 1 (L1) at twelve time points in triplicate (Figures 4C andS5C, Table S12). Six genes were excluded due to abnormal morphology; and 50 conditions showed a significant change in lifespan (22 shorter, and 28 longer lifespans; Figures 4B and 4C, **black dots** and Table 2).

**Table 2:** Novel aging genes

Name	Human orthologue	Human disease	Molecular function	% Lifespan change at D19	χ2-adjusted pvalue
Attf-3	High mobility group (HMG)		HMG/Chromatin remodeling	−45.96	1.65E-50
f33h1.4	Uncharacterized			−45.96	9.11 × 10^−48^
Attf-6	High mobility group		HMG/Chromatin remodeling	−45.96	8.59 × 10^−44^
Gei-3	CIC	Yes	Transcriptional repressor	−44.38	2.38 × 10^−32^
b0261.1	BDP1		TF	−43.33	5.64 × 10^−25^
f23b12.7	CEBPZ		DNA binding	−42.07	2.59 × 10^−21^
Lin-13	ZNF423; ZNF462; and ZNF786.	Yes	Zinc finger protein	−41.94	1.10 × 10^−29^
Pqm-1	Sal-like protein 2		TF	−41.9	1.14 × 10^−15^
Hmg-1.2	HMGB1, HMGB3	Yes	HMG	−41.53	1.10 × 10^−19^
Taf-5	TAF-5		TATA box TF	−41.06	1.04 × 10^−28^
Daf-16	FOXO (CONTROL)	Yes	TF	−40.09	4.21 × 10^−35^
b0336.3	RBM26		RNA binding	−39.53	3.92 × 10^−21^
Lin-35	RBL1; RBL2	Yes	Transcriptional corepressor	−35.8	3.89 × 10^−19^
Gei-17	PIAS2; PIAS3, STAT 4		TF	−34.55	7.61 × 10^−16^
Erm-1	EZR, MSN, RDX	Yes	Ezrin-radixin-moesin protein	−34.52	1.01 × 10^−13^
c27a12.2	ZNF791	Yes	Zinc finger protein	−33.3	3.46 × 10^−12^
Sdc-3	CPA3 (A3); CPA4; CPB2	Yes	Carboxypeptidase	−32.63	2.98 × 10^−7^
f26a10.2	ZBTB32; ZFP91-CNTF	Yes	TF	−31.57	4.51 × 10^−14^
Isw-1	SMARCA1 (SWI/SNF related)		Predicted to be TF	−29.18	5.04 × 10^−13^
Swsn-7	ARID2	Yes	Chromatin remodeling	−24.35	3.46 × 10^−7^
Nfyc-1	NFYC		TF	−15.5	1.79 × 10^−4^
Sup-37	Uncharacterized			−13.74	1.32 × 10^−14^
y56a3a.18	ZNF593		Zinc finger protein	−7.531	2.19 × 10^−6^
Zgpa-1	ZGPAT		Zinc finger protein	8.194	1.32 × 10^−2^
Zip-3	ATF5		TF	14.23	6.06 × 10^−4^
r12 × 10^2^.7	Uncharacterized			16.05	4.49 × 10^−2^
Ahcy-1	AHCY	Yes	Adenosyl-hydrolase	16.24	2.69 × 10^−2^
Ztf-3	Zinc finger protein 394		Zinc finger protein	17.51	3.93 × 10^−6^
Mgl-2	GRM1 and GRM5	Yes	Glutamate receptor	19.19	1.48 × 10^−5^
d2005.6			Membrane-associating domain; and Marvel domain	20.18	1.83 × 10^−7^
Ham-2	PRDM16		Zinc finger protein	20.24	4.83 × 10^−3^
Lin-36			DNA and metal ion binding	20.78	2.43 × 10^−3^
Tkt-1	Uncharacterized (CONTROL)		Transketolase	21.01	1.09 × 10^−7^
Swsn-4	SMARCA2	Yes	SWI/SNF-chromatin remodeling	21.05	5.00 × 10^−4^
c04f5.9	Zinc finger protein 319		Zinc finger protein	21.13	2.28 × 10^−3^
Sox-4	SRY	Yes	Sox protein	21.54	4.00 × 10^−2^
Hmbx-1	HMBOX1		Homeobox TF	21.86	6.44 × 10^−4^
Nhr-120	HNF4A; NR2C2 RXRB	Yes	TF	22.97	4.05 × 10^−4^
c01f6.9	ZNF706		Zinc finger protein	23.83	2.79 × 10^−4^
Dnj-11	DNAJC2		Hsp40	24.06	2.15 × 10^−3^
Dmd-9	Isoform 1 of Doublesex- and mab-3-related transcription factor 2		DNA binding TF	24.49	6.50 × 10^−4^
Nhr-47	Isoform HNF4-Alpha-8 of Hepatocyte nuclear factor 4-alpha		TF	24.58	3.83 × 10^−2^
Nhr-84	Nuclear receptor subfamily 2 group E member 1		TF	24.99	3.90 × 10^−3^
Sop-2			Polycomb protein	25.22	1.85 × 10^−2^
Ztf-14	GLIS1; GLIS3; ZXDA	Yes	Zinc finger protein	26.38	1.99 × 10^−9^
Somi-1	Uncharacterized			26.79	5.76 × 10^−8^
Nra-2	Ncln		Nicalin	27.38	2.88 × 10^−3^
Nhr-7	Uncharacterized		TF	27.39	2.93 × 10^−3^
Nhr-193	HNF4-Alpha-2		TF	28.08	2.82 × 10^−6^
Age-1	PIK3CA, PIK3CD (CONTROL)	Yes	Kinase	30.07	1.05 × 10^−13^
Hmg-1.1	High mobilty group		Chromatin remodeling	30.68	1.58 × 10^−7^
Nduf-6	NDUFS6		Ubiquinone oxidoreductase	31.34	7.48 × 10^−8^
c34b2.8	NDUFA13	Yes	Ubiquinone oxidoreductase	33.87	2.02 × 10^−15^

We list the novel aging genes, its human orthologues, human disease linkage, their known function, and mean lifespan change at day 19 of adulthood upon knockout in *glp-1(ts)* animals. pvalues from the logrank survival statistical test on 3 independent biological values were combined using Fisher's method and adjusted using Bonferroni correction (Table S12). TF: transcription factor. Although *pqm-1* has been shown to be involved in the ILS pathway (Tepper et al., 2013), it is missing in GenAge and has not been described in *glp-1(ts)*. *daf-16*, *age-1*, and *tkt-1* are shown as controls. Table S13 lists other controls which also match expected results.

As an initial characterization, we addressed their level of conservation. As shown in Figure 4D and Table 2, 86% of the novel genes have corresponding human orthologues and 36% of them have been linked to a human disease (Table S13). Among the novel aging genes, 58% of them encode for genes with DNA binding roles, including transcription factors (such as Zinc-finger proteins, TATA box and Homeobox containing genes), and 12% of genes contain AT-Hook and SWI-SNF domains, typical of chromatin binding complexes. In addition, we find genes encoding metabolic enzymes (carboxypeptidase, adenosyl-hydrolase, and ubiquinone oxidoreductases); a glutamate receptor, a gene encoding a protein folding chaperone and several novel genes of unknown function (Table 2).

Most of the general biological roles of these genes have been linked to aging in wild-type animals (Tacutu et al., 2018), therefore we hypothesized that their function is not specific to the germline longevity pathway, but that they also play a conserved role during normal aging. To test this hypothesis, we measured the percentage of survival of a normal-lived sterile mutant, the *fem-3(q20)ts* mutation which causes worms to possess a normal somatic gonad that produces no oocytes but excess sperm (Barton et al., 1987). Indeed, we observed that the effect on the survival rate of *fem-3(ts)* and *glp-1(ts)* animals under same RNAi conditions are significantly correlated (Spearman's ⍴ = 0.742, pvalue = 1.6 × 10^−9^), indicating that the novel aging genes have a species-wide rather than mutant-specific role (Figure 4E, Table S14A). To rule out spurious effects caused by the L1 larval RNAi treatment, we investigated whether the identified genes caused similar changes to lifespan if they were knocked down only during adulthood. We find that the identified lifespan-altering genes are genuine. The mean survival rates of *glp-1(ts)* worms treated with RNAi from the L1 larval stage are strongly correlated with those treated from L4 larval stage (Spearman's ⍴ = 0.735, pvalue = 5.5e-10), with only 8% of the tested genes showing small significant differences between treatments (Figure S5D, Table S14B).

### Aging genes are enriched in the core module of the network

The analysis of the distribution of the genes in the *input-core-output* network highlights the importance of the role played by genes at the core for the longevity of *glp-1(ts)* animals. Most of the 50 novel genes concentrate in the largest *core* module (Figures 3E, S3E, and S4E). While this concentration could be explained by the large size of the core (pvalue = 0.094), it is in line with the fact that known regulators in GenAge are exclusively located in core modules (Figures 3C, 3D, S3C, S3D, S4C, and S4D). Furthermore, regulators shown to shorten *glp-1(ts)* lifespan are also primarily located in core modules, including all 80 previously known *glp-1(ts)* aging regulators, the key longevity gene *daf-16* (Figure 3D, Table S11), and 19 of the 22 novel lifespan-shortening genes uncovered in this study, a significant enrichment compared to a uniform distribution of genes across modules (pvalue = 0.021 for modules at the core, 19/22; pvalue = 0.045 for the largest core module, 18/22) (Figures 3A, 3E, S3E, and S4E). Note that we also found that genes causing developmental defects (Kamath and Ahringer, 2003) are not enriched in the input modules (Figures 3F, S3F, S4F), ruling out the bias that the RNAi treatment on L1 larvae can introduce by favoring the enrichment of developmental genes over aging genes.

Gene Ontology analysis reveals that biological functions are heterogeneously distributed across modules (Figures 3A, S3A, and S4A). *Input* modules are enriched in genes that modulate the generation of energy (ATP), proteins (via translation regulation) and other structural components of the cell (Eden et al., 2009). C*ore* modules are enriched in genes that control metabolism and play regulatory functions in the nucleus and the mitochondrion, which have been linked to aging (López-Otín et al., 2016). The *output* module is enriched in genes that control chromatin including histones, histone acetylation, and basal transcriptional regulation (Figures 3A, S3A, and S4A). The organization of the network is consistent with the conserved regulatory role of known longevity pathways, where nutrient/energetic stress is communicated via insulin receptor DAF-2 (in ILS pathway) or via KRI-1 (in the germline longevity pathway) to the transcription factor DAF-16/FOXO, master regulator of stress and metabolic genes (Kenyon, 2010). The fact that most longevity genes are within the thick, tightly connected core module showcases the complexity of the regulatory interactions that govern the germline longevity pathway. One key question is then how the novel aging regulators interact with *daf-16/foxo* and ILS as part of the *core* modules.

### Global characterization of the novel aging genes reveals genes sharing the same metabolic features and pathways as DAF-16/FOXO and ILS

To provide a global characterization of the novel longevity genes regarding key metabolic activities related to FOXA/PHA-4, ILS and DAF-16/FOXO, we generated a data-rich map using *in vivo* transcriptional reporters. First, we quantified in a semi-automatic manner (STAR Methods) the *in vivo* expression of a target of the pha-4/FOXA transcription factor, *lgg-1*. LGG-1 is a protein present at the membrane of phagophore and autophagosome (Wu et al., 2012) and PHA-4/FOXA is a key determinant of *glp-1ts* lifespan (Lapierre et al., 2011). We found that the aging genes: *hmg-1.2i* ang *gei-17i* trigger a significant increase of LGG-1 signal (Figure S6A) suggesting that both *hmg-1.2* and *gei-17* could modulate lifespan via PHA-4. We studied two other *in vivo* reporters and used *daf-16* and *age-1* -a key PI3 kinase downstream of DAF-2- as controls. The first is DHS-3, which localizes to the worm's main fat storage compartment, the intestinal lipid droplets (LD) (Na et al., 2015). We observed that in the *glp-1(ts)* animals LD stores increased under *daf-16i* while remaining unaltered under *age-1i* (Figures S6B and S7, Table S15). In parallel we used a transcriptional reporter to monitor the expression of *superoxide dismutase 3* (*sod-3*), a direct target of DAF-16 that functions to detoxify oxidative stress in mitochondria (Honda and Honda, 1999). As expected, *age-1i* increased *sod-3* transcriptional activation while *daf-16*i has the opposite effect (Honda and Honda, 1999; Furuyama et al., 2000) (Figures S6C and S7, Table S15).

**Figure 5:**
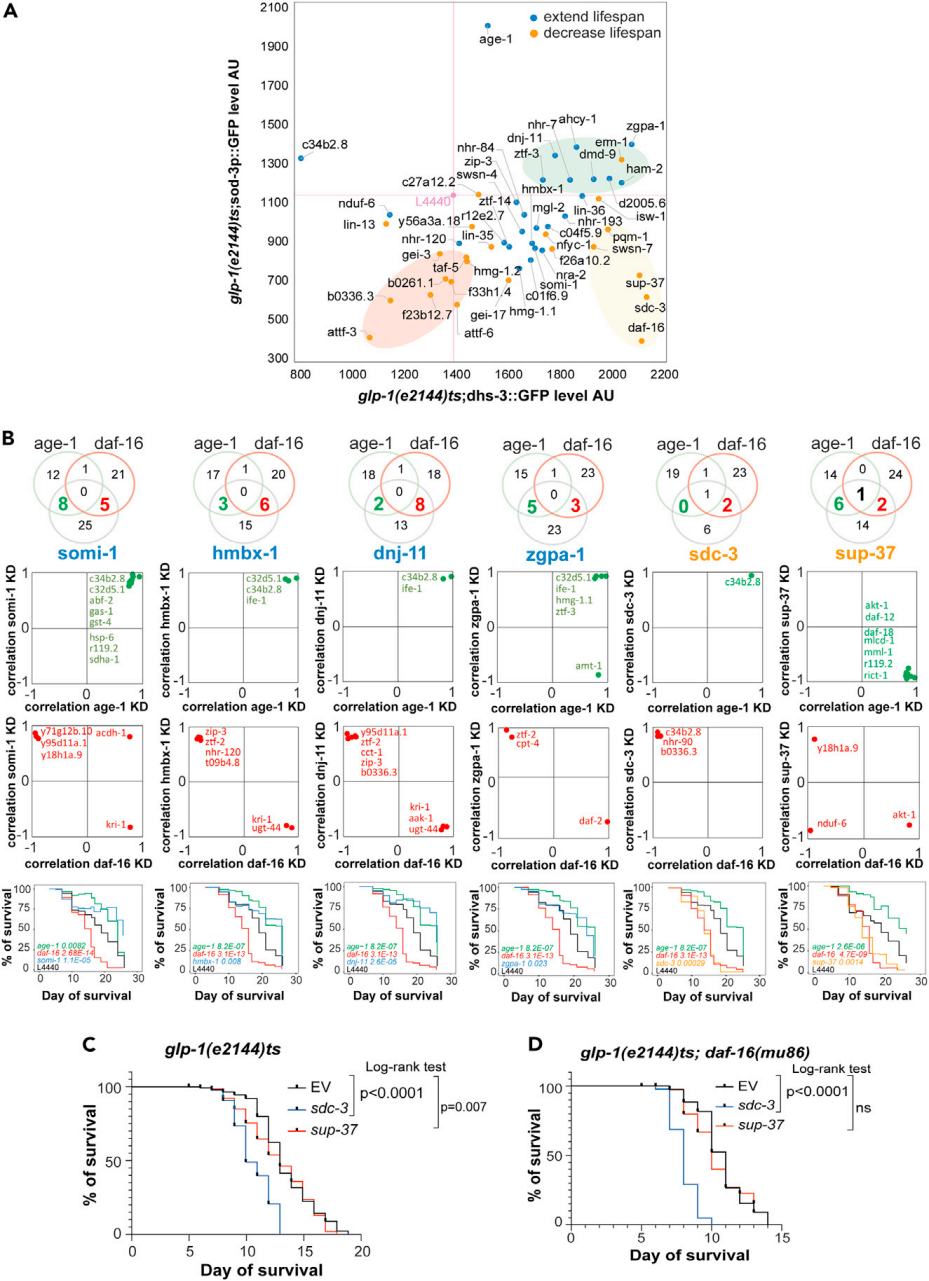
Global characterization of the novel aging genes reveals genes sharing the same metabolic features and pathways as DAF-16/FOXO and ILS (A) Comparison of the *in vivo* fluorescence measures of *sod-3p*:GFP versus *dhs-3p::dhs-3::GFP* in *glp-1ts* animals at day 4 of adulthood. *dhs-3p::dhs-3::GFP* is a translational reporter which localizes to the intestinal lipid droplets (LD). *sod-3p*:GFP is a transcriptional reporter for the expression of superoxide dismutase 3 (*sod-3*), a direct target of DAF-16. Colors correspond to lifespan phenotype as shown in the figure. L4440 is the control/empty vector (Table S15). (B) Novel aging genes sharing known aging and metabolism targets with *daf-16* and *age-1*. The top panel shows Venn diagrams of the number of shared targets of 6 novel aging genes with *age-1* (red) and *daf-16* (green). Aging genes in orange are those whose loss of function leads to a decrease in the *glp-1ts* lifespan, and genes in blue are those whose loss of function decreases the lifespan. Their corresponding survival curves are shown in the bottom panel. The middle panel plots Pearson's correlation between shared targets and a novel aging gene (Y axis) and *age-1* or *daf-16* (X axis) (Table S16). The bottom panel presents the survival curves of *glp-1ts* worms grown at 25°C upon the knockout of the 6 novel aging genes (blue/orange), *age-1* (green) or *daf-16* (red) (Table S12). Control animals fed with an empty vector are shown in black. pvalues were calculated using a logrank test. (C–D) show a lifespan epistasis experiment where *sup-37* and *sdc-3* RNAi was fed to either *glp-1ts* (C) or *glp1ts; daf-16(mu86)* (D) from L1 at 25C. The epistasis was evaluated with a Mantel-Cox logrank test. To evaluate the pvalues of 3 biological replicates we used a Fisher test (Table S20).

We observed a significantly higher *sod-3* transcriptional level in those that led to a longer lifespan than those that led to a shorter lifespan (t-test, pvalue = 1 × 10^−4^; Figure S6C). We also observed a positive correlation between lifespan and LD accumulation, but only up to a certain LD accumulation level; animals with LD accumulation beyond this level have shorter lifespans (Figure S6A). The combined relationship between these two variables and lifespan delineates three groups of genes. The general trend is that long-lived worms accumulate more LDs and display higher *sod-3* transcription levels, similar to *age-1i* (Figure 5A, **green circle**). Interestingly, RNAi treatments that cause uncoupling between fat store accumulation and *sod-3* transcriptional activation, tend to result in shorter lifespan, a group that includes the *daf-16i* control (Figure 5A, **yellow circle**). A third clear group is one that is short-lived, leaner and has lower *sod-3* transcriptional output than controls (Figure 5A, **red circle**). Taken together these results show a systematic relationship between SOD enzymes, and fat accumulation and lifespan in *glp-1(ts)*.

To determine if the intricate relationship also exists in the wildtype context, we analyzed*sod-3* and *dhs-3* signals after RNAi treatments in *fem-3(ts)* animals, and have compared it to the *glp-1(ts)* results (Figure S8). We found that *sod-3* and *dhs-3* signals follow the same pattern in both mutants *glp-1(ts)* and *fem-3(ts)* (Spearman's ⍴=0.466, pvalue=0.0005; Spearman's ⍴=0.89, pvalue=1.47 × 10^−18^ respectively); suggesting that the relationship between SOD enzymes, fat accumulation, and lifespan is conserved. Taken together these results suggest that most of the candidates will have a similar effect in the wildtype context.

To investigate further, we generated a new gene-interaction network based on high-throughput gene expression assays of 19 novel regulators. We also included *age-1i* and *daf-16i* to contextualize our findings. In the *glp-1(ts)* longevity pathway, DAF-16/FOXO activity acts independently of the AGE-1/ILS pathway, which represents a parallel synergistic pathway to the germline longevity pathway (Berman and Kenyon, 2006). We used RNAi to decrease the expression of the regulators and measured their impacts on 177 genes related to aging and metabolism (Table S16). We then used PCC to construct a weighted, directed gene-interaction network (https://s-andrews.github.io/wormgrn/qpcr/).

We first use the network to characterize the interaction of aging genes with several longevity modulators that are key to fat remodeling including *pha-4*, *daf-12*, *nhr-49* and *hlh-30* (Van Gilst et al., 2005; Lapierre et al., 2011, 2013; Wang et al., 2015). As shown in Figure S6B, we have identified several aging genes that show functional interactions with these regulators while influencing lipid accumulation. From this data, we predict the pathway membership of a handful of novel aging regulators including *sup-37*, *gei-17, hmg1.2*, *f26a10* and *somi-1 (*highlighted in Figure S6B).

Using the PCC network, we also analyzed correlations with *daf-2* and *daf-16* (Figure S6E) as well as other known targets downstream of *daf-16* and the ILS component *age-1*. We noticed a group of aging modulators that share a regulatory pattern with either *daf-16* or *age-1* (Figure 5B, Venn diagrams). These modulators encode proteins with diverse functions: nuclear protein (*somi-1*); transcription factors (*hmbx-*1, *zgpa-1*, *sup-37*); heat shock protein (*dnj-11*) and a mitochondrial oxidoreductase (*nduf-6*). The regulatory pattern revealed a striking systematic response. About 60% of the tested genes phenocopy the regulatory pattern of *age1i*, with correlations in the same direction as *age-1i* and in the opposite direction to *daf-16i*. Conversely, some genes -such as *sup-37*- phenocopy the regulatory pattern of *daf-16*, with correlations in the same direction as *daf-16i* and in the opposite direction to *age-1i* (Figure 5B, middle and bottom panels). These regulatory patterns suggest that these genes act as mediators of lifespan through overlapping mechanisms with either *glp-1*/*daf-16* or ILS.

We tested both *sup-37* and *sdc-3* as candidates belonging to the DAF-16/FOXO pathway. While both regulators phenocopy *daf-16i* in terms of fat accumulation and *sod-3* expression levels, according to the PCC network, only *sup-37* shares a high number of key aging and metabolic targets with *daf-16* (Figure 5C; Table S16). To test if *sup-37* and not *sdc-3* work in the same pathway as *daf-16*, we treated both *glp-1(e2131)* and *glp-1(e2141); daf-16(mu86)* with either an empty vector or a vector containing dsRNA against *sup-37* or *sdc-3* from the L1 stage of development. We found that *sdc-3i;glp-1(e2141);daf-16(mu86)* has a significant shortened lifespan compared to the same animals fed with empty vector to (pvalue < 0.0001 on a logrank Mantel Cox test) whereas *sup-37i;glp-1(e2141);daf-16(mu86)* maintains the same lifespan as *glp-1(e2141);daf-16(mu86)* (Figure 5D). This result confirms our prediction that *sup-37* -but not that of *sdc-3-* is dependent on *daf-16*. This encouraging result suggests that the presented data-rich maps are useful tools to guide mechanistic analysis in the context of the germline longevity pathway.

## Discussion

We developed a new “wisdom-of-the-crowds” NI pipeline which successfully extracts meaningful information from noisy whole-worm transcriptomics data, along with integrating a database of 402 orthogonal multi-omics datasets. From the WormExp, modERN, Cis-BP, and GEO databases, we manually identified context-specific datasets, reconciled and curated 380,023 interactions in young adult *C. elegans*, freely accessible to the community. We obtained for the first time, genome-wide GRNs that are contextual to aging in *C. elegans* whose modular structure is biologically meaningful, providing a systems-view of the regulatory interactions underpinning the aging process. This study presents a novel approach that integrates NI with large-scale network analysis tools applied to networks containing many errors at the “local” level, typical of NI-derived networks. Our work provides a compelling example where a network that contains unbiased errors at a local level, can be predictive as long as the global structure is robust. The study led to the discovery of 50 novel regulators of *glp-1(ts)* longevity, augmenting the number of regulators of the pathway by 62.5% and the majority of which have an identifiable human orthologue. This pipeline presents a minimum of 4.8% hit rate, more than a two-fold increase compared to the blind genetic screening in the *glp-1(ts)* which reported a 2.1% success rate (Berman and Kenyon, 2006). Although the fold change may seem modest, we present a pipeline that allows the contextualization of aging modulators in relation to information flow, providing additional mechanistic value to the findings.

Overestimating the number of interactions is unavoidable with current NI tools (Barbosa et al., 2018). Indeed, new methods have been recently developed that may improve future results; non-parametric approaches to correct for noisy transcriptional (Aalto et al., 2020) and the use of context-agnostic prior information, both improve the accuracy of the networks (Wang et al., 2018). Future work using both our comprehensive “wisdom-of-the-crowds” GRN pipeline in combination with other methods that correct for noisy or sparse information may improve overall accuracy of local edges.

We find that the topology of our inferred *input-core-output* network is predictive of functionality in aging. The aging network shows the typical inter-modular pattern of connections and the distribution of key functional roles as classic bow-tie networks. Our GRNs have a thick core similar to the knot of bow-tie architectures of metabolic networks. In these networks, the conversion of heterogeneous substrates to heterogeneous output products cannot be streamlined into a few central reactions, hence the metabolic core is a complex subnetwork of interlinked biochemical reactions. Similarly, aging is a complex process where hundreds of genes and interventions can trigger early senescence in response to a multitude of external factors. In light of this knowledge, it is not surprising that a longevity pathway activates a broad number of health-promoting activities. The challenge in the field is to map this interlinked web of age-modifying genes, most of them located in the core module.

One interesting finding from the structure of the network is the very consistent Gene Ontology enrichment across layers. The top tier layers are enriched by genes that play key metabolic and structural roles, including ATP/energy supply as well as genes involved in the synthesis of proteins. The core is enriched in genes that are involved in transcription, signaling and metabolism and contains many of the key genes that control the long life of germline-less animals. Finally, the outer layer is enriched in genes that modulate global chromatin organization including histones, histone acetylation, and core transcriptional components. The hierarchical distribution where energy (ATP and amino acids) is at the top end of the chain, whereas chromatin organization is at the lower end suggests a model of causality where energy supply constraints and perhaps shapes the landscape of the network, such that the core relays the “available energy” into the global transcriptional status of the chromatin. The role of chromatin in the aging process has been unclear, with some studies suggesting that aging is caused by the progressive opening of chromatin with aging. Our results suggest that the global transcriptional landscape of a long-lived organism is the end result of multiple layers of regulation. But this model may be too broad a generalization because we find some epigenetic modulators (specifically the HMG complex) have a hierarchically distinctive regulatory role at the core of the network.

With regards to the biological insights, this is the first time a DAF-16 target and fat stores have been systematically studied across a large number of aging genes. Based on these relationships, we delineated groups of genes with similar phenotypes and therefore propose lifespan predictor parameters. First is that the transcriptional activation of *sod-3* is positively correlated with longevity (Figures 5A and S6C). Furthermore, these results show that in the control of germline-less animals, *sod-3* is not fully activated and that its expression can be further enhanced. This corroborates the finding that in the germline longevity pathway, DAF-16 is activated independently of ILS. Removal of ILS further extends *glp-1(ts)* lifespan presumably by an additive or synergistic effect on DAF-16 activation (Berman and Kenyon, 2006). The notion that this group of genes may work in the ILS pathway is further strengthened by the results shown by the gene interaction network, where genes in this category share a similar gene expression profile with *age-1i* and *daf-16i* (Figures 5B and S6E).

From our mechanistic studies we have placed the transcriptional activator *sup-37* alongside with *daf-16* regulation. *sup-37* has been previously identified as a DAF-16 target by chromatin precipitation analysis (Wook Oh et al., 2006) further strengthening our findings. It is interesting to also notice that the PCC network also shows that *sup-37* as a positive regulator of *daf-12*, *pha-4* and the PTEN homologue and ILS pathway component *daf-18*, suggesting that it may act as a linker between multiple branches of the germline longevity pathway. Our limited but encouraging validation strategy indicates that data-rich maps are powerful tools to guide mechanistic predictions. Future work should be done to establish if the other predicted interactors of ILS- including the transcription factor *zgpa-1* and the heat shock protein *dnj-11*- work in combination with the *daf-2* pathway to extend *glp-1(ts)* lifespan.

Second, our results show that we observe that an increase of fat accumulation is positively correlated to lifespan up to a threshold. Beyond a certain amount of accumulated fat, if *sod-3* is not concomitantly activated, animals have a shortened lifespan (Figures 5A and S6B). This relationship is expected, since fat metabolism causes an increase in mitochondrial reactive oxygen species (ROS) production and SOD enzymes are known to limit ROS toxicity (Ighodaro and Akinloye, 2018). We have also made a number of new mechanistic predictions linking longevity genes that are involved in fat remodeling and autophagy (Figure S6B, such as *pha-4*, *daf-12*, *hlh-30* and *nhr-49*). Additional work will be required to establish the validity of these connections.

Our approach is the first that attempts to tackle aging modulation by fully embracing its systems-wide complexity in *C. elegans*. We have shown that despite the noisy transcriptomics data and the shortcomings of available NI methodologies, systems approaches are able to extract biologically meaningful information contextual to aging. Future studies drawing upon our work will be able to refine and expand our findings to other model organisms as well as humans.

### Limitations of the study

Although the large-scale organization of the network is informative about the location and overall role of aging modulators, the network is to noise to yield high quality information about specific targets of these regulators that help fully map aging pathways. We still need to develop NI tools that are able to deal with complex regulation data and provide locally reliable gene-regulatory networks. Finally, our results are context dependent (i.e., valid for glp-1 animals), up to what extent our results exactly translate to wild-type animals will require further experiments.

## STAR★Methods

### Key resources table

**Table:** 

REAGENT or RESOURCE	SOURCE	IDENTIFIER
**Chemicals, peptides, and recombinant proteins**		

N, N′-dimethyl-4,4′-bipyridinium dichloride	Sigma	Item No. M2254; CASNo: 75365-73-0
L-(−)-Dithiothreitol	Sigma	Item No. D9760; CASNo: 16096-97-2
3-oxo-cholest-4-en-26-oic acid/ Δ7-Dafachronic acid	Cayman Chemicals	Item No. 14100; CASNo. 23017-97-2
C_20_H_31_O_2_⋅ Na Arachidonic acid	Cayman Chemicals	Item No. 10006607 CASNo. 6610-25-9
N, N′-dimethyl-4,4′-bipyridinium dichloride	Sigma	Item No. M2254; CASNo: 75365-73-0

**Critical commercial assays**		

Power SYBR Green Cells-to-Ct kit	Invitrogen	Item No. A25599
96.96 Dynamic Array IFC	Fluidigm	Item No. SKU 100-6173

**Deposited data**		

Time-series RNA-seq	Generated in house	GEO accession: GSE166512
DAF-12 ChIP-ChIP datasets in young adult worms	Hochbaum et al. (2011)	GEO accession: GSE28350
HSF-1 ChIP-seq datasets in young adult worms	Li et al. (2016)	GEO accession: GSE81521
Curated physical prior dataset	Raw data taken from public databases	Zenodo repository https://zenodo.org/record/4382337#.YA6jculKhhE
Network related and experimental datasets	Generated in house: TF-gene interactions valid in young adult C. elegans (day 1 until day 4 adulthood)	Zenodo repository https://zenodo.org/record/5499464#.YA6h1OlKhhF
Gene interaction database based on qRTPCR experiments	Generated in house: 5,497-edge gene interaction network (both positive and negative edges) from 19 novel ageing genes and 10 randomly selected regulators (Tables S7 and S16)	Zenodo repository https://zenodo.org/record/5499464https://s-andrews.github.io/wormgrn/qpcr/

**Experimental models: Organisms/strains**		

*glp-1(e2144)III*	Caenorhabditis Genetics Center	WB strain:CF1903
*fem-3(q20) ts IV*	Caenorhabditis Genetics Center	WB strain:JK816
*ldrIs1 [dhs-3p::dhs-3::GFP + unc-76(+)]. dhs-3::GFP*	Caenorhabditis Genetics Center	WB strain:LIU1
*muIs84 [(pAD76) sod-3p::GFP + rol-6]*	Caenorhabditis Genetics Center	WB strain:CF1553
*glp-1(e2144)III; ldrIs1 [dhs-3p::dhs-3::GFP + unc-76(+)]*	This study	strain:MOC269
*fem-3(q20) ts IV; ldrIs1 [dhs-3p::dhs-3::GFP + unc-76(+)]*	This study	strain:MOC267
*fem-3(q20) ts IV; muIs84 [(pAD76) sod-3p::GFP + rol-6]*	This study	strain:MOC274
*glp-1(e2144ts) III; muIs84 [(pAD76) sod-3p::GFP + rol-6]*	This study	strain:MOC313
*rrf-3(pk1426) II, glp-1(e2144)III*	This study	strain:MOC308
*glp-1(e2141)* III	Caenorhabditis Genetics Center	WB strain:AGD1032
*glp-1(e2144)III*	Caenorhabditis Genetics Center	WB strain:CF1903
*fem-3(q20) ts IV*	Caenorhabditis Genetics Center	WB strain:JK816
*ldrIs1 [dhs-3p::dhs-3::GFP + unc-76(+)]. dhs-3::GFP*	Caenorhabditis Genetics Center	WB strain:LIU1
*daf-16(mu86); glp-1(e2141)*III	Caenorhabditis Genetics Center	WB strain:AGD1048
glp-1*(e2141ts) III; adls2122 [lgg-1p::GFP::lgg-1 + rol-6(su1006)]*	Caenorhabditis Genetics Center	WB strain: MAH42

**Oligonucleotides**		

OL010 R sma-10 CCGGTCTTGGAGTTCCTGTG	All oligos were designed in house	Does not apply to any oligos in this list/ NA
OL011 F bcat-1 TCCCGGAGCAAAAGTTCTTCA		
OL012 R bcat-1 TTCTGGACGGAACATGCGAA		
OL015 F elo-1 ACACGAAACGATTTGTGGCTA		
OL016 R elo-1 AGGATTGAAGCCTGAATAGTAACAT		
OL017 F ech-8 GGCTCAGTGGTCTCTTCCAAAT		
OL018 R ech-8 GCGATTGCAATTCCTCTTCCC		
OL009 F sma-10 AAGTTGCAAGTCTACCAAGCG		
OL019 F gba-4 GGATTTGGAGCTGCATTCACTG		
OL020 R gba-4 CCCGAGACCATCATCGGAAAA		
OL021 F ttx-3 AGGGTTCTGCAGGTTTGGTT		
OL022 R ttx-3 ATTGATGCCAATGGGGCAGA		
OL023 F asp-3 ACGATGTTGTCTGCTTCGGA		
OL024 R asp-3 AGCGACGAAGGTGATTCCTG		
OL025 F acs-7 ATCGGGTACAACTGGAAAACCA		
OL026 R acs-7 CGTTGGCATCGAAGAATCTCA		
OL027 F lpr-3 TAGGACAGGTTGTCCCACCA		
OL028 R lpr-3 GGGAGGGCAATTGGTTGTTG		
OL029 F lin-3 ACTACTGTCATCACAACGCGA		
OL030 R lin-3 AACCCTGTGGACAATGGCAA		
OL035 F best-17 CTGGATGAAGGTGGCAGAGG		
OL036 R best-17 ATCGCCATGCCTCTCGAAAT		
OL041 F daf-2 GGAAGAAGAGAATCTCGGCCC		
OL042 R daf-2 CGAGCTTCGCTGCTGCTTA		
OL043 F nduf-6 CGGACAAGCGTGGGATCAAT		
OL044 R nduf-6 AATCCTCTGGAGGGCGTTGA		
OL047 F nuo-6 TTCTTCGACCAGCTGCCAAC	All oligos were designed in house	Does not apply to any oligos in this list/ NA
OL048 R nuo-6 GCAGAAATTCGGTCTTTCCGTG		
OL051 F nhr-62 (Ver.2) ACCTGGTATGCGTAGTCTGC		
OL051 F nhr-62 (Ver.2) ACCTGGTATGCGTAGTCTGC		
OL052 R nhr-62 (Ver 2) CCGGTTGTGCCAAACACTTC		
OL053 F nhr-17 (Ver.2) TCATCCACACGGCGTTTTCT		
OL054 R nhr-17 (Ver.2) AGCAACGT TTCGAAATCCACA		
OL55 F abu-11 AGGAGAATGTGTTCCTCCGC		
OL56 R abu-11 GTTGGTTTGAGCTGGTTGGC		
OL57 F fmo-2 TCGACATGGTCTTCTCTATGGC		
OL58 R fmo-2 TGACGACTCATTCGTTTCGTG		
OL61 F lpr-5 GGAGAAGCCACCGGATCAAT		
OL62 R lpr-5 CCCTTTCCTTTCACCAAGGC		
OL69 F gipc-2 GCCGTTCTAGAAGCCGATCA		
OL70 R gipc-2 TGAAGCTAAAACGGCAGGCT		
OL71 F nhr-76 TCGTAACGCCGTAGTGATGG		
OL72 R nhr-76 CGAGAAGACCAGCCTCATGT		
OL73 F cup-16 AGGAAGATCGCATCAGTTGGG		
OL74 R cup-16 AGAGAGCACTTAACGGCTTCAA		
OL75 F tatn-1 CGTATTACCCCACTTCCGGG		
OL76 R tatn-1 GATCCTTGCGGTTTGGCTTG		
OL81 F tre-5 CGAGCTAACGCAATTGACCG		
OL82 R tre-5 CATTGTCCAGGCGCTGTTTC		
OL85 F mak-1 AGCTATTGCCATTCCTGGAGC		
OL86 R mak-1 TTTCGGCAACCGTATCAGGA		
OL87 F hsp-70 CGATGAAGTTGTCTTGGTTGGG		
OL88 R hsp-70 TGGAGCAGTTGAGGTCCTTC		
OL89 F tnt-4 TTGAATGAGCGTTCCCGTCA	All oligos were designed in house	Does not apply to any oligos in this list/ NA
OL90 R tnt-4 TGGACCTTTGGTGGATGTCTT		
OL91 F nhr-23 GCCAATACTGCCGTCTCAAA		
OL92 R nhr-23 TGCATTCGAACCTCATCTTCT		
OL93 F aagr-4 ACCAAGCAGGAGCTTTCCAG		
OL94 R aagr-4 AGCTTCAGTTGTGTTGTCTGC		
OL97 F pqn-13 AACTTGCGGACAACAAGCTC		
OL98 R pqn-13 AGGTTTGCTGGCACTGTGG		
OL99 F faah-2 GTATCGCCAGCTCTTCCACA		
OL100 R faah-2 ACAGATCCTGCAGCATAATCCA		
OL101 F abu-13 GTGAAGCAATGTCGCAAGGG		
OL102 R abu-13 GCTCGGCGAGTTCTCTATCC		
OL107 F F56F10.1 CCTCCATTCGATGCAAACACA		
OL108 R F56F10.1 TGTGCTGTTGCTCTGTCCAT		
OL109 F clec-186 AGCCTGTCCACAAGGATTCG		
OL110 R clec-186 CGGAGCATCATCCGGTACAT		
OL111 F nas-36 AGCAAAATCTACCGATGCCGA		
OL112 R nas-36 CACCCCAATCTGCCCATACA		
OL115 F dlat-1 CGCCAAAAGCTCAGGACTTG		
OL116 R dlat-1 TGCTTTGGCAGATTTCCACTG		
OL117 F cnp-3 CACTTCCAATGCCACCACAATC		
OL118 R cnp-3 ATACGGCATCATTGGAGGCA		
OL119 F R08B4.3 GCAGTCGTCATATGGGATGGT		
OL120 R R08B4.3 AGCGTATCGACGTAGTCTGC		
OL121 F Y14H12B.1 TGCACATGGGATGGTTGTGA		
OL122 R Y14H12B.1 CCGCGTCGATGGAATCTCAG		
OL123 F mccc-1 GCAGGTACCGTCGAGTTCAT		
OL124 R mccc-1 AGATCCGTGCCTGTGATAGC	All oligos were designed in house	Does not apply to any oligos in this list/ NA
OL125 F C25H3.9 AGGACTATCCAACAGAACCACA		
OL126 R C25H3.9 TTCGTGGTGCTCAACATCCG		
OL127 F dpyd-1 AGATGACAACGGCAAGTGGT		
OL128 R dpyd-1 AAAGCCGACAAAACTGCGTC		
OL129 F mlc-3 CGGACAGGAGTTCAAGCGTA		
OL130 R mlc-3 GGTTCCTTGCTCCTTCTCCT		
OL131 F aqp-7 TATCTTCGGAGCCTGGTCCT		
OL132 R aqp-7 GTCGGTGGCAAGTTTCAGTG		
OL133 F K12C11.1 TGCTAGTAAAATCGCGTCGGA		
OL134 R K12C11.1 TGTGACGGAACAGACTCTCC		
OL135 F fbxa-85 GCTGATCCCAATCCAGAGATTAGT		
OL136 R fbxa-85 TTGCCATGCTCAATTGCGAG		
OL137 F unc-10 GCCAATGTTGGCTTCCAGTG		
OL138 R unc-10 TCTCCTAGCACCGGTGAGG		
OL141 F phb-2 CACTCAACGCCAACAGGTTT		
OL142 R phb-2 GCTCGGTGAGGGAAACATCA		
OL143 F hpl-2 CCACCGGGACATCGAATTCTT		
OL144 R hpl-2 CGGTTTGCGCTTCAGTCATC		
OL145 F prdx-3 TCTGGAACTGTCCGTCACAC		
OL146 R prdx-3 GGCAAACCTCTCCGTGCTTA		
OL147 F haf-9 AGGAAGCCAAATGTCAGGAGG		
OL148 R haf-9 TCAGCGTCCAAAGCAGATGT		
OL149 F nuo-5 GAATGAGATCGCTCCCCACC		
OL150 R nuo-5 GGGAGACGTCAACGTCGATT		
OL153 F egl-9 GTGTACATATTGTGGAAGCTCGTG		
OL154 R egl-9 CAATCGAGTTGCTGGTGCTC		
OL155 F grl-16 AGACCAACGAAGCTGTCGAG	All oligos were designed in house	Does not apply to any oligos in this list/ NA
OL156 R grl-16 TCGAGGTTGTCCTTCTCGTTC		
OL157 F coq-5 AGACATTCGCCTACCGCAAA		
OL158 R coq-5 CGCGAGATGGTTGGATATCTCT		
OL159 F asp-14 CAACCAAGCAAAGTCGTCGT		
OL160 R asp-14 CGGTCTCCGATCACAACAGT		
OL161 F Y69A2AR.18 CAACCTCAGAGCTGCCTGC		
OL162 R Y69A2AR.18 GGAAAATTGGGGCGTCGAAA		
OL163 F abu-10 TATTGTCGCCCTGGCACTTT		
OL164 R abu-10 GATGGTGGATTGTTGGCAGC		
OL165 F rmh-2 GATGGATGTCACCCGTCGAA		
OL166 R rmh-2 GCAGGACACTGGTTCCTCAT		
OL167 F immt-1 ACGAGCTCTTGTGGAAAGCC		
OL168 R immt-1 GAGTCCGGATGACTTGCTCC		
OL169 F f17h10.1 AGCTTACAGACCCGATCCGA		
OL170 R f17h10.1 TGGCGAGAAGACCTCCCATA		
OL171 acd-1 F ACTGATGGACACATGAGAGCAA		
OL172 acd-1 R TCACTGAGAAGTTGCGTGACA		
OL173 ahcy-1 F CTGCAACGTTGGTCACTTCG		
OL174 ahcy-1 R GTAACGGTCAACCTGTGGCT		
OL175 art-1 F ACAAATCGCCTGGAAGACTGT		
OL176 art-1 R GTAGCATTGACAGCGGCTTG		
OL177 atp-2 F AGTCGCTGAGGTGTTCACTG		
OL178 atp-2 R GGTGGTCGAGTTCTCCCTTG		
OL179 asp-17 F TGGGGTCACTTATGTTCCGC		
OL180 asp-17 R CCGTGTCGGAAATTACCTGATTG		
OL183 bli-6 F GGCTTCAGAAATGGAGGTGGA		
OL184 bli-6 R CGGAAGTGTGATACATACCGAAAG	All oligos were designed in house	Does not apply to any oligos in this list/ NA
OL185 clec-265 F ACACAAACTTTGCAGTTGGAGA		
OL186 clec-265 R AGAATCTGGGCATGGCTGAG		
OL187 bli-1 F TGCCCAGAACTATCCAAGGTTT		
OL188 bli-1 R CCGGCTCTCTACCGTAATTGT		
OL189 clec-75 F GGTGTTCAGCCAATCTCCGA		
OL190 clec-75 R GCAAACGGCACTAGATAACACA		
OL191 col-109 F GATCGCTGGAAACCTGACCA		
OL192 col-109 R AGGCTTTCCGTTACGTCCTG		
OL193 dld-1 F GTCGCTGTCCAGAACGACT		
OL194 dld-1 R GACGAGATCGGCATCTTGAGT		
OL195 dod-19 F TGGGCAGAAAACACTCCTTGA		
OL196 dod-19 R GCTGTAATCAGATAGGCGGACT		
OL197 fkb-1 F GCGTTCAGAAATCCAGAAAGGG		
OL198 fkb-1 R ATTCTTCATTGCGAGTGCGG		
OL199 folt-2 F GGCCACAGTTTGTGGTCTATTC		
OL200 folt-2 R CGTTTTGAAAAACCGCCTGC		
OL201 grsp-2 F GCTCAGGGATCCCCAGGTC		
OL202 grsp-2 R CCATCCTCCAGATGAACCGA		
OL203 lec-10 F TATCACAACCCCGGTGTCC		
OL204 lec-10 R TTCTTGTGATGTCCGTGACGA		
OL205 lips-15 F GGGTCAGCAACCTTGAATGC		
OL206 lips-15 R GTGTTCGGGTAGTGGGACTG		
OL207 lpd-5 F CTGGAACTGCGGATCCACTC		
OL208 lpd-5 R CTCCTCGACGTCAAACTCCC		
OL209 dpy-13 F ATCGCCGTGCTTTCGGTTT		
OL210 dpy-13 R CTTGCAGGTAAGGGCTTCGT		
OL211 ell-1 F ACCAGCTTCTAAGCTCAACCC	All oligos were designed in house	Does not apply to any oligos in this list/ NA
OL212 ell-1 R AGTGTCACGTGATGGTGGTG		
OL213 mec-12 F TCGGAGGATCCGATGACTCT		
OL214 mec-12 R GCGGATTTCGTCAATGACAGT		
OL215 mec-7 F GCAATCAGCAGTATCGTGCC		
OL216 mec-7 R CAGCGGTGAGATAACGTCCA		
OL217 mrpl-32 F ACTGCACAAAACTTGCCAATGA		
OL218 mrpl-32 R GCGAGTTACTTTCTTTGGTTTGC		
OL219 mrpl-4 F GGACTTTGTGTTGCTCTCACTC		
OL220 mrpl-4 R GAGCTTCGCAAAGATCAATCCA		
OL221 mrps-26 F CTGCCATGTCACAAATGGACG		
OL222 mrps-26 R TAGAGAACT TTCTTCGATGGAGGC		
OL223 mrps-30 F AGGAAGCTAAACAAATGCTCGAC		
OL224 mrps-30 R TGAATCCATGAGTGTGAGCTTGA		
OL225 oxy-5 F TCGGCCCAGTTGGAAAGATT		
OL226 oxy-5 R AGCCAGTATTAACAGCCTCACA		
OL227 pat-10 F CCGACAACATGGCTGAGGAT		
OL228 pat-10 R GCTTTCCTCTGTCGAAGGCA		
OL229 rack-1 F GAAGCTTACCGGAACCCTCG		
OL230 rack-1 R GTCTTGTCGCGGGAAGATGA		
OL231 rla-0 F GCTTGTCGAGCTTTTCGAGG		
OL232 rla-0 R TCAGCATGTCCTCTCATGGC		
OL233 rol-8 F ACGGTAGAGATGCTGAGGTT		
OL234 rol-8 R ATTGGTCCTATAGCTCCCATTG		
OL235 rpl-3 F GGATACGTCGACACCCCAC		
OL236 rpl-3 R CTTGGATTTAGCCCAGTTGCTG		
OL237 rps-11 F GTCGTTCGTCGTGACTACCT		
OL238 rps-11 R GTGGATATCTCTGAAGGCTGGG	All oligos were designed in house	Does not apply to any oligos in this list/ NA
OL239 rps-28 F AGAAACGTTAAGGGCCCAGT		
OL240 rps-28 R AGTCGAGACCGACACTTAGC		
OL241 rps-9 F CAGGCTCCAAACCCAGGTC		
OL242 rps-9 R TTGTCTGCGGACACGGATG		
OL243 sdha-2 F AGGAAGAGGAGTCGGACCAA		
OL244 sdha-2 R GGTTTCCGAGATTCCTGGCA		
OL245 sol-2 F TGCAGTGGAGGGTCTGATTG		
OL246 sol-2 R TCCATGACCCTGTTCAACACA		
OL247 taco-1 F AGAAGGGGCACTCGAAATGG		
OL248 taco-1 R CTGCTCCACGGACCTTTCTG		
OL249 tfg-1 F GCCAACGACTTGACACTCATC		
OL250 tfg-1 R TGTCACCAAATCGCCTTCTTC		
OL251 trap-1 F GCCAATGTTGTTCGCGAGTT		
OL252 trap-1 R GTCTCAGCGTACTTCACGACA		
OL253 tufm-1 F ACAAAGATCCTCGCCACATCA		
OL254 tufm-1 R TTGATGGTGATACCACGGGC		
OL255 ugt-25 F CAGTACTAGACGAACGACCACA		
OL256 ugt-25 R CTCCAGCAGTCCATTTCTCCA		
OL257 unc-62 F GGCATCCGATGGAGGGATCA		
OL258 unc-62 R GTCCCCTGCTTGGTTGGAAG		
OL259 unc-69 F AGTGCTCGGCCAGTACATT		
OL260 unc-69 R AAAATCGTCGTCGTGAATGCT		
OL261 vdac-1 F CGGATGGAAGGTTGGTGGAA		
OL262 vdac-1 R CAACGGCAACTTGGGAAGAG		
OL263 vhl-1 F TGGCTGAATCCATCAAAACAACC		
OL264 vhl-1 R CTCCTAGCAACCCATGGATGA		
OL265 trp-4 F AATAAGAAAAGCGCCACGCC	All oligos were designed in house	Does not apply to any oligos in this list/ NA
OL266 trp-4 R GGGTCATGCATCTCAACCGA		
OL267 vap-1 F CGGCTGCTCTAACACCAAGA		
OL268 vap-1 R ACTTGTTTGGCTCAAGCCCT		
OL269 mct-4 F GGCGGGAGGATTGATCTCTG		
OL270 mct-4 R CAAGACCTCCCATCACTCCG		
OL271 gst-10 F ACTTCACTATTCGAGGATTCGGA		
OL272 gst-10 R CTTGCCATTCATTCCCCTCG		
OL273 sams-3 F CGCTGAGAACGGACACTTTG		
OL274 sams-3 R CCGTTTGAGATCGTTTTTCCTCC		
OL275 B0491.5 F CGTGGCTACCATGAAGAGCA		
OL276 B0491.5 R TTGGAGTCCAGTCTCCGAGT		
OL277 C12D12.1 F CCGTTACAGTGCCGACTACA		
OL278 C12D12.1 R TGGTTTGCTTACTTGTGCCA		
OL279 C17F4.7 F TTTGCACACAATTGCCCGTT		
OL280 C17F4.7 R TAGGGCTTATGCGCAGCC		
OL281 C18D11.1 F TCGGATTGCAGTTCCTCCAC		
OL282 C18D11.1 R CCACGGACGTCGTACACTTT		
OL283 C40H1.8 F ATTGGTCTGTACAAGCCGGA		
OL284 C40H1.8 R TGTACGGGAAAGTTTTGGTATGC		
OL285 C55A6.7 F GTAACCGGATCCAATCGAGGA		
OL286 C55A6.7 R TGAGAGCAGTTGCCTTATCAAC		
OL287 dlat-2 F ACTGAGGTCCGTGCCGA	All oligos were designed in house	Does not apply to any oligos in this list/ NA
OL288 dlat-2 R GGAGTGATAAGGCCGGTTGG		
OL289 C55B7.3 F TGTGACTCTGAGACTGCTCCA		
OL290 C55B7.3 R ACACTTGTTGAGAACCGAGTGA		
OL291 cpt-3 F GTTGGCGAAAATCGTCTCCG		
OL292 cpt-3 R CGGTTTTTGCTCCATTCGGT		
OL293 dct-8 F GCTTCCTTCGCAACCTCATTG		
OL294 dct-8 R TTTCCATACATATTTCCTCCGGCA		
OL295 eef-1A.1 F ATCACTGGAACATCCCAGGC		
OL296 eef-1A.1 R CGCGAGTTTGTCCGTTCTTG		
OL297 F23C8.5 F TGCTGTCAAGATCCGTGTCAA		
OL298 F23C8.5 R TTGACGGCCTCCTCTAAAGC		
OL299 F26G1.2 F GAAGAACGCGAGAAAGTGCG		
OL300 F26G1.2 R CGTTGGATTCTTGCCCGATG		
OL301 F33G12.6 F CTGCTGACGACATAAGAGGTGA		
OL302 F33G12.6 R CGATTCGATCCCAACATGCTTC		
OL303 fkb-7 F TATGCCAGGACTTGATAAGGGTC		
OL304 fkb-7 R TGCTCTTGCTCTTTCTGCTCA		
OL305 brp-1 F CTTCAAGAAAACCGAAACATCCG		
OL306 brp-1 R TCCTGGATTTTCCGCAGAGTT		
OL307 T19C9.8 F GTGTAGCTAGTTACCATGCTGGA		
OL308 T19C9.8 R AACTTGGATTTGGTACCGGTGT		
OL309 T21H3.1 F CCGTCAGTCCACAGGACAAG		
OL310 T21H3.1 R GCAGCCCATGGAGTGTGAT		
OL311 Y26D4A.21 F AAAATGTAAGAT TCATCCAACCGAC		
OL312 Y26D4A.21 R CTCGTCCGATTGAATTGCCTG		
OL313 Y51H7C.13 F GCTATTGGAACCAAGTGCTGC		
OL314 Y51H7C.13 R TTTGGGATGGAAGGTTCGGG	All oligos were designed in house	Does not apply to any oligos in this list/ NA
OL315 Y54F10AM.5 F TCTTTGAAGCGTTCCTGCCT		
OL316 Y54F10AM.5 R TGGTCGTCATGGCACTTTGA		
OL317 Y51H1A.3 F GGAATTGGGGAGAGCTCGTG		
OL318 Y51H1A.3 R CGATCGAGTTCCAGGTGGTG		
OL319 Y71G12B.10 F GTTATGAGGTCTCGCTGGGC		
OL320 Y71G12B.10 R GTTTTTCGGCTGGCACACTT		
OL321 Y43F8B.1 F CGAGCAACTAGCCCAGAAGT		
OL322 Y43F8B.1 R GAGCTCGGAATCAGCTACCC		
OL323 lpl-1 F AGGTGTTAGGCATTTTGTGGAC		
OL324 lpl-1 R ACCCATACACCTGTATTCGCA		
OL325 atp-1 F AAACCGGAAAGACCGCCATT		
OL326 atp-1 R GACAGCGACGTAGATGCAGA		
OL327 mdh-2 F CTTCCAGCAAAGACCCTCGT		
OL328 mdh-2 R AGAAGAGCGACCTTTGGAGC		
OL329 paf-2 F AGTTGGTCATGTCATCCGCT		
OL330 paf-2 R TTGCTTTTTGGAAGTCCGTTGT		
OL331 str-7 F TTTCACATCAAACGGCAATTCG		
OL332 str-7 R GGAGGAACGTGTGAAACAAGTAT		
OL333 tag-120 F TATTTTCACTCTCTCGGCAGCA		
OL334 tag-120 R TCCACTGCATACTGTGGTGAT		
OL335 sucl-1 F GGGAGCTGCTCGTTTCTACA		
OL336 sucl-1 R AGGTACCCTGCTTTCCTGTG		
OL337 tald-1 F GAATTCGGGCTGCTAACACG		
OL338 tald-1 R GGCGAGATTAGGGTGACTCC		
OL339 tiam-1 F CCTTGTGATGAGCAGCCAGA		
OL340 tiam-1 R ACGCGAAACATTCCAGCAAAT		
OL341 rhy-1 F TGACACTTGTCGTCATCGGAA	All oligos were designed in house	Does not apply to any oligos in this list/ NA
OL342 rhy-1 R TTCGAGGATCTTTCAGTGAGCA		
OL343 dpy-2 F GAATTTTGTCAGGCATCCGCA		
OL344 dpy-2 R ATCCAGCAGCCCTTTTCGTT		
OL345 C16C10.4 F TGCATA CTTGGATGGACTGTTCT		
OL346 C16C10.4 R TCTGCCTGCACAATAGCGAA		
OL347 glf-1 F CCGTGTCACAATTCTCAGCAG		
OL348 glf-1 R TCTCTTCCTCGGTGATCGGA		
col-129 F TCAATGATGTCAACAATTACTATGATGA		
col-129 R GCCAGACGGTATTAGCATCG		
OL335 sucl-1 F GGGAGCTGCTCGTTTCTACA		
OL336 sucl-1 R AGGTACCCTGCTTTCCTGTG		
OL337 tald-1 F GAATTCGGGCTGCTAACACG		
OL338 tald-1 R GGCGAGATTAGGGTGACTCC		
OL339 tiam-1 F CCTTGTGATGAGCAGCCAGA		
OL340 tiam-1 R ACGCGAAACATTCCAGCAAAT		
OL341 rhy-1 F TGACACTTGTCGTCATCGGAA		
OL342 rhy-1 R TTCGAGGATCTTTCAGTGAGCA		
OL343 dpy-2 F GAATTTTGTCAGGCATCCGCA		
OL344 dpy-2 R ATCCAGCAGCCCTTTTCGTT		
OL345 C16C10.4 F TGCATACTTG GATGGACTGTTCT		
OL346 C16C10.4 R TCTGCCTGCACAATAGCGAA		
OL347 glf-1 F CCGTGTCACAATTCTCAGCAG		
OL348 glf-1 R TCTCTTCCTCGGTGATCGGA		
col-129 F TCAATGATGTCAACAATTACTATGATGA		
col-129 R GCCAGACGGTATTAGCATCG		
acdh-11 F TAATGCAGTCGCATCGTTGG		
acdh-11 R CCATTTGGACTGTGTTTGCCC	All oligos were designed in house	Does not apply to any oligos in this list/ NA
acdh-1 F TCCGAGCTTCATCCACTTGT		
acdh-1 R TGCGTATTTGTAGCCTTTTCCA		
acdh-8 F CCGCAGCTGAAGTTGACTCT		
acdh-8 R CCTCCGAAGATCTGACAAGCA		
acs-5 F GGGAGAATACGTGGCACCAG		
acs-5 R AGCGATGAGCCATCTTTCCA		
aip-1 F CTGGACGGATTCAATCACATC		
aip-1 R TTGTAGAGAACGAGCCAGAGC		
atg-18 F AGCCGCAAGGAGTAATCAAGTATC		
atg-18 R TCCGTCTGATGTAGCAGCCAT		
cct-4 F CAGCCACGACGATAATACAACAG		
cct-4 R CAGGCGGTAGAGCAGGAAGT		
cpt-4 F CGAGAACAGAGACGCTACGC		
cpt-4 R TTCAACAGGTCTCTTCGCTCTT		
cth-1 F GGAGCCGCTATCACTAATAATGAC		
cth-1 R CGAATGGAGATGGAACACCT		
dnj-11 F AGCAAGCCGACAAGGAGACA		
dnj-11 R CATCTCTCCACGGTTCCAGGT		
dod-17 F ATTCACACTCACTGTCGCTAACG		
dod-17 TCGGTCCTGTGCTGTATTCGR		
ech-6 F TCTATGCCGGAGAGAAGGCT		
ech-6 R AACGCTGAGTACCTCCTGCT		
elo-5 F ATGCACTGGTACCATCACGC		
elo-5 R ACCCAAACCATATGGACAGCA		
ets-4 F TCTCAAAGGACGCCGATCAC		
ets-4 R CTGGGTGTCAAGACCGTTGT		
fat-2 F GTCCCGGCTCTTCGAGACTA	All oligos were designed in house	Does not apply to any oligos in this list/ NA
fat-2 R GAGGACAAAGGCAATGTAAGCA		
fat-4 F AGTTTGCATTGAGCTCGAACAT		
fat-4 R CCTCCCCAAAGCCAGTCAAT		
fat-7 F GCCGTCTTCTCATTTGCTCTC		
fat-7 R CTCATTGGTGTGGTTGCCTT		
lbp-8 F GAGAGAAATTTGTTGAAATTGCTCCG		
lbp-8 R TGAAAACAGAGCTGTGGTGGT		
lbp-9 F TCGTGATGTGTCGAGCGTC		
lbp-9 R ACGATGACGAGCTTTCCGTT		
let-767 F GCAGCTGTGGCTTATCGTCT		
let-767 R CGGTGACAACAGCCCAAGAA		
lipl-4 F TGATGACTGTAATGATCCCATTGT		
lipl-4 R CCATGATTTTATTAATTCCGGCGTA		
mex-1 F TCGCAGAGCCACCAACAAGA		
mex-1 R GATGAGGAAGAGGACCGATGC		
mtl-2 F TGGTCTGCAAGTGTGACTGC		
mtl-2 R GGCAGTTGGGCAGCAGTATT		
nhr-80 F ATCACCGACGAGATCATGCC		
nhr-80 R TCGAAACCCCCTTGAAAGC		
oma-1 F CCAAGATATGAGCTACCAACGAA		
oma-1 R CAGCGAGACGGTGGATAGGT		
sbp-1 F ATCCGATTGGATTGCTCGCT		
sbp-1 R GAGTGCTAGTTCCATCCGGG		
sip-1 F CGGGTTCAGCAAGAGATCGT		
sip-1 R CCAAGTCGACGTCCTTTGGA		
tcer-1 F TGAGCCACGAAAATCAGGAGAA		
tcer-1 R CTCTACCTCTGCCGCCAAAA	All oligos were designed in house	Does not apply to any oligos in this list/ NA
vit-1 F AGCCAGAAGAAATCCGATCTTG		
vit-1 R GCTCCACAGCTTCGTATCCA		
vit-2 F TCCATCAAGAGCCACATCAAGA		
vit-2 R CGAACTCAGCCTTGTCTCCA		
cdc-42 F TCCACAGACCGACGTGTTTC		
cdc-42 R TCCACAGACCGACGTGTTTC		
pmp-3 F gttcccgtgttcatcactcat		
pmp-3 R acaccgtcgagaagctgtaga		
ire-1 F TACTTGCCACCACGGAGACC		
ire-1 R CGTTGCCATCGTCATCATTG		
OL001 F mdh-1 GAACCAAGGCTGGGCAATTC		
OL002 R mdh-1 ACCCTCGATGGTAACTGGGA		
OL003 F sams-1 CCAGCATTGGATTCGACCAC		
OL004 R sams-1 TCCGACATCTTCTCCGTCCT		
OL005 F far-3 GAGCTCATTGCTGGAGGACG		
OL006 R far-3 TGCAGCAACTTGGGTTTCAAT		
OL007 F sma-4 ATATCCGTTATTACCTCAAATGCCA		
OL008 R sma-4 AGAAGACGCTTCGTCAAGAG		
OL009 F sma-10 AAGTTGCAAGTCTACCAAGCG		
OL010 R sma-10 CCGGTCTTGGAGTTCCTGTG		
OL011 F bcat-1 TCCCGGAGCAAAAGTTCTTCA		
OL012 R bcat-1 TTCTGGACGGAACATGCGAA		
OL015 F elo-1 ACACGAAACGATTTGTGGCTA		
OL016 R elo-1 AGGATTGAAGCCTGAATAGTAACAT		
OL017 F ech-8 GGCTCAGTGGTCTCTTCCAAAT		
OL018 R ech-8 GCGATTGCAATTCCTCTTCCC	All oligos were designed in house	Does not apply to any oligos in this list/ NA
OL009 F sma-10 AAGTTGCAAGTCTACCAAGCG		
OL019 F gba-4 GGATTTGGAGCTGCATTCACTG		

**Software and algorithms**		

RStudio Version 1.2.5033	RStudio Team (2019)	https://www.rstudio.com/
R language Version 3.6.3	R Core Team (2020)	https://cran.r-project.org/
Prism	GraphPad	https://www.graphpad.com/scientific-software/prism/
Cytoscape version 3.8.2	Shannon et al. (2003)	https://cytoscape.org
Python version 3.8	Python Software Foundation	https://www.python.org
Inferelator 2.0	Arrieta-Ortiz et al. (2015)	https://github.com/flatironinstitute/inferelator/blob/release/README.md
MERLIN-P	Siahpirani and Roy (2016)	https://github.com/Roy-lab/merlin-p
Time-lagged LASSO	Nguyen and Braun (2018)	https://github.com/pn51/laggedOrderedLassoNetwork
SeqMonk	Andrews (2021)	https://www.bioinformatics.babraham.ac.uk/projects/seqmonk/
Stochastic Block Model	Peixoto (2014)	https://graph-tool.skewed.de/

### Resource availability

#### Lead contact

Further information and requests for resources and reagents should be directed to and will be fulfilled by the lead contact Marta Sales Pardo (marta.sales@urv.cat).

#### Materials availability

• *C. elegans* strains generated in this study are available upon request.

### Experimental model and subject details

Most assays performed in this study used sterile *glp-1(e2144)ts* or *fem-3(q20)ts C.elegan*s that were maintained at 16°C on NGM with OP50 *E. coli*. To induce sterility, L1 synchronised larvae were added to NGM plates containing HTT115 *E coli* at 25°C.

#### Worm maintenance and synchronization to obtain time-series RNA-seq datasets

Worms were maintained at 16°C on NGM with OP50 *E. coli*. Synchronised experimental populations were prepared by washing gravid adults and eggs from plates and bleaching in a freshly prepared solution of 1% sodium hypochlorite and 1 M potassium hydroxide. Eggs were allowed to hatch overnight at room temperature in M9 solution (22 mM KH_2_PO_4_; 42 mM Na_2_HPO_4_; 86 mM NaCl; 1 mM MgSo4) to ensure all animals arrested at the L1 stage. For time series transcriptomics, *glp-1(e2144)ts* was crossed with an *mlt-10*p::GFP molting reporter (kind gift from the Frand lab) to identify the exact molting times and further aid synchronisation. Conditional sterility was obtained by growing L1 larvae to adulthood at restrictive temperature (25°C).

#### Worm maintenance and synchronization of worms exposed to environmental and metabolic perturbations

The following treatments were used prior to obtaining transcriptomics using MOC82 (Table S18). For oxidative stress the strain was treated at day 1 of adulthood (41 hours) and day 2 (65 hours) for 1 hour by placing animals in 9 cm plates containing 200mM of Paraquat (N, N′-dimethyl-4,4′-bipyridinium dichloride, Sigma). To induce endoplasmic reticulum stress MOC82 worms were placed in 9cm plates containing 5mM of DTT/ Dithiothreitol(Sigma) for 1hour at both day 1 (41 hours) and day 2 (65 hours) of adulthood. To induce heat stress, MOC82 day 1 (41 hours) and day 2 (65 hours) of adulthood were subject to heat stress by placing sealed 9 cm plates in a 34°C water bath for 30 minutes. For the following interventions, MOC82 animals were treated at day 1(45 hrs), day 2(69 hours), day 3(93 hours) and day 4(117 hours). Dafachronic acid (DA) treatment: 1mM Δ7-Dafachronic acid (3-oxo-cholest-4-en-26-oic acid/Cayman #14101), (as described in Deline et al., 2013. In the case of DA, the stock was prepared in ethanol and kept at −20°C. Prior to use, this was diluted 1:100 in PBS and 500μl dropped evenly over the lawn of HT115 plates prepared as described. Animals were treated with 50uM of Arachidonic acid (C20:4n6, Cayman), using 9cm NGM plates containing AA and NP40 0.01%. Both AA and DA plates also contained 10 mg/ml Nystatin to prevent fungal contamination. Controls were grown in NGM plates containing 10mg/ml Nystatin and no acid treatment and NGM devoid of both, collected 45 and 69 hours after feeding and additional control was collected.

#### Worm maintenance and synchronization for RNAi-screening

For L1 screen, *glp-1(e2144), rrf-3(pk1426)* or *fem-3(q20)ts* animals were bleach-synchronized as L1s and 25 to 50 synchronised L1 worms were grown in a 96- or 24-well plate at 25°C .

For L4 screen, *glp-1(e2144), rrf-3(pk1426)*were bleach-synchronized as L1 were added to OP50 seeded plates and incubated at 25°C until the L4 stage. L4 worms were then gently washed 3 times with M9 in order to remove OP50. Finally, 25 to 50 animals were added onto each well of the 24-well RNAi plates and incubated at 25°C until scoring time.

#### Worm maintenance and synchronization for epistasis analysis

*glp-1(e2141)* and *daf-16(mu86); glp-1(e2141)* animals were grown at 25°C and fed from L1 with HTT115 bacteria.

#### Worm maintenance and synchronization for high throughput nanofluidic qPCR

Worms for this experiment were grown from L1 or from L4 onto RNAi plates, specified in figure legends until day 2 of adulthood, when they were harvested to prepare cDNA.

#### Worm maintenance and synchronization for microscopy screening

*glp-1(e2144); dhs-3*p::dhs-3::GFP , *glp-1(e2144); sod-3*p::GFP and *glp-1(e2144); lgg-1*p:LGG:GFP worms were grown on 12-well RNAi plates from L1 to day 4 of adulthood at 25°C.

### Method details

#### RNA-sequencing

Eggs were collected by bleaching and L1 larvae were hatched in the absence of food. Synchronisation was obtained by adding L1 larvae to plates with HTT115 *E coli* strain containing the empty vector plasmid L4440 on standard NGM plates containing 50 μg/ml Carbenicillin and 1 mM IPTG until harvesting and grown at 25°C. The time of adulthood was estimated as time after feeding L1 larvae (as described in Figure S1) and monitored visually using the *mlt-10*p:GFP molting reporter. Samples of at least 1,000 worms were prepared as described in Hastings et al., 2019. The main dataset consists of 113 of *ad libitum*, environmental and chemical perturbation transcriptomics—57 are steady-state time series at 19 sequential timepoints and 56 are perturbation time series at two or four timepoints. For PCR library enrichment, 13 cycles of amplification were performed. Libraries were sequenced on an Illumina HiSeq 2,500 system by the Babraham Sequencing Facility.

#### mRNA-seq quantitation and normalisation

The libraries were trimmed with trim galore version 0.4.4 The libraries were trimmed with trim galore v0.4.4 using default parameters (Krueger, 2021). Trimmed data were mapped to the *C. elegans* WBCel235 genome assembly using HISAT2 v2.1.0 (Kim et al., 2015) guided by splice junctions taken from Ensembl version 75. Mapped positions with MAPQ < 20 and all non-primary alignments were discarded. MAPQ score stands for the MAPping Quality score. A MAPQ score of 20 represents that the probability of correctly mapping a random read is 99%. Sequence processing was carried out by the Babraham Bioinformatics. Raw counts were then generated using SeqMonk (Andrews, 2021). Due to overlapping exons found in *C. elegans* genome annotation, only sequencing reads that were precisely matched to the genome assembly were quantified.

The raw counts were then normalised using the mean expression of genes whose expression levels were identified as consistent across all libraries. Table S19 lists the stably-expressed genes used for the normalisation. The analysis was performed in R.

#### RNA interference

RNAi by feeding was performed as described (Kamath et al., 2000). RNAi clones were taken from the Ahringer's RNAi library (Source Bioscience). Briefly, the clones were inoculated overnight at 37°C in LB plus 50 μg/ml ampicillin and were then seeded onto fresh RNAi plates composed by NGM plus 25 μg/ml carbenicillin and 1 mM IPTG. Worms were exposed to RNAi bacteria from L1 or from L4 (specified for each assay).

#### High throughput nanofluidic qPCR

cDNA from day 2 adult animals was prepared as described in (Chauve et al., 2020). Briefly, 10 worms were lysed and RT was directly performed using the Power SYBR Green Cells-to-Ct kit (Invitrogen, 4402955). The cDNA was then analysed using nanofluidic technology developed by Fluidigm using the standard protocol adapted from Fluidigms’User Guide and by (Chauve et al., 2020). 1.25μL of the cDNA solution was used for pre-amplification. Then, a 96.96 Dynamic Array IFC was used to measure the expression level of 96 genes in 96 different biological conditions. Primers used in RT-qPCR were designed to span exon-exon junctions when possible or to have an intron between them. Primers are listed in Table S18. Melting curves were examined to ensure primer specificity. Results were analysed using the standard ΔΔCT method. Expression levels of target genes were normalised using *cdc-42*, *ire-1* and *pmp-3* as reference genes. At least six biological replicates were analysed per condition. The coefficient of correlation between the knockdown efficiency and any tested genes was calculated using Pearson’s correlation coefficient.

#### Two-step *glp-1(ts)* lifespan screening

##### *Glp-1(ts)* lifespan screen 1

The initial screen included 1120 RNAi clones divided into 96-well plates. Each 96-well plate was tested together and divided further into four 24-well plates. Each clone was initially grown in liquid LB in 24-well plates. 25μL of each culture was seeded in duplicate on 24-well plates. A plate was seeded in duplicate with the empty vector (L4440) as a negative control and vectors expressing *daf-16* and *age-1* as positive controls. Each clone was seeded in 2 independent wells of 24-well plates and 2 control plates were used with 24 wells seeded with L4440. 25 to 50 synchronised L1 worms were grown in each well. Contaminated plates, starved plates, or plates with outwardly defective or arrested animals were discarded (104 conditions).

##### *Glp-1(ts)* lifespan screen 2

93 candidates were selected based on novelty as explained in the main text. The experiment was set-up as described above and the percentage of survival was assayed every 2 to 3 days (at days: 3, 5, 7, 10, 12, 14, 16, 17, 19, 21, 24 and 26) by scoring animals based on movement. Three biological replicates were conducted and each of them included two technical replicates. To ensure the identity of the genes that were knocked down, each one of the clones that significantly change the lifespan was sequenced using M13 forward primer. Day 19 normalised percentage of survival was calculated using the same procedure. For this screen, we used day 19 survival rate as we found that it is closer to the mean survival.

#### *Fem-3(ts)* lifespan screen

To evaluate the effect of RNAi clones in normal lived animals we used *fem-3(q20)ts.*The experiment was set-up at 25°C as described in the two-step *glp-1(ts)* lifespan screening, and the percentage of survival was assayed at day 13 of adulthood, which is the time point when animals grown in bacteria expressing empty vectors reached roughly 50% survival. *fem-3(q20)ts* survival at day 13 was compared with *glp-1(ts)* survival at day 19, at which time both strains have reached 50% survival.

#### L4 *glp-1(ts*) screen

*glp-1(e2144), rrf-3(pk1426)* animals were grown as follows: bleach-synchronized L1s were plated onto OP50 seeded plates and incubated at 25°C until L4 stage. L4 worms were then gently washed 3 times with M9 in order to remove OP50. Finally, 25 to 50 animals were seeded onto each well of the 24-well RNAi plates with a Pasteur pipette.

#### Lifespan epistasis experiments

*glp-1(e2141)* and *daf-16(mu86); glp-1(e2141)* animals were grown at 25°C and fed from L1 with RNAi bacteria containing either empty vector (EV) or double stranded RNAi for *sup-37* or *sdc-3*.

#### Microscopy

*glp-1(e2144); dhs-3*p::dhs-3::GFP , *glp-1(e2144); sod-3*p::GFP and *glp-1(e2144); lgg-1*p:LGG:GFP worms were grown on 12-well RNAi plates from L1 to day 4 of adulthood at 25°C. On the day of imaging, 300μL of M9 with 100μM levamisole were used to transfer worms from the 12-wells RNAi plate to black flat bottom 96-wells plate (CellCarrierTM-96, Black, clear bottom, TC Treated). Worms were washed 2 times with 300μL of M9 containing 3mM of Levamisole (Sigma). The image of an entire well was acquired automatically using a Nikon Ti-E microscope equipped with an Elements (using JOBS module) system and a Hamamatsu Flash 4.0 v2 camera. The objective 10X was used. 5% of the laser was used with 200ms exposure. These settings were identical for all experiments. Using FIJI image processing software, a mask was applied to remove irrelevant information from outside the well. Then raw images were thresholded (using "Huang dark” algorithm) to outline the worm body, the green channel was used for the *dhs-3*p::dhs-3::GFP fluorescent reporter images and the bright-field channel was used for *sod-3p::GFP* fluorescent reporter images. This was used as a mask to measure the mean intensity per well and the background was then subtracted. An in-house script was used to automatically analyse the images (available upon request). For each fluorescence reporter, 3 to 4 biological replicates were analysed with 10 to 80 worms per condition. For the statistical analysis, the fluorescence of the worms grown on bacteria with the empty vector L4440 was used as a baseline.

#### Construction of a mechanistic TF-gene (physical) and gene-gene (functional) interaction database

We built a WT functional interaction database that includes two types of interactions: TF-gene interactions and gene-gene interactions. We identified TF-gene interactions from ChIP-seq data, motifs, eY1H assay, and transcription start sites (TSS) from Ensembl version 75 (Aken et al., 2017). We filtered TF-binding sites using ATAC-seq open regions. To identify TF-binding sites that are open from ChIP-seq and motifs, we first used *bedtools intersect* and *bedtools merge* commands (version 2.29.0, Quinlan and Hall, 2010) and only kept TF-binding sites that overlapped with an open ATAC-seq region by at least one base pair. We then inferred interactions from TFs to target genes by aligning TSS to the TF-binding sites from ChIP-seq and motifs, using the *bedtools* window command with 1000 bp window size. For eY1H data which already are interactions between TF and genes, we only included an interaction if the TSS site of the target gene overlaps with an open ATAC-seq region by at least one base pair according to the *bedtools intersect* output (version 2.29.0, Quinlan and Hall, 2010).

We used the following sources of TF-gene interactions: 1) 115 L4 or young-adult ChIP-seq datasets from ModERN (Kudron et al., 2018), 2) two young-adult ChIP datasets (GSE28350, GSE81521) (Hochbaum et al., 2011; Li et al., 2016), 3) 202 unique TF DNA recognition motifs using “direct evidence” option from CiS-BP motif database (Weirauch et al., 2014), obtained through RTFBSDB R package (Wang et al., 2016), and 4) 13,501 TF-gene interactions from eY1H assay (Fuxman Bass et al., 2016). Regulatory sequences were obtained using biomaRt R package (accessed on 31^st^ Oct 2017) (Durinck et al., 2009). This study used WBcel235/ce11 version of the *C. elegans* genome, and WormBaseWS260 genome annotations.

For gene-gene interactions, we based our curation on the WormExp v1.0 database (Yang et al., 2016) which has compiled nearly all *C. elegans* published expression data over the past decade (last updated on 27/07/2017) (Yang et al., 2016). Out of the 361 studies, 298 studies were in ’Mutants’, ’DAF/Insulin/food’, ’Development/Dauer/Aging’, and ’Others’ categories, and were included in the curation. All 298 publications were carefully read to filter for studies fulfilling the following criteria: 1) a minimum of three citations, 2) the background was either N2 (i.e., which is wild-type), or mutants with N2 background, and 3) the age of the worms at the time of harvest is L4 to day 4 of adulthood. We finally included 98 studies in the database covering 126 different regulator genes. Table S1 lists the references and Table S3 lists the final database of TF-gene and gene-gene interactions (WT-GS).

#### Definition of empirical modules

We curated nine eight *glp-1(ts)* studies, which included a total of 52 different RNA sequencing knockdown datasets that we normalised using the DESeq2 pipeline (Love et al., 2014). Details of the quantitation and GEO accession numbers are listed in Table S9. We divided each *glp-1(ts)* dataset into five empirical modules according to the levels of observed gene expression changes. These empirical modules contain genes that show the same response to a genetic perturbation and therefore enable us to assess whether structural modules that group genes according to the role they play within the GRN (see below) follow grouping patterns similar to empirical modules.

• Empirical module 1- Differentially upregulated at greater than two-fold change (p-adjusted< 0.05)
• Empirical module 2- Differentially upregulated between 0 to two-fold change (p-adjusted< 0.05)
• Empirical module 3- Differentially downregulated at greater than two-fold change (p-adjusted< 0.05)
• Empirical module 4- Differentially downregulated between 0 to two-fold change (p-adjusted< 0.05)
• Empirical module 5- Not differentially expressed

### Quantification and statistical analysis

#### Adjusted p-values for correlation calculation in RNAi knockdown experiments

In Tables S7 and S16 we show the results from experiments in which we knock down (KD) a regulator gene using RNAi and look at the effect of this KD on the expression of a target gene in at least six replicate experiments. To assess the effect of a KD in a target gene, we compute the Pearson Correlation Coefficient (PCC) between ΔΔCT values in the N replicates between KD regulator gene and target gene using the “cor” function with the default method, i.e. pearson correlation, in the base R library in RStudio Version 1.2.5033. Because the number of points is small (N=6, 7; we excluded KD with lower than 6 effective KD from the analyses) and we look at correlations of many targets for a given KD regulator, correlation values are highly dependent and we cannot use standard p-values and multiple testing corrections. Instead, we use a resampling method to obtain a distribution of extreme correlation values for each KD and obtain the p-value of the correlation values between a target gene and a given KD regulator from this distribution. Specifically, we obtain a distribution of 1,000 extreme correlation values (absolute values) for each KD regulator. To obtain each one of these extreme correlation values, we shuffle the vector of ΔΔCT values of each target using and compute PCCs between expression values of each target gene with the KD regulator; we then keep the largest PCC (in absolute value). After the 1,000 iterations we obtain a distribution of extreme (largest in absolute value) PCCs conditioned on the expression vector of the KD and target genes and we can compute the p-values of each observed PCC of that KD regulator with any target from this distribution. Because to obtain this distribution we consider all of the expression vectors for all of the targets, we do not need further adjustment of the p-values. We used python3 to implement the algorithm and scipy.stats.pearsonr to obtain PCCs.

#### Statistics used to analyse significance in the two-step *glp-1(ts)* lifespan screening

##### *Glp-1(ts)* lifespan screen 1

Percentages of survival were obtained at day 16 of adulthood (D16) in worms grown at 25°C. At that time, *glp-1(e2144)ts;rrf-3(pk1426)* animals grown in bacteria containing the empty vector had reached about 50% of survival.

Percentage of survival:$\frac{Number\;of\;dead\;worms\;}{Average\;number\;of\;dead\;and\;alive\;worms\;at\;each\;timepoint}$∗ 100

For each condition, we performed two technical replicates. Because each 96-well plate was not tested at the same time, we used control L4440 plates that were performed at the same time (same round of experiments) for normalisation. For each round of experiment (96 clones), 24-well plates of control were analysed and the average of percentage of survival was used. To normalise, we divided the % of survival of the test by the average % of survival of the controls in the same round of experiments. We then calculated the mean of the ratios of the two technical replicates. To obtain the normalised percentage of survival we added 50 to this ratio (centering the result at 50 because D16 was considered to be the mean survival of the controls).

##### *Glp-1(ts)* lifespan screen 2

Day 19 normalised percentage of survival was calculated using the same procedure. For this screen, we used day 19 survival rate as we found that it is closer to the mean survival. To obtain survival curves, we combined the number of alive worms of the two technical replicates for each time point and similarly for the number of dead worms. The percentages of survival were then calculated for each time point and the mean sample size (the sample size may vary due to experimentation error) was calculated. The values were used to construct survival curves using the survival R package. Each survival curve was compared to the L4440 survival curve using the log-rank test. P-values were obtained for each biological replicate and combined using Fisher’s method. Bonferroni multiple correction was then applied and the conditions with a p-value lower than 0.05 were considered significantly changing the lifespan.

#### Statistics used to analyse lifespan epistasis experiments

The data was analysed with a log-rank (mantel Cox) test to measure statistical significance. Three replicates have been performed and a Fisher exact test was used on the three log-rank p values to establish overall significance. The software Prism was used to compute these values.

#### Statistics used to analyse microscopy data

Statistics were based on either one-way Anova or a mixed model (depending on the number of replicates) followed by FDR. A two-stage step-up method of Benjamin, Krieger and Yekutieli was used to calculate the statistical relevance of each condition compared to L4440. The software Prism was used to compute these values.

#### ATAC-seq data reconciliation

Whole-worm young adult ATAC-seq data (Daugherty et al., 2017) were retrieved from from the Gene Expression Omnibus database (https://www.ncbi.nlm.nih.gov/geo/) (Barret et al., 2013) under accession numbers GSM2385311 (4 datasets, young adult replicates and the input control). We re-quantitated the samples using a 200 bp running window with a step size of 200. We only kept probes with lower than 5000 read counts and applied a post-quantitation normalisation using a matched distribution percentile normalisation ("multiply to the 95.0 percentile" option in Seqmonk).

We applied *hiddenDomains* R package (Starmer and Magnuson, 2016) to each replicate separately, as we deemed the input sample not viable. We considered regions with the minimum of 0.7 enriched probability as open regions. Using the *bedtools intersect* and *bedtools merge* (version 2.29.0, Quinlan and Hall, 2010)*,* open regions which exist in at least two out of the three replicates were included as final open regions.

#### Genome-wide *C. elegans* gene regulatory network inference

##### Gene input selection

To select genes whose gene-expression time series would be fed to network inference algorithms, we applied a threshold of a minimum of log2-difference between the highest and the lowest values across all time conditions. Out of 20,191 protein coding genes, 12,884 genes were above that threshold and thus, RNA-seq data for these genes was used as input for the inference algorithms.

##### Network inference algorithm selection

We used Inferelator (Arrieta-Ortiz et al., 2015), MERLIN-P (Siahpirani and Roy, 2016) and Time-lagged Ordered Lasso (Nguyen and Braun, 2018) (TOL) inference algorithms to infer networks from time series RNA-Seq. The three methods use three types of input data: a time series of gene expression data of a given length, a list of input regulators (genes which can regulate the expression of other genes in the dataset; Table S17) and known regulatory interactions between genes (priors, Table S2).

##### Input data

For Inferelator and MERLIN-P, we labelled replicates of the time series expression separately and sequentially, and included both steady-state and perturbation libraries. For TOL, we only used steady-state libraries so that the input for each condition is the average of the values across replicates. We considered five different prior sets as illustrated in Figure 1 showing data types included in each corresponding set. We also considered three regulator sets (Table S17): **a.** 632 ageing-related genes from GenAge database; **b.** 721 transcription factors (Kudron et al., 2018); and **c.** 1,442 high variability genes identified by maSigPro R package (Nueda et al., 2014). For Inferelator and MERLIN-P, we used regulator types **a** and **b** as input and 12,884 as targets. For TOL, we used genes in sets **a, b,** and **c** as regulators and targets (2,553 genes) to fit the tool’s requirement. Time-series lengths are either a full time series, L4 until day 10, or a shorter time series, L4 until day 3 of adulthood. We used every possible combination of the three input variables in our inference (Table S4).

###### Implementation

Inferelator: the number of bootstraps was set to 100. Prior weight was set to 1.2 (Arrieta-Ortiz et al., 2015). Remaining parameters were set to default values. MERLIN-P: All parameters were set to default. Weights of priors were assigned according to their data type (ChIP-seq edge weight = 0.8; eY1H, motif edge weight = 0.6). TOL: TOL algorithm was modified to output the sum of all βs for all time points for every gene. l_max_ was set to 5. λ_prior_ was 0.5 and λ_non−prior_ was 1.

##### Consensus network building

We obtained 50 different directed, binary GRNs for the different combinations of input data, prior and NI tool (Table S5). We then compared these networks by measuring their edge overlap, i.e., the fraction of overlapping edges between pairs of networks. Using this as a similarity metric between networks, we used hierarchical clustering (pheatmap R package) to identify distinct groups of networks that have large edge overlaps. (See Table S5 for the inferred networks in each group labelled by NI tool and input variables) p. For each group we build a consensus network as the union of all networks within that group, i.e. by keeping unique edges of all networks within the group.

#### Significance calculation of enrichment of sets of genes by resampling

We use resampling to compute: 1) the enrichment of GenAge genes and orthologous genes that act as regulators in our inferred GRNs with respect to the input regulator set; and 2) the enrichment of GenAge genes in genes that have a >20% impact in lifespan with respect to genes than were tested in lifespan screen 1.

The resampling methodology works as follows: initially we have a set of Ng genes (for instance genes tested in lifespan screen 1), in which Mg genes are labeled (for instance, Mg genes appear in GenAge Database). Then we have a set of Nt (<Ng) genes (in the example, genes with >20% impact in lifespan with respect to the control) in which Mt (<Mg) genes are labeled (in the example, they appear in GenAge). We want to know what is the probability of finding Mt labeled genes in a subgroup of Nt genes from a group Ng genes where Mg genes are labeled. To do that we generate 10,000 random samples of Nt genes from the initial set of Ng genes and compute for each sample the number of labeled genes Nr. From this distribution of {Nr} we can obtain the p-value of the observed fraction of labeled genes.

#### Performance metrics for validations against the gold standard

Our Gold Standard dataset covers a very small fraction of the possible regulators (<5%) in the network. Usual performance metrics are hard to interpret since the fraction of data that is testable is a very small amount of the potential number of interactions that can be inferred. Because of this, we resorted to performance metrics that are more suitable to measure whether the observed signal differs from random or not. Specifically, we use the precision fold enrichment introduced in (Roy et al., 2013) and the area under the precision fold enrichment curve.

##### Precision fold enrichment

It measures the precision (that is the fraction of predicted positive interactions that are positive in the GS) versus the probability that an inferred edge is in the GS set:

$$\text{Precision}\;\text{fold}\;\text{enrichment}\;\left(\text{PFE}\right)=\frac{\frac{p}{n}}{\frac{k}{K}}$$

where p is the number of correct predictions, n is the total number of edges inferred, k is total number of GS edges, and K is the total number of edges that is possible to infer (#regulators x # targets). A PFE >1 indicates that the precision is larger than what we would expect at random. It is important to note that PFE does not have an upper bound. The upper bound depends in general on the size of the GS and that of the inferred network. However, it is useful to compare different GRNs obtained for the same set of genes and validated against the same GS as is our case.

##### Area under precision fold enrichment curve (AUFE)

It is the area under the curve in which the x axis indicates a PFE value and the y axis represents the fraction of regulators with a PFE value equal or larger than that in the x axis. Note that while PFE is not bounded in theory, it does have a maximum (PFE_max)_ for each network and GS validation set so that it can be computed.

AUFE measures how evenly distributed PFEs are across regulators in the GS validation set (measurable regulators). For networks with similar PFE_max_ values, the larger AUFE the better. To illustrate this idea, consider two networks with the same PFE_max_. In one network all measurable regulators, *r,* have PFE_r_ equal to PFE_max_, so that AUFE is equal to PFE_max_. In the second case, we have the same regulators so that all of them have PFE_r_ =0 except for one that has PFE_r_ = PFE_max_, so that AUFE=PFE_max_/2, which is smaller than that of the first (and preferred) situation.

#### Precision for empirical validation experiments and random expectation

We tested the effect of knocking down (KD) 10 different regulator genes on 190 targets (Table S7). We looked at the change in expression of regulators and targets in at least 6 replicates and obtained the Pearson correlation coefficient for each regulator-target pair.

To validate the network, we used a PCC cut-off (PCC≥ |0.75|). For a regulator-target pair that has a PCC ≥ |0.75|, we consider the interaction a positive validated edge if the edge corresponding to that regulator-target pair appears in at least one of our consensus networks, counting towards precision and accuracy. If a regulator-target pair has a PCC< |0.75|, we take that as a negative empirical interaction; if that edge does not appear in any of our consensus networks, we count that edge as a negative confirmed interaction which counts toward the accuracy.

Because the precision and accuracy we report are cut-off dependent, we also measure the AUPR, that is the area defined by the curve of (precision, recall) values we obtain by sweeping over all PCC cut-off values.

To obtain a random expectation, we make two types of assessments:

1. Naive expected precision. Assuming that the number of edges in the overall network is fixed and given by our procedure of filtering for edges with low weight in the NI process -which is 313,562 for the target network-, if we were to distribute the observed edges among all the possible regulator (2,795) and target (12,884) pairs, then the expected precision would be equal to the density of that network: 313562/(2,795x12,884) =0.0087.
2. Expected precision/accuracy from randomising the networks. We made 100 randomisations of each one of the three networks by using a double edge swap approach in which we select two edges at random (u→ v) and (s → t) and we swap the targets between edges to obtain a rewired set (u→ t) and (s → v). This procedure preserves the degree of both regulators and targets and allows to assess whether any observed network feature can be explained solely by the degree distribution of its nodes or not. Using the same procedure described above we calculated precision and accuracy for each randomisation. We then obtained from this ensemble the expected average precision and accuracy as well as their standard deviations to obtain Z-scores.

#### Bayesian model selection with stochastic block models (SBM) and definition of structural modules

Stochastic block models are simple generative models that assume that there are underlying groups of nodes in the network and that the probability that there is an edge running between nodes (i,j) only depends on the group memberships of i and j. As generative models, SBMs are amenable to Bayesian inference, and therefore to model selection techniques that allow us to find the best division of the nodes into groups. Nodes in the same group have statistically similar connection patterns and are thus interpreted to play a similar role in the network. Note that there is no a priori selection neither of the number of groups nor of the interactions between the groups. SBMs have been shown in the literature to be appropriate models for real network topology being successful at both error prediction (Guimerà and Sales-Pardo, 2009) and community detection (Peixoto, 2014).

We use a minimum description length approach (MDL), which is equivalent to maximizing the posterior, to find the best division of nodes into groups (https://graph-tool.skewed.de/; Peixoto, 2014). Specifically, we use the MDL approach to identify the best SBM variant (non degree-corrected and degree-corrected with and without hierarchical priors for the groups). Because the minimisation process is heuristic, we ran the algorithm 1,000 times to identify the best model (with minimum description length, Σ) and therefore best division of nodes into structural modules. We find that the best model is a degree-corrected SBM with hierarchical priors. We therefore obtain a hierarchical tree of network divisions into structural modules. Note that there is no a priori selection neither of the number of groups nor of the interactions between the groups. The inference methodology finds the division into groups that best describes the observed topology.

##### Input-core-output structure

We represent structural modules, *m*, at the second most coarse-grained level in the hierarchy-- level 1 (Figure S2D). We select this level because it summarises the networks and it is the first level that is significantly correlated with empirical modules for the two smallest GRNs we select (max AUFE and max PFE). In Figures 3, S3, andS4, connections between structural modules have a weight equal to the number of connections between genes in the two modules. We only represent connections with a weight > 260 to represent the main structure of the network of structural modules. All selected consensus GRNs have an input-core-output structure. In networks with this kind of topology it is possible to define three layers with different topological properties. The *input* layer has genes that are either connected to genes in other layers or with genes within the same layer. The *core* layer has genes that are connected either to genes in the *output* layer or to genes in the *core* layer. Genes in the *output* layer only connect to genes in that same layer. This type of networks thus has a clear direction of ‘information’ or regulatory flow from the input layer to the output layer.

To better characterize the input-core-output structure, the tables below show the aggregate connections between the input, core and output layers.

**Table:** 

max AUFE	TO/FROM	Input	core	output
	input	18417	571	0
	core	249216	8428	0
	output	735	34526	1446
**max PFE**	input	41287	761	0
	core	226597	1438	0
	output	164	8019	112
**middle PFE/AUFE**	input	15026	474	0
	core	247117	7681	16
	output	209	9218	126

Note that the majority of connections go in the direction input→ core → output (89% on average) and that most of the remaining connections are within layer connections indicating complex regulatory interactions especially in the input layer; only 0.2% of the connections on average fall out of the main input-core-output connectivity pattern.

#### Recovery quantification of empirical gene modules by structural gene modules

##### Topological approach using the Jaccard index

To measure the overlap between empirical modules *G ={G1,..G5}* and structural SBM modules *m,* we compute the Jaccard index = $\frac{m\cap G}{m\cup G}$ for each one of the empirical modules against each one of the structural modules in the hierarchy level 1. We then generated an expected distribution of random Jaccard indices by reshuffling cluster and group memberships 1,000 times. From this distribution we computed the robust Z-scores of the observed Jaccard indices.

The robust Z-score is a standardised score used when the distribution of values deviates from normality:

$$\text{Robust}\;\text{Z}-\;\text{score}=\frac{x\;-\;median}{1.4826*MAD}$$

where MAD is the median of the absolute deviation from the median:

MAD =median(|xi−medianx|)

For each empirical group, we selected the structural group with the maximum robust Z-score. If the selected robust Z-score is >1.96 (p-value of 0.05 based on a right-tailed Fisher’s exact test), the corresponding empirical cluster is considered to be structurally "recovered" in the network (Figure 2C).

##### Information-theoretic approach

The mutual information (Cover and Thomas, 2006) allows to compare two partitions of items into groups. It measures the amount of ‘bits of information’ shared between the two partitions. We used the python 3 scikit-learn implementation to compute the mutual information between the SBM partition of nodes into groups at the different hierarchical levels and the empirical clusters. To assess the difference between the measured mutual information and that expected at random, we generate 10,000 randomised group assignments keeping the sizes of empirical and SBM clusters fixed. From this distribution we obtain the Z-score of the observed mutual information (Figure S2D). We also show the adjusted Z-score considering that for each network we perform 36 different tests (9 sets of empirical clusters x 4 levels of SBM partitions).

### Additional resources

#### Gene interaction network interactive webpage

The webpage (https://s-andrews.github.io/wormgrn/qpcr/) plots a subnetwork of a chosen regulator based on qRTPCR data (Tables S7 and S16). The edges can be filtered based on PCC and p-value of edges (see Quantification and statistical analysis). Colour intensity of edges varies according to PCC values.

## Data Availability

• This paper does not report original code. We specify the tools we use in the Quantification and Statistical Analysis Section. • Any additional information required to reanalyze the data reported in this paper is available from the lead contact upon request. • RNA-seq and microscopy data have been deposited at Zenodo and are publicly available as of the date of publication. Accession numbers are listed in the key resources table. • The input data and gold standard datasets were generated with publicly available data. The main dataset is listed in the key resource table. Other datasets are listed in Table S1 and available on zenodo.

## Supplemental information

Document S1. Figures S1–S8 and Tables S4, S5, S9, S11, S13, and S20

Table S1. Datasets used as prior information, related to Figures1 and S1 and Table 1

Table S2. Physical priors from mechanistic inference, related to Figures 1 and S1 and Table 1

Table S3. List of WT gold standards, related to Figures 1, S3, and S4

Table S6. Final three selected networks, related to Figures 1, S3, and S4

Table S7. qRTPCR results and correlations used to calculate network accuracy, related to Figure 2

Table S8. SBM division into hierarchy of modules of each network, related to Figure 3

Table S10. First screen on 1,120 network regulators on *glp-1(e2144)ts;rrf-3(pk1426)*-RNAi from L1 to day-16 adulthood, related to Figure 4

Table S12. Second screen for 93 top genes on *glp-1(e2144)ts;rrf-3(pk1426)* detailed lifespan assays, worms treated from L1, related to Figure 4

Table S14. Day 13 survival of *fem-3(q20)ts* vs day 19 survival of *glp-1(e2144)ts;rrf-3(pk1426)* RNAi from L1, related to Figure 4

Table S15. in vivo fluorescent reporter data of *glp-1(e2144)ts;rrf-3(pk1426)*, related to Figure 5

Table S16. qRTPCR results for the gene interaction network of new aging genes and targets and the weighted, directed gene-interaction network, related to Figure 5

Table S17. List of genes used as an input regulator and its category (GenAge, TF or High variability) and orthologues, related to to Figure 1, Figure S3 and Figure S4.

Table S18. Strain and primers used in the study, related to Star Methods.

Table S19. List of non-variable genes used for RNA-Seq normalisation, related to Star Methods
