# Supplementary material for: Gene regulatory network inference in long-lived C. elegans reveals modular properties that are predictive of novel aging genes
Source: iScience. 2021 Dec 20;25(1):103663. doi: 10.1016/j.isci.2021.103663 (PMC8753122; doi:10.1016/j.isci.2021.103663)
Supplement: Document S1. Figures S1–S8 and Tables S4, S5, S9, S11, S13, and S20 [file mmc1.pdf]

iScience, Volume *25*

Supplemental information

Gene regulatory network inference in long-lived *C. elegans* reveals modular properties that are predictive of novel aging genes

Manusnan Suriyalaksh, Celia Raimondi, Abraham Mains, Anne Segonds-Pichon, Shahzabe Mukhtar, Sharlene Murdoch, Rebeca Aldunate, Felix Krueger, Roger Guimerà, Simon Andrews, Marta Sales-Pardo, and Olivia Casanueva

Supplemental Material:

Figures:

Figure S1 Detail of transcriptomics data and wisdom-of-the-crowds network inference approach and validation.

Figure S2 *In vivo* measurement of the local accuracy of networks and *in silico* validation of network topology.

Figure S3 The global organization of the gene-regulation network reveals an input-core-output structure with ageing modulators at its core(max PFE network).

Figure S4 The global organization of the gene-regulation network reveals an input-core-output structure with ageing modulators at its core (middle PFE/AUFE network).

Figure S5 RNAi screening uncovers novel ageing modulators

Figure S6 The novel ageing genes cause metabolic remodeling.

Figure S7 The new ageing candidates are impacting the expression of sod-3 and dhs-3.

Figure S8 Relationship of fat storage and oxidative stress in *fem-3* animals.

Supplemental Tables:

Table S4 List of 50 inferred networks and their scores, related to Figure 1, Figure S3 and Figure S4.

Table S5 List of the 50 network membership by the nine block consensus networks, related to Figure 1, Figure S3 and Figure S4.

Table S9 Datasets used for glp-gold standards, related to Figures 2, Figure S3 and Figure S4.

Table S11 Genes that are essential for glp longevity according to Wormbase Version: WS278,
related to Figure 3.

Table S13 List of orthologous genes in fly, mouse, and human for the novel ageing genes, related to Table 2.

Table S20 Epistasis lifespan data of *glp-1(e2144)ts*, related to Figure 5.

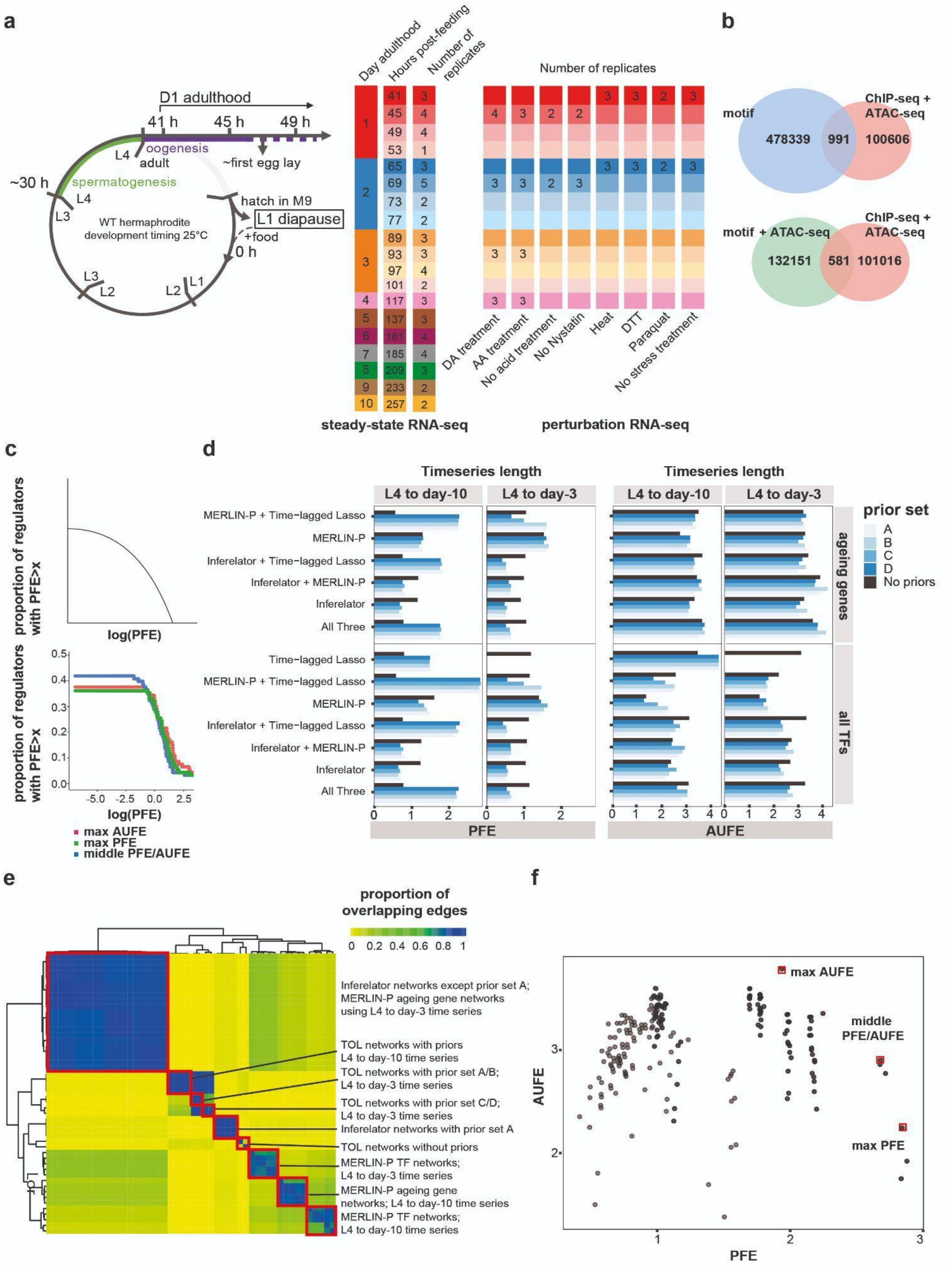

a
D1 adulthood
41 h
45 h
49 h
oogenesis
L4
adult
~first egg lay
~30 h
spermatogenesis
L4
L3
hatch in M9
L1 diapause
WT hermaphrodite development timing 25°C
+food
0 h
L3
L2
L2
L1
Day adulthood
Hours post-feeding
Number of replicates
Number of replicates
steady-state RNA-seq
perturbation RNA-seq
DA treatment
AA treatment
No acid treatment
No Nystatin
Heat
DTT
Paraquat
No stress treatment
b
motif
ChIP-seq + ATAC-seq
478339
991
100606
motif + ATAC-seq
ChIP-seq + ATAC-seq
132151
581
101016
c
proportion of regulators with PFE>x
log(PFE)
max AUFE
max PFE
middle PFE/AUFE
d
Timeseries length
L4 to day-10
L4 to day-3
MERLIN-P + Time-lagged Lasso
MERLIN-P
Inferelator + Time-lagged Lasso
Inferelator + MERLIN-P
Inferelator
All Three
Time-lagged Lasso
prior set
A
B
C
D
No priors
ageing genes
all TFs
PFE
AUFE
e
proportion of overlapping edges
Inferelator networks except prior set A; MERLIN-P ageing gene networks using L4 to day-3 time series
TOL networks with priors L4 to day-10 time series
TOL networks with prior set A/B; L4 to day-3 time series
TOL networks with prior set C/D; L4 to day-3 time series
Inferelator networks with prior set A
TOL networks without priors
MERLIN-P TF networks; L4 to day-3 time series
MERLIN-P ageing gene networks; L4 to day-10 time series
MERLIN-P TF networks; L4 to day-10 time series
f
max AUFE
middle PFE/AUFE
max PFE
AUFE
PFE

Figure S1. Detail of transcriptomics data and wisdom-of-the-crowds network inference approach and validation, Related to Figure 1.

a. Schematic representation of RNA sequencing datasets used for the inference. Left diagram shows the life cycle of *C. elegans* grown at 25°C. The stages are: larval stage 1(L1); larval stage 2 (L2); larval stage 3 (L3); larval stage 4 (L4) and day 1 (D1) of adulthood. The time of harvest (day of adulthood and time post-feeding of arrested L1, shown by colours) as well as the number of replicates used per time point are shown for steady-state samples in the middle panel and for perturbation samples in the right panel. Steady-state samples were *glp-1(e2144)ts* animals grown in standard NGM whilst perturbation samples were *glp-1(e2144)ts* animals treated by incubating them in arachidonic acid (AA), dafachronic acid (DA); Dithiothreitol (DTT), Paraquat and heat shock as described in the methods section.

b. Venn diagrams of the effect of using ATAC-seq data as a filter on motif-derived interactions. Upper diagram shows the number of overlapping interactions of motif-derived interactions and the interactions from modERN ChIP-seq interactions filtered by ATAC-seq open regions. Lower diagram shows the overlap when the motif binding regions were also filtered by the ATAC-seq open regions before inferring interactions from the motif to the nearest genes. The comparison shows the effect of the ATAC-seq filter on quality improvement of motif-derived interactions by using interactions agreed by both ChIP-seq and ATAC-seq data as the standard. The increased overlap ratio is significant (p-value 2.2e-16, Fisher's Exact Test).

c. Schematic illustration of the area under the fold enrichment curve (AUFE). AUFE is the area under the curve formed by the fraction of regulators with precision fold enrichment (PFE) equal or larger than the PFE value on the X-axis, where PFE must be within the interval 0 to 100 . PFE corresponds to the ratio between the proportion of correct predictions and the probability of making a correct prediction at random. In general, PFE captures the accuracy of individual edges whereas AUFE captures the coverage of regulators based on their PFE scores. High AUFE values imply that a large proportion of regulators have high PFE, taking into account individual regulator accuracy

d. Comparison of PFE and AUFE obtained for the 50 inferred networks and their combinations across tools, prior set, input regulator sets, and time-series length. Note that for TOL the input regulator set also includes high-variability genes as described in Methods Sec. III. The left figure shows PFE and the right figure shows AUFE, using functional data from wild-type/N2 as gold standard (WT-GS). Because WT-GS is very sparse, we only considered those networks where at least 10 regulators were represented in the WT-GS dataset. The right panels indicate regulator set and the top panels indicate time series length.

e. Heatmap of the matrix of edge overlaps between pairs of inferred GRNs. Each element represents the number of overlapping edges between the two networks at corresponding row and column names divided by the size of the network at the row. We ordered the matrix using hierarchical clustering which shows nine high-overlap groups of networks along the diagonal. We use the groups to define consensus GRNs (red squares). The labels to the right summarise the inference variables used to generate the networks in each group.

f. **PFE and AUFE comparison of networks from a wisdom-of-the-crowds approach.** We constructed all possible network combinations using the 9 consensus networks obtained in **d** and evaluated them against WT-GS using FE and AUFE scores. We selected three combination GRNs according to their performance (highlighted in the plot): max PFE network, middle PFE/AUFE network, and max AUFE network. The max PFE network scores well in PFE; the middle PFE/AUFE network scores relatively well in both metrics; and the max AUFE network scores relatively high in AUFE. We considered only networks where more than 50 regulators were present within the WT-GS.

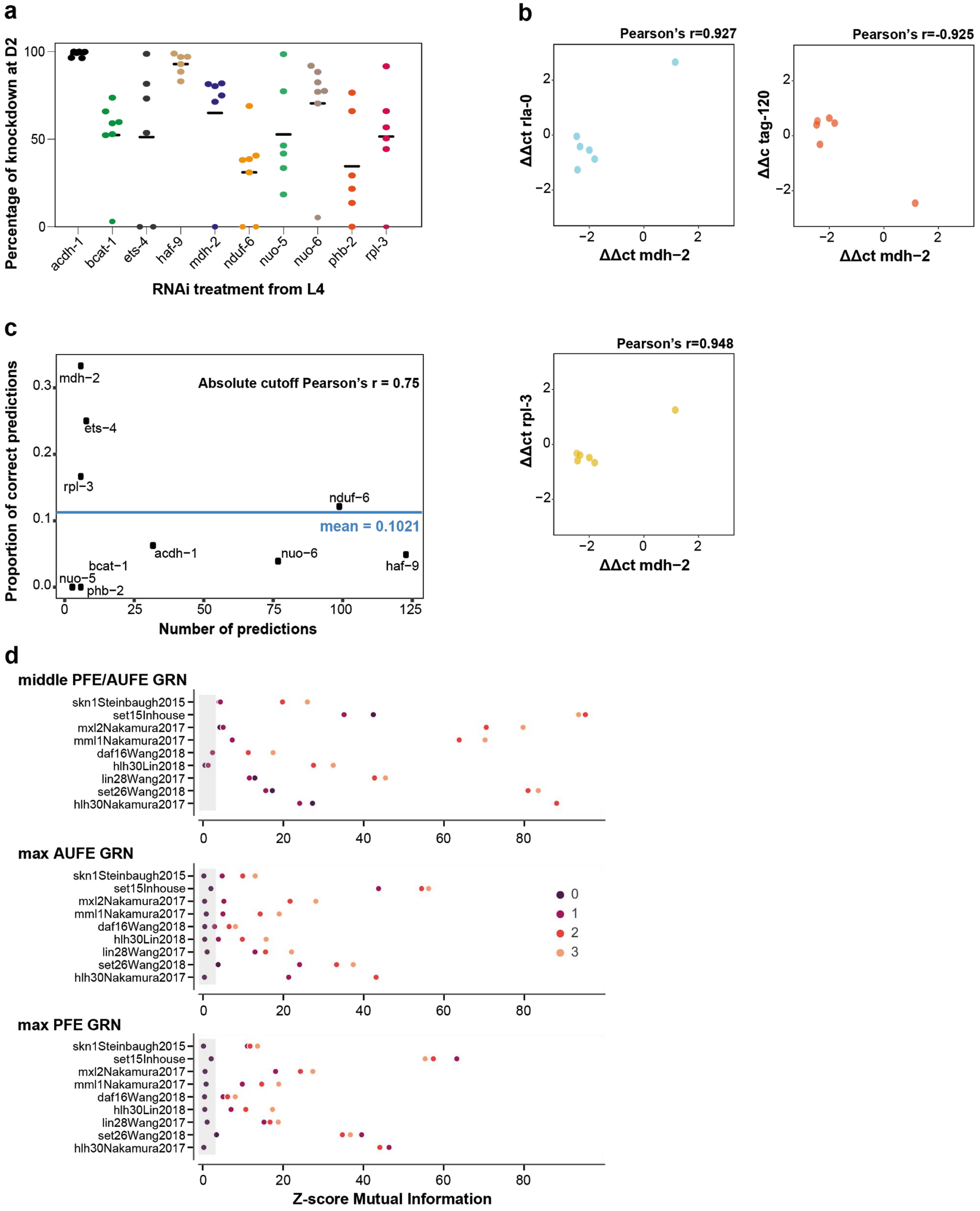
a
Percentage of knockdown at D2
100
50
0
acdh-1
bcat-1
ets-4
haf-9
mdh-2
nduf-6
nuo-5
nuo-6
phb-2
rpl-3
RNAi treatment from L4
b
Pearson's r=0.927
ΔΔct rla-0
ΔΔct mdh−2
Pearson's r=-0.925
ΔΔc tag-120
ΔΔct mdh−2
Pearson's r=0.948
ΔΔct rpl-3
ΔΔct mdh−2
c
Proportion of correct predictions
Absolute cutoff Pearson's r = 0.75
mdh−2
ets−4
rpl−3
nduf−6
mean = 0.1021
acdh−1
nuo−6
haf−9
bcat−1
nuo−5
phb−2
Number of predictions
d
middle PFE/AUFE GRN
max AUFE GRN
max PFE GRN
skn1Steinbaugh2015
set15Inhouse
mxl2Nakamura2017
mml1Nakamura2017
daf16Wang2018
hlh30Lin2018
lin28Wang2017
set26Wang2018
hlh30Nakamura2017
0
1
2
3
Z-score Mutual Information

Figure S2. ***In vivo*** **measurement of the local accuracy of networks and** ***in silico*** **validation of network topology, Related to Figure 2.**

a. Percentages of knockdowns in each of six biological replicates obtained using RNAi against a set of regulators. *glp-1(e2144)ts* worms were fed with OP50 until L4 and were then transferred to RNAi bacteria until collection at day 2 adulthood (D2). Each dot represents delta-delta Ct values (ddCt) obtained by qRTPCR in each of the 6 biological replicates.

b. Example depicting the correlation between the levels of expression of a putative regulator versus the expression of a target gene. This variability of knockdown per replicate was exploited to measure the functional relationship between a regulator with its predicted targets. The plot shows relative expression level (ddCt values measured by qRTPCR) of *rla-0*, *rpl-3* and *tag-120* genes (Y-axis) at day 2 of *glp-1(e2144)ts* adulthood upon the RNAi-mediated knockdown of their predicted regulator *mdh-2* (X-axis). Each dot represents ddCt values in each of the 6 biological replicates. The Pearson's correlation (top of each plot) for each pair was calculated based on the ddCt values, representing an interaction between the predicted target and the knockdown *mdh-2* regulator.

c. Proportion of correct predictions as a function of the number of predictions for the 10 regulators. We considered a prediction to be correct if the absolute value of Pearson's correlation between the expression of its regulator and its target is superior or equal to 0.75. The mean proportion of correct predictions achieved by the 10 regulators is shown in blue. The fact that the proportion of validated predictions is higher for nodes that have a lower number of edges indicates that there is an unbiased error in the inferred network.

d. ***In silico*** **validation of network topology according to mutual information.** For the three consensus networks we consider the partitions of genes into structural modules according to the different levels in the SBM hierarchy (**Methods, Table S8a-c**); and into five empirical modules in terms of the different levels of gene expression fold change upon a gene knockdown (**Figure 2d**). For each pair of SBM structural modules and RNA-seq empirical modules, we compute the mutual information. The mutual information is an information-theory metric that assesses the similarities between two distributions of a set of elements into groups[65]. We then estimate the Z-score of the observed mutual information from the distribution of mutual information obtained by reshuffling module memberships. Each plot shows the Z-scores of a consensus network per RNA-seq datasets specific to *glp-1(e2144)ts* (row) and SBM hierarchical level (colours according to legend). The shaded area represents the non-significant Z-scores after Bonferroni multiple comparison correction. Note that every network has SBM partitions significantly similar to empirical modules for hierarchical levels >1. Since level 1 is the most coarse-grained, functionally significant hierarchical level, we use this level in further analyses in the manuscript.

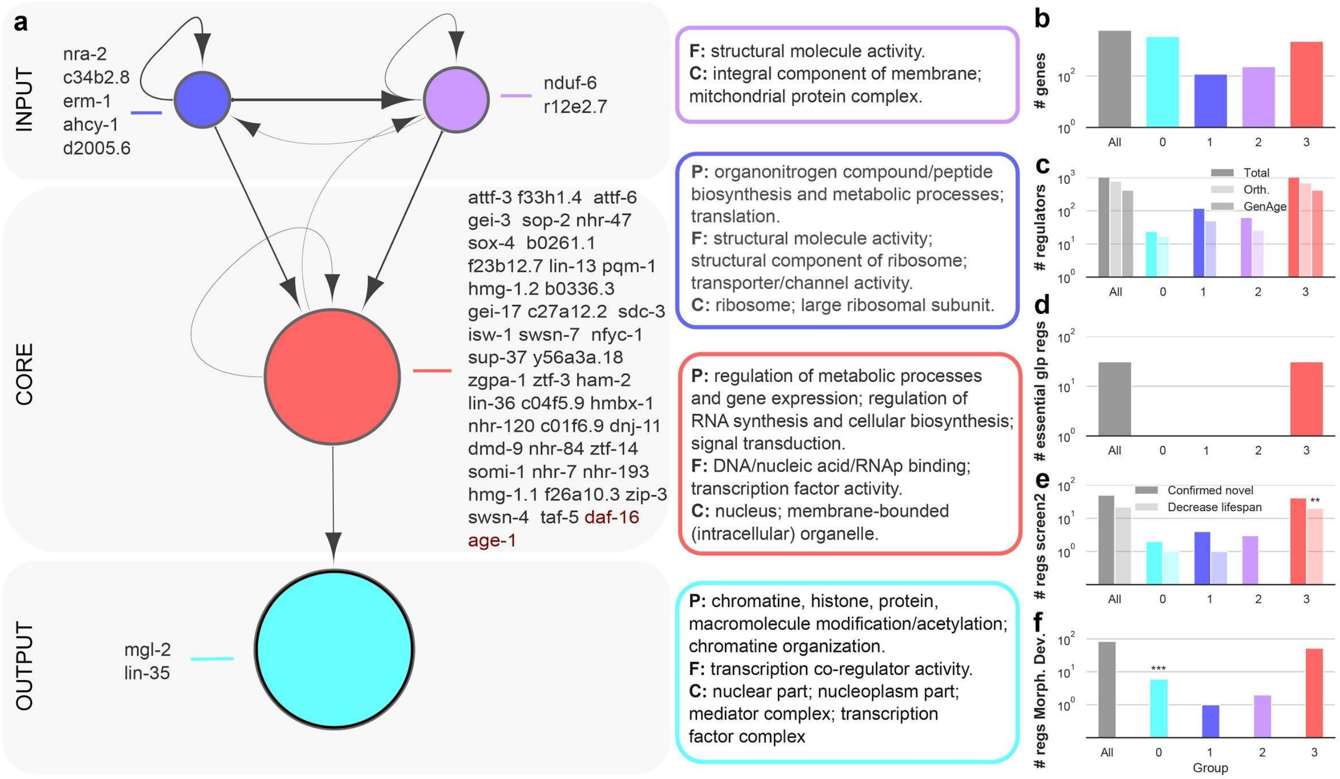

Figure S3. The global organization of the gene-regulation network reveals an input-core-output structure with ageing modulators at its core, Related to Figure 3 but for the max PFE network.

a. Global organisation of the network and GO enrichment for each module. We obtained structural modules using a Bayesian model selection approach with hierarchical stochastic block models (**Methods**). The area of the nodes is proportional to the number of genes it comprises (shown in **b**); and edge weights are proportional to their weight (we only represent edges with weight > 260-- **Methods**). The network has three main layers: input, core and output according to the inter and intra-layer regulatory interactions (see text). Newly-discovered ageing regulators are listed according to their network nodes in each layer, including *daf-1* and a*ge-1* (red) for reference. Boxes show the GO enrichment terms of the regulators in each module following the colour code of the network nodes (P: Process; F:Function; C:Component).

b. Number of genes in each module. Bars follow the same colour code of nodes in **a.** Grey bars show network totals.

c. Number of regulators, number of regulators with a human orthologue and number of regulators in the GenAge database.

d. Number of known essential genes in *glp-1 C. elegans* (Table S11) that appear as regulators in the network.

e. Number of tested and confirmed ageing genes in the second screen. *,** show enrichment with respect to the random expectation of hits given the number of non GenAge regulators in each module (* p-value <0.1, ** p-value <0.05, *** p-value <0.001).

f. Number of regulating genes that cause defects in body morphology (according to Kamath and Ahringer, 2003).

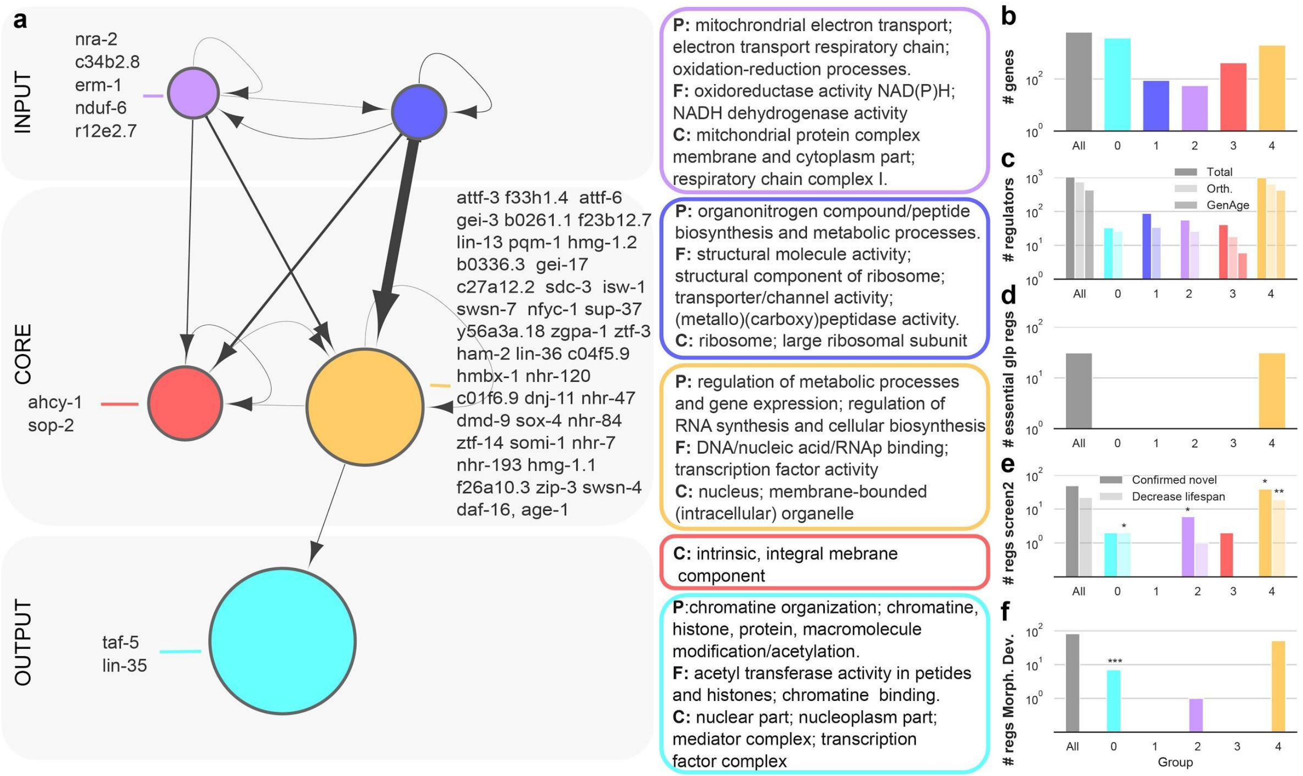

Figure S4. The global organization of the gene-regulation network reveals an input-core-output structure with ageing modulators at its core, Related to Figure 3 but for the middle PFE/AUFE network.

a. Global organisation of the network and GO enrichment for each module. We obtained structural modules using a Bayesian model selection approach with hierarchical stochastic block models (**Methods**). The area of the nodes is proportional to the number of genes it comprises (shown in **b**); and edge weights are proportional to their weight (we only represent edges with weight > 260-- **Methods**). The network has three main layers: input, core and output according to the inter and intra-layer regulatory interactions (see text). Newly-discovered ageing regulators are listed according to their network nodes in each layer, including *daf-1* and a*ge-1* (red) for reference. Boxes show the GO enrichment terms of the regulators in each module following the colour code of the network nodes (P: Process; F:Function; C:Component).

b. Number of genes in each module. Bars follow the same colour code of nodes in **a.** Grey bars show network totals.

c. Number of regulators, number of regulators with a human orthologue and number of regulators in the GenAge database.

d. Number of known essential genes in *glp-1 C. elegans* (Table S11) that appear as regulators in the network.

e. Number of tested and confirmed ageing genes in the second screen. *,** show enrichment with respect to the random expectation of hits given the number of non GenAge regulators in each module (* p-value <0.1, ** p-value <0.05, *** p-value <0.001).

f. Number of regulating genes that cause defects in body morphology (according to Kamath and Ahringer, 2003).

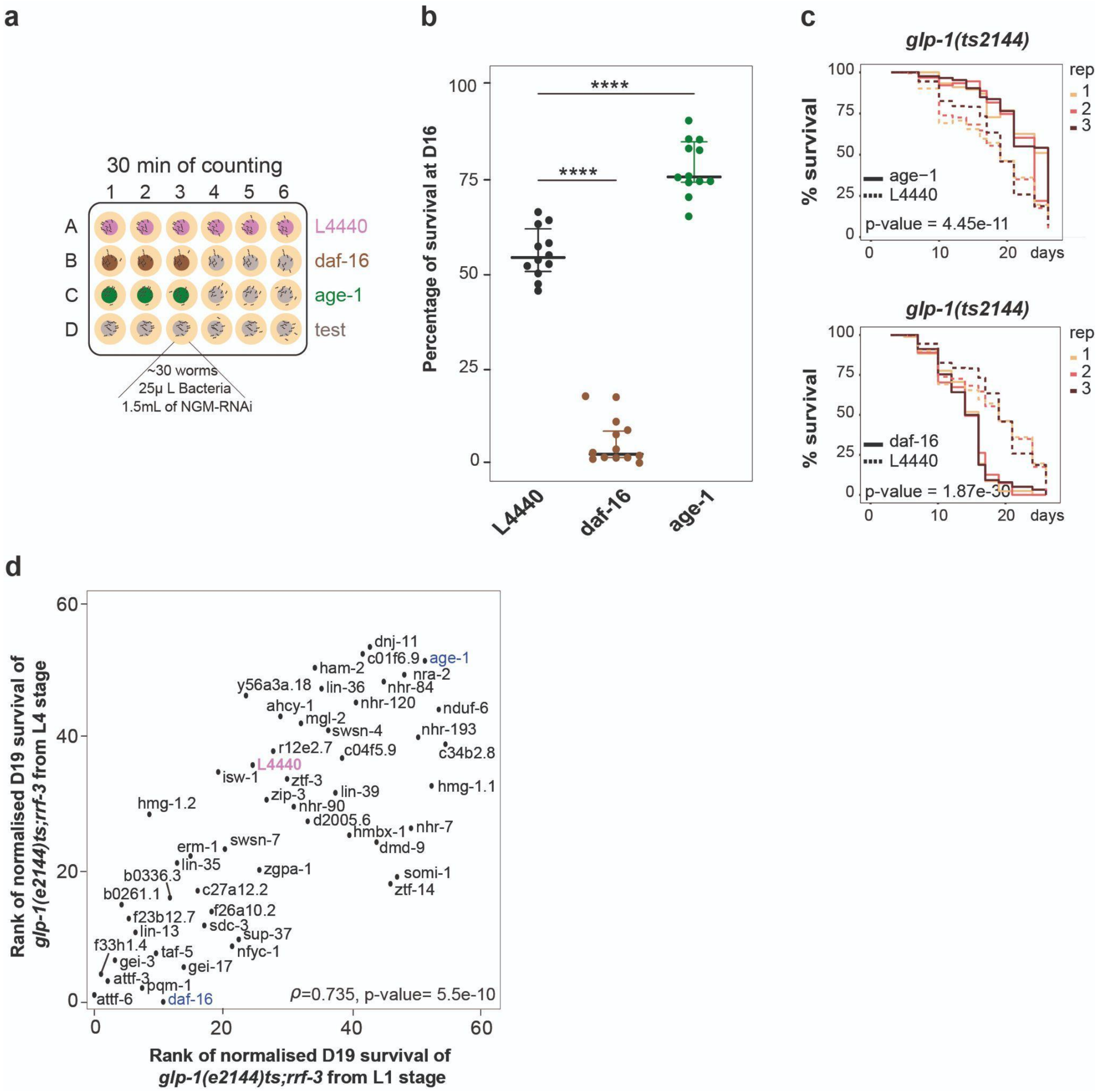

Figure S5. RNAi screening to uncover novel ageing modulators, Related to Figure 4.

a. Schematic representation of a screen plate considered a biological replicate. The amount of NGM, bacterial culture and worms are shown.

b. The mean survival of positive controls confirmed the expected effect on lifespan. Percentage of survival of *glp-1(e2144)ts;rrf-3* animals at day 16 of adulthood (D16) fed with bacteria expressing a negative control: an empty vector (L4440, black), and two positive controls: *daf-16* dsRNA (red) or *age-1* dsRNA (green), which decrease and increase *glp-1(e2144)ts* lifespans respectively. Each dot represents a biological replicate. For each biological replicate at least 6 wells

with 30 worms were analysed. p-values are based on 12 biological replicates using a one-way ANOVA test with Dunnett's correction.

c. Detailed lifespan assays showed expected effect on lifespan for positive controls. The percentage of survival of *glp-1(e2144);rrf-3* as a function of time is represented for the controls. The results of three biological replicates are shown. Top panel, worms fed with bacteria expressing *age-1* dsRNAs (full lines) or the empty vector (dashed lines). Lower panel, worms fed with bacteria expressing *daf-16* dsRNAs (full line) or the empty vector (dashed line). The p-value for each biological replicate was obtained using a log-rank test and then the 3 p-values were combined using Fisher's method. As expected *age-1* extends *glp-1(e2144)ts* lifespan whilst *daf-16* shortens it.

d. Correlation between the rank of normalised D19 survival of *glp-1(e2144)ts;rrf-3* worms treated with bacteria expressing 50 dsRNAs from L1 stage vs from L4 stage (**Table S14b**). We ranked genes according to the mean percentage of survival of long-lived *glp-1(e2144)ts* at D19 treated with dsRNAs from L1 stage, and the mean percentage of survival of the *glp-1(e2144)ts* at D19 treated with dsRNAs from L4 stage The RNAi conditions correspond to the novel ageing genes. We used Spearman's ρ to quantify the correlation between the ranked lists of genes.

a

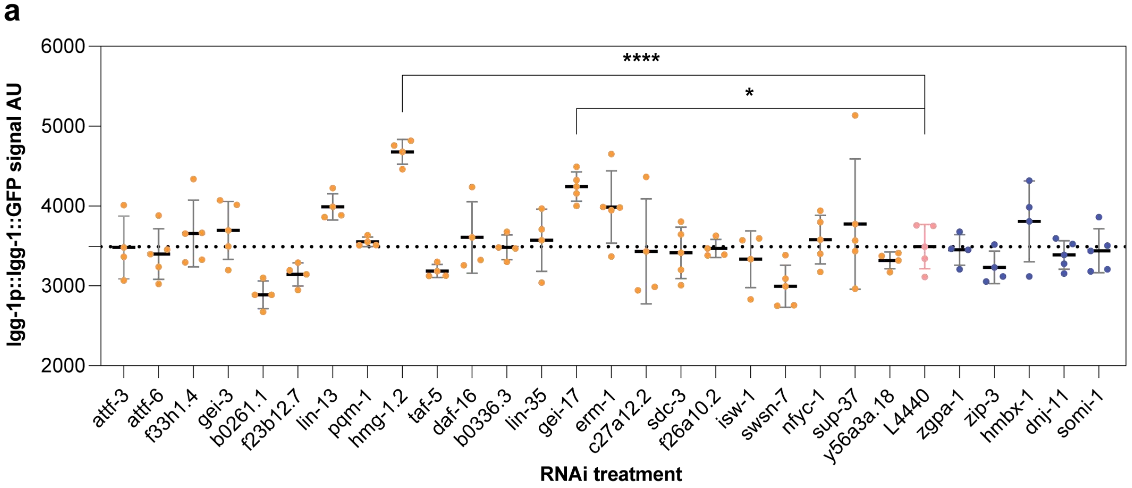

b

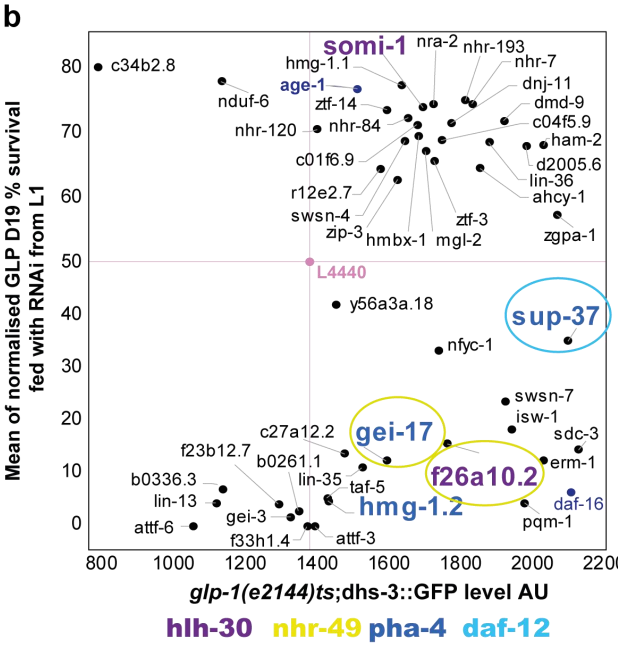

c

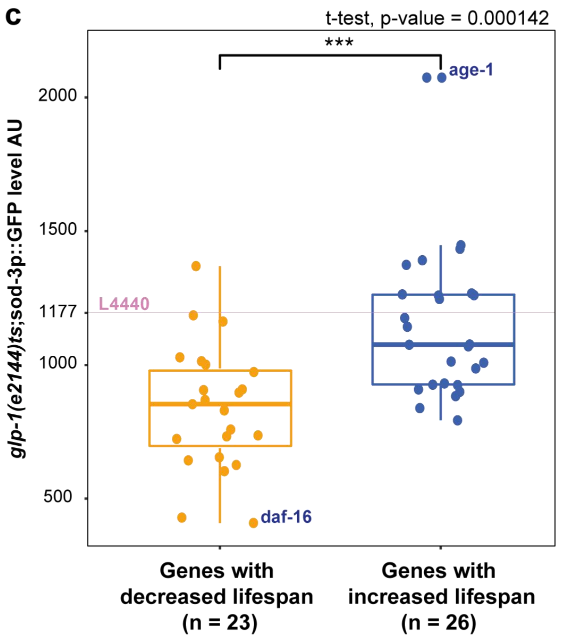

d

Regulator	Mean lifespan	Target	Ageing pathway	PCC	adjusted p-values
daf-16	-40.09	akt-1	ILS	0.818	<1e-3
daf-16	-40.09	daf-2	ILS	0.975	<1e-3
f26a10.2	-31.57	daf-2	ILS	-0.891	0.00699
f26a10.2	-31.57	hlh-30	other	-0.931	<1e-3
f26a10.2	-31.57	nhr-49	other	-0.835	0.0379
gei-17	-34.55	nhr-49	other	-0.889	0.0249
somi-1	26.79	hlh-30	other	0.840	0.0349
sup-37	-13.74	daf-18	ILS	-0.939	<1e-3
sup-37	-13.74	daf-12	other	-0.893	0.0239
sup-37	-13.74	pha-4	other	-0.941	<1e-3
zgpa-1	8.194	daf-2	ILS	-0.908	0.0139

e

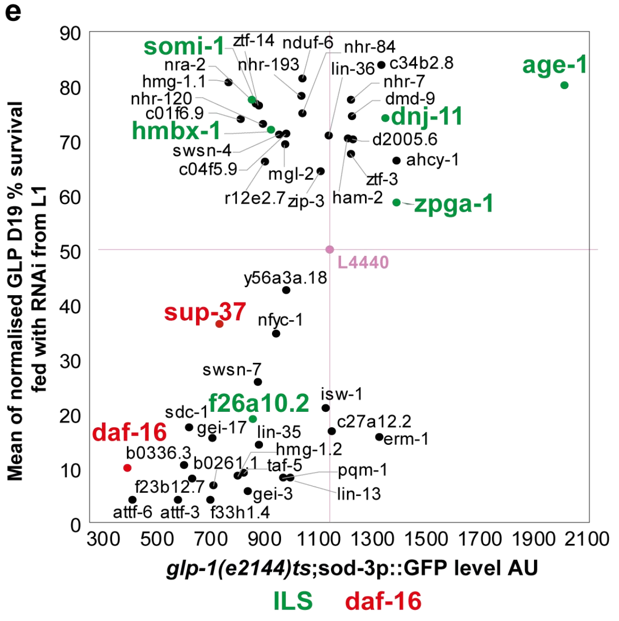

Figure S6. The novel ageing genes cause metabolic remodeling, Related to Figure 5.

a. Knockout of specific novel ageing genes alters the expression of a PHA-4/FOXA target. Knockouts of *hmg-1.2* and *gei-17* impact the expression levels of *lgg-1* in *glp-1(ts)* animals. Measures of the *in vivo* fluorescence of *lgg-1p::*LGG:1::GFP in day 4 adult *glp-1(e2144)ts* worms fed with dsRNA for the genes shown in the X-axis. The ageing genes that decrease *glp-1ts* lifespan are highlighted in orange and those that increase *glp-1(ts)* lifespan are highlighted in blue. We assayed the fluorescence level of GFP in approximately 30 worms in four biological replicates, each dot representing one independent replica of 30 worms (**Table S15a**). p-values were obtained using a mixed effect model.

b. Knockout of the novel ageing genes alters fat stores systematically. Comparison of the mean normalised percentage of survival of *glp-1(e2144)ts;rrf-3* at day 19 (D19) with respect to the i*n vivo* fluorescence level of the lipid droplet *in vivo* marker, *dhs-3p*::DHS-3::GFP reporter. Assays were performed in animals fed from L1 with RNAi bacteria (**Table S12, S15**). Pink dot shows the survival of worms fed with the empty vector (L4440), the positive controls are shown (dsRNA *age-1* and *daf-16* in blue). Black dots represent values of the novel ageing genes. Novel ageing genes with predicted functional interactions are highlighted. The predictions are based on *in vivo* screens (**Figure S6a** *hmg-1.2* and *gei-17*) and the PCC network (**Figure S6d**. *gei-17*, *f26a10.2*, *sup-37*, *somi-1*) which include interactions with *pha-4* (blue), *nhr-49* (yellow), *hlh-30* (purple), and *daf-12* (light blue).

c. Relationship between a DAF-16 activation and lifespan change. Comparison of the i*n vivo* fluorescence of the DAF-16 target *sod-3* across *glp-1(e2144)ts* worms fed with dsRNA that decrease lifespan (in orange) and those that increase lifespan (in blue). GFP fluorescence from a *sod-3p*::GFP transcriptional reporter at day 4 of adulthood. We assayed the fluorescence level of *sod-3p::GFP* in approximately 30 worms in four biological replicates (**Table S15**). The genes plotted led to a significantly changed mean normalised percentage of survival of *glp-1(e2144)ts;rrf-3* at day 19 (Bonferroni-corrected, Fisher's-combined p-values < 0.05) according to three biological replicates of 12-timepoint lifespan assays (**Table S12**).

d. PCC network as a tool for mechanistic predictions with regards to longevity pathway predictions. Table with values were obtained from the weighted gene-directed interaction network obtained by computing PCC from PCR gene expression data (https://s-andrews.github.io/wormgrn/qpcr/).

e. Summary of the mechanistic predictions of pathway membership. Genes are predicted to influence *glp-1(ts)* lifespan via DAF-16 and DAF-2/ILS. The plot shows the mean normalised percentage of survival of *glp-1(e2144)ts;rrf-3* at day 19 (D19) and the expression levels from an *in vivo sod-3* transcriptional reporter. It summarises functional interactions predicted by the qRTPCR network (*zpga-1, f26a10.2, somi-1, hmbx-1*; **Figure S6d**) and genetic epistasis experiments (**Figure 5c-d**, *sup-37*). Red dots highlight proven interactions with *daf-16* and green dots show predicted interactions with *daf-2*/ILS.

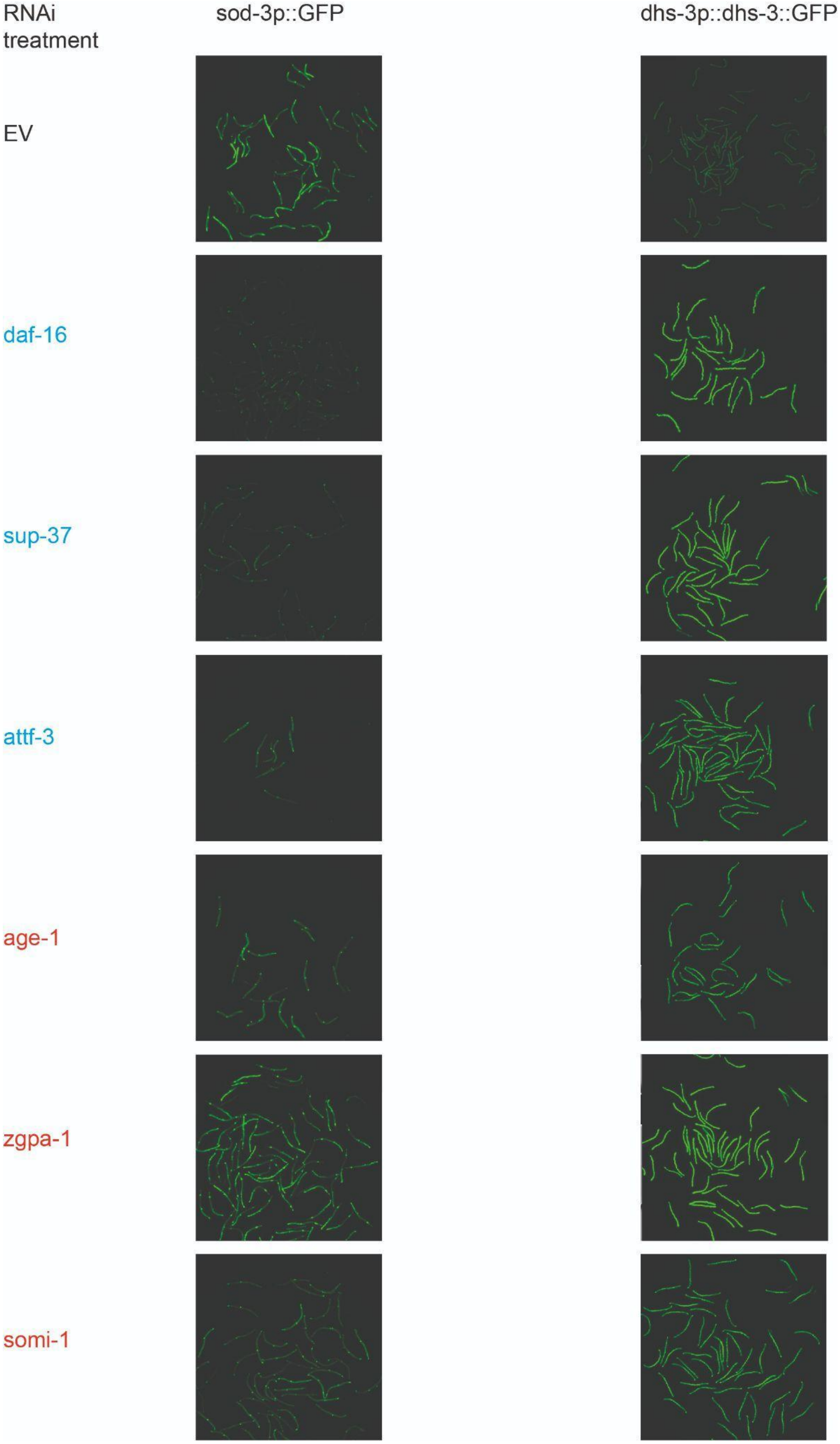
RNAi treatment
sod-3p::GFP
dhs-3p::dhs-3::GFP
EV
daf-16
sup-37
attf-3
age-1
zgpa-1
somi-1

Figure S7. The new ageing candidates are impacting the expression of sod-3 and dhs-3.
Fluorescent images of day-4 transgenic strain *glp-1(e2144)ts*;*sod-3p*::GFP (left) and *glp-1(e2144)ts*; *dhs-3p::dhs-3::GFP* (right) animals after treatment with empty vector or RNAi from L1 (top panel) and the indicated RNAi treatments. Genes in red correspond to RNAi conditions that reduce the *glp-ts* lifespan whereas genes in blue correspond to RNAi conditions that extend *glp-1ts* lifespan. Representative images related to *glp-1ts, lgg-1:lgg-1:gfp*; *fem-1ts, sod3gfp*, *fem-1ts, dhs-3p::dhs-3::GFP* can be found in Zenodo repository: https://zenodo.org/record/5499464#.YUDq8lMzb_o.
.

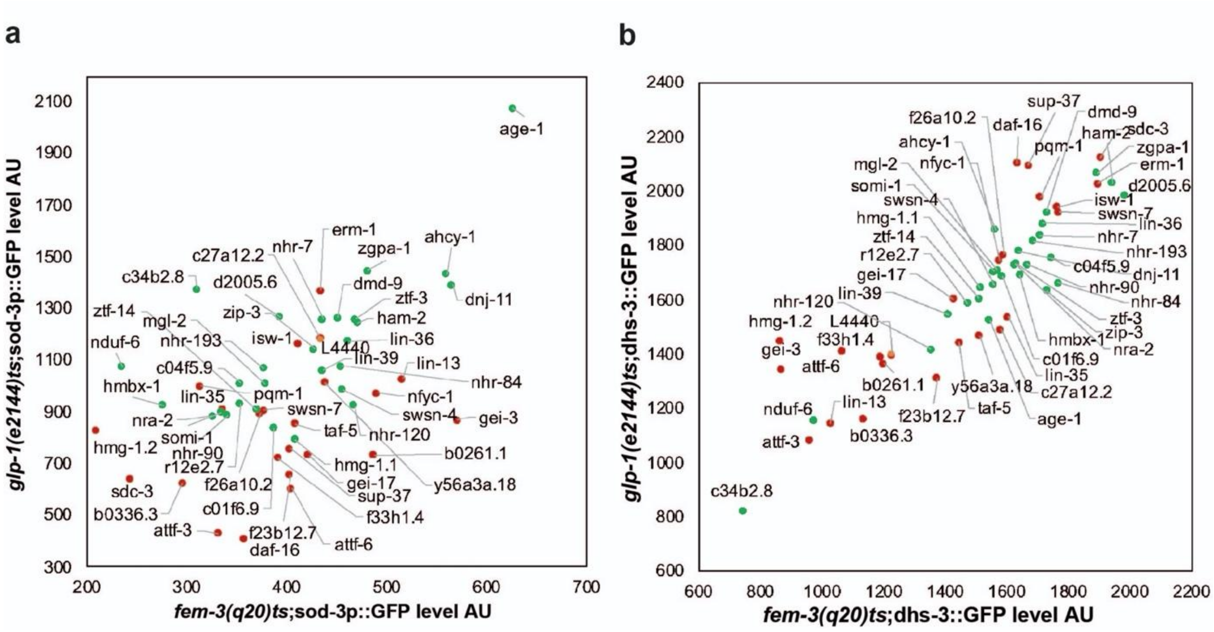

Figure S8. Relationship of fat storage and oxidative stress in *fem-3* animals.

a. Knockout of the novel genes alters fat stores in in the same manner in *glp-1(ts)* and *fem-3(ts)* Comparison of the *in vivo* fluorescence measures of *dhs-3p*::dhs-3::GFP in *glp-1(e2144)ts* versus *dhs-3p*::dhs-3::GFP in *fem-3(q20)ts* of animals at day 4 of adulthood. Green dots represent treatments that extend lifespan and red dots correspond to treatments that shorten lifespan. L4440 is the control/ empty vector. *fem-3ts* animals are sterile but have a wild type lifespan (**Table S15**).

b. Knockout of the novel genes impacts a DAF-16 target gene in the same manner in *glp-1(ts)* and fem-3(ts) Comparison of the *in vivo* fluorescence measures of *sod-3p*::GFP in *glp-1(e2144)ts* versus *sod-3p*::GFP in *fem-3(q20)ts* of animals at day 4 of adulthood. Green dots represent treatments that extend lifespan and red dots correspond to treatments that shorten lifespan.. L4440 is the control/ empty vector (**Table S15**).

Table S4. List of 50 inferred networksand their scores

Strain	inputData	Tools	Prior Code	Prior	Regulator Set	Measurable Reg	Fold Enrichment	Network Size	AUFE
glp	Full length	Inferelator	A	ChIP-Seq + ATAC-Seq	all TFs	90	0.70785244	35086	2.140 68959
glp	To 93 hr	Inferelator	A	ChIP-Seq + ATAC-Seq	all TFs	90	0.489583	36332	2.254 16918
glp	Full length	Inferelator	A	ChIP-Seq + ATAC-Seq	ageing genes	64	0.73302929	32936	3.082 04796
glp	To 93 hr	Inferelator	A	ChIP-Seq + ATAC-Seq	ageing genes	64	0.52098121	32893	3.022 51295
glp	Full length	Inferelator	B	ChIP-Seq + eY1H +ATAC-Seq	all TFs	87	0.64222305	32607	2.293 68756
glp	To 93 hr	Inferelator	B	ChIP-Seq + eY1H +ATAC-Seq	all TFs	89	0.54482747	34583	2.398 16284
glp	Full length	Inferelator	B	ChIP-Seq + eY1H +ATAC-Seq	ageing genes	62	0.66026993	33001	3.082 52805
glp	To 93 hr	Inferelator	B	ChIP-Seq + eY1H +ATAC-Seq	ageing genes	62	0.5071517	32413	3.359 0104
glp	Full length	Inferelator	C	ChIP-Seq + CisDB +ATAC-Seq	all TFs	89	0.68460105	33497	2.597 29828
glp	To 93 hr	Inferelator	C	ChIP-Seq + CisDB +ATAC-Seq	all TFs	91	0.55798263	35046	2.268 04163
glp	Full length	Inferelator	C	ChIP-Seq + CisDB +ATAC-Seq	ageing genes	64	0.73145126	32482	3.140 47268
glp	To 93 hr	Inferelator	C	ChIP-Seq + CisDB +ATAC-Seq	ageing genes	64	0.5396698	32671	2.905 78325
glp	Full length	Inferelator	D	ChIP-Seq + eY1H + CisDB +ATAC-Seq	all TFs	90	0.6325589	31699	2.250 15228
glp	To 93 hr	Inferelator	D	ChIP-Seq + eY1H + CisDB +ATAC-Seq	all TFs	91	0.52049528	33839	2.197 55652
glp	Full length	Inferelator	D	ChIP-Seq + eY1H + CisDB +ATAC-Seq	ageing genes	64	0.67669071	31507	3.109 80849
glp	To 93 hr	Inferelator	D	ChIP-Seq + eY1H + CisDB +ATAC-Seq	ageing genes	64	0.47584173	32161	3.061 34518
glp	Full length	Inferelator	No priors	No priors	all TFs	92	1.23380021	42518	2.367 59059
glp	To 93 hr	Inferelator	No priors	No priors	all TFs	92	1.03748549	43583	2.666 95882
glp	Full length	Inferelator	No priors	No priors	ageing genes	64	1.15486972	38626	3.329 26927
glp	To 93 hr	Inferelator	No priors	No priors	ageing genes	64	0.90719451	38108	3.231 37233
glp	Full length	Merlin	A	ChIP-Seq +	all TFs	81	1.47768283	8282	2.157

				ATAC-Seq					58967
glp	To 93 hr	Merlin	A	ChIP-Seq + ATAC-Seq	all TFs	84	1.42339759	6303	1.734 18025
glp	Full length	Merlin	A	ChIP-Seq + ATAC-Seq	ageing genes	60	1.29162263	8545	3.175 75132
glp	To 93 hr	Merlin	A	ChIP-Seq + ATAC-Seq	ageing genes	59	1.59449886	6186	3.304 29223
glp	Full length	Merlin	B	ChIP-Seq + eY1H +ATAC-Seq	all TFs	82	1.40140601	8269	2.249 96704
glp	To 93 hr	Merlin	B	ChIP-Seq + eY1H +ATAC-Seq	all TFs	83	1.53038667	6273	1.752 63342
glp	Full length	Merlin	B	ChIP-Seq + eY1H +ATAC-Seq	ageing genes	60	1.1995263	8454	3.019 44612
glp	To 93 hr	Merlin	B	ChIP-Seq + eY1H +ATAC-Seq	ageing genes	60	1.65803791	6192	3.255 05497
glp	Full length	Merlin	C	ChIP-Seq + CisDB +ATAC-Seq	all TFs	82	1.32404513	8360	1.827 34248
glp	To 93 hr	Merlin	C	ChIP-Seq + CisDB +ATAC-Seq	all TFs	84	1.62635756	6322	1.569 32941
glp	Full length	Merlin	C	ChIP-Seq + CisDB +ATAC-Seq	ageing genes	60	1.25791288	8357	3.153 25173
glp	To 93 hr	Merlin	C	ChIP-Seq + CisDB +ATAC-Seq	ageing genes	60	1.53405701	6326	2.991 88819
glp	Full length	Merlin	D	ChIP-Seq + eY1H + CisDB +ATAC-Seq	all TFs	76	1.17552261	5323	1.266 92729
glp	To 93 hr	Merlin	D	ChIP-Seq + eY1H + CisDB +ATAC-Seq	all TFs	83	1.45350142	6261	1.678 30276
glp	Full length	Merlin	D	ChIP-Seq + eY1H + CisDB +ATAC-Seq	ageing genes	60	1.30275826	8434	3.150 25606
glp	To 93 hr	Merlin	D	ChIP-Seq + eY1H + CisDB +ATAC-Seq	ageing genes	61	1.59309417	6209	3.202 85007
glp	Full length	Merlin	No priors	No priors	all TFs	78	1.59791348	5162	1.353 91743
glp	To 93 hr	Merlin	No priors	No priors	all TFs	82	1.3983987	5416	1.410 36014
glp	Full length	Merlin	No priors	No priors	ageing genes	59	1.28888757	6197	2.736 56176
glp	To 93 hr	Merlin	No priors	No priors	ageing genes	60	1.53232711	6221	3.274 66448
glp	Full length	Time-lagged Lasso	A	ChIP-Seq + ATAC-Seq	ageing genes + TF	12	1.48124609	267077	4.323 33409
glp	Full length	Time-lagged Lasso	B	ChIP-Seq + eY1H +ATAC-Seq	ageing genes + TF	12	1.47524433	267791	4.319 37687
glp	Full length	Time-lagged Lasso	C	ChIP-Seq + CisDB +ATAC-Seq	ageing genes + TF	12	1.4768733	267489	4.318 52634
glp	Full length	Time-lagged	D	ChIP-Seq +	ageing	12	1.48666072	268400	4.313

		Lasso		eY1H + CisDB +ATAC-Seq	genes + TF				73116
glp	Full length	Time-lagged Lasso	No priors	No priors	ageing genes + TF	52	0.7886536	290360	3.454 24236
glp	To 93 hr	Time-lagged Lasso	A	ChIP-Seq + ATAC-Seq	ageing genes + TF	1	0	28754	0
glp	To 93 hr	Time-lagged Lasso	B	ChIP-Seq + eY1H +ATAC-Seq	ageing genes + TF	1	0	28888	0
glp	To 93 hr	Time-lagged Lasso	C	ChIP-Seq + CisDB +ATAC-Seq	ageing genes + TF	1	6.95640327	47237	8.847 42287
glp	To 93 hr	Time-lagged Lasso	D	ChIP-Seq + eY1H + CisDB +ATAC-Seq	ageing genes + TF	1	2.91771429	59400	7.978 29238
glp	To 93 hr	Time-lagged Lasso	No priors	No priors	ageing genes + TF	44	1.18229366	253013	3.106 85796

Table S5. List of the 50-networkmembership by the nine block consensus networks

Block number	Network name by their input variables	Block summary
1	priorSetC-fullInput-ageingGenes-Inferelator	All Inferelator networks except prior set A
	priorSetB-shortInput-ageingGenes-MERLIN-P	All MERLIN-P ageing genes networks using short time series
	priorSetC-fullInput-TFs-Inferelator	
	priorSetC-shortInput-ageingGenes-Inferelator	
	priorSetC-shortInput-TFs-Inferelator	
	noPriors-fullInput-ageingGenes-MERLIN-P	
	noPriors-fullInput-TFs-Inferelator	
	noPriors-fullInput-ageingGenes-Inferelator	
	noPriors-shortInput-ageingGenes-Inferelator	
	noPriors-shortInput-TFs-Inferelator	
	priorSetD-shortInput-ageingGenes-MERLIN-P	
	priorSetD-fullInput-ageingGenes-Inferelator	
	priorSetC-shortInput-ageingGenes-MERLIN-P	
	priorSetD-fullInput-TFs-Inferelator	
	priorSetD-shortInput-ageingGenes-Inferelator	
	priorSetD-shortInput-TFs-Inferelator	
	priorSetB-fullInput-ageingGenes-Inferelator	
	priorSetA-shortInput-ageingGenes-MERLIN-P	
	priorSetB-fullInput-TFs-Inferelator	
	priorSetB-shortInput-ageingGenes-Inferelator	
	priorSetB-shortInput-TFs-Inferelator	
2	priorSetB-fullInput-TOL	All TOL with priors using full input data
	priorSetA-fullInput-TOL	
	priorSetC-fullInput-TOL	
	priorSetD-fullInput-TOL	
3	priorSetB-shortInput-TOL	TOL using prior set A and B with short data input
	priorSetA-shortInput-TOL	
4	priorSetD-shortInput-TOL	TOL using prior set C and D with short data input
	priorSetC-shortInput-TOL	
5	priorSetA-fullInput-TFs-Inferelator	All Inferelator using prior set A
	priorSetA-fullInput-ageingGenes-Inferelator	
	priorSetA-shortInput-ageingGenes-Inferelator	
	priorSetA-shortInput-TFs-Inferelator	
6	noPriors-shortInput-TOL	TOL using no priors
	noPriors-fullInput-TOL TOL using no priors	
	priorSetD-shortInput-TFs-MERLIN-P	
	priorSetB-shortInput-TFs-MERLIN-P	

7	priorSetC-shortInput-TFs-MERLIN-P	All MERLIN-P TF networks using short data input
	priorSetA-shortInput-TFs-MERLIN-P	
	noPriors-shortInput-TFs-MERLIN-P	
8	priorSetC-fullInput-ageingGenes-MERLIN-P	All MERLIN-P ageing gene networks using full data input
	priorSetA-fullInput-ageingGenes-MERLIN-P	
	priorSetD-fullInput-ageingGenes-MERLIN-P	
	priorSetB-fullInput-ageingGenes-MERLIN-P	
	noPriors-shortInput-ageingGenes-MERLIN-P	
9	priorSetD-fullInput-TFs-MERLIN-P	All MERLIN-P TF networks using full data input
	noPriors-fullInput-TFs-MERLIN-P	
	priorSetC-fullInput-TFs-MERLIN-P	
	priorSetA-fullInput-TFs-MERLIN-P	
	priorSetB-fullInput-TFs-MERLIN-P	

Table S9a. curated *glp-1(e2141ts)*-specific RNA-seq data used for this study

Knockdown gene	Age	Treatment	Quantitation detail	Data accession number	Reference
skn-1	day-1 adult	RNAi	Replicates did not separate well according to tSNE. Replicate 3 of the *glp-1* libraries and replicate 1 of *glp-1* + *skn-1* RNAi data were discarded. After removing the outliers, the dataset produced a consistent cumulative distribution plot. Proceeded with the standardSeqmonk RNA-seq quantitation pipeline, non-strand specific.	GSE63075	Steinbaugh et.al, 2015
mml-1*, *mxl-2*, *hlh-30	day-1 adult	RNAi	Good consistent quality. Quantitated by opposing strand-specific.	E-MTAB-3686	Nakamura et.al, 2016
tcer-1*, *daf-16	day-2 adult	RNAi	No RNA-seq data provided. Used the providedlist of genes in Supplementary table 1	N/A	Amrit et. al,2016
lin-28	day-4 adult	RNAi	Good consistent quality. Quantitated by samestrand-specific.	GSE86077	Wang et. al,2017
daf-16, hlh-30	L4	RNAi	Used only *hlh-30* data. *daf-16* data replicates were not consistent. After the standard Seqmonk quantitation (non-strand specific, applied percentile normalisation as the sizes ofthe libraries are quite different.	SRP152334	Lin et. al, 2018
set-26, daf-16	L4	RNAi	Good consistent quality. Quantitated by samestrand-specific.	GSE108848	Wang et. al, 2018
daf-16	day-1 adult	RNAi	Good consistent quality. Quantitated by samestrand-specific.	GSE111338	Chen et. al, 2019
set-15	day-1 adult	RNAi	Good consistent quality. Quantitated by opposing strand-specific	Our in-house data	

Table S9b. Additional ChIP-seq/ChIP-ChIP data curated for the study

Transcription Factor	Technique	Age	Background	GEO accession number	Reference
DAF-12	Chip-Chip	Mixed L2/adult	Wild type	GSE28350	Hochbaum et. al, 2011
DAF-16	Chip-Seq	Mixed stage	Wild type	GSE63865	Kumar et.al, 2015
HSF-1	Chip-Seq	Young adult	Wild type	GSE81521	Li et. al, 2016

Table S11. *Glp-1* essential genes

Type of interaction of glp-1(ts)	gene	reference	Type of interaction of glp-1(ts)
genetic	suppressing	acdh-11	Shen et al., 2012
genetic	suppressing	acs-17	Ratnappan et al., 2014; Ghazi A, Henis-Korenblit S & Kenyon C, 2007
genetic	suppressing	acs-2	Ratnappan et al., 2014
genetic	suppressing	acs-22	Amrit et al., 2016
genetic	suppressing	acs-22	Ratnappan et al., 2014
genetic	suppressing	atg-18	Lapierre LR, Gelino S, Melendez A & Hansen M, 2011
genetic	suppressing	atg-18	Lapierre 2011
gentic	suppressing	bec-1	Lapierre LR, Gelino S, Melendez A & Hansen M, 2011
genetic	suppressing	bec-1	Lapierre 2011
genetic	suppressing	cpt-2	Shen et al., 2012, Ratnappan et al., 2014
genetic	enhancing	cul-5	Ghazi A, Henis-Korenblit S & Kenyon C, 2007
genetic	enhancing	cul-6	Ghazi A, Henis-Korenblit S & Kenyon C, 2007
genetic	suppressing	daf-12	Hsin et al., 1999, Berman et al., 2006
genetic	suppressing	daf-16	Hsin et al., 1999; Nakamura et al., 2016; Hsin et al., 1999
genetic	suppressing	daf-36	Gerisch et al., 2001
genetic	suppressing	daf-9	Gerisch et al., 2001; Godeaux, 2011
genetic	suppressing	ech-7	Ratnappan et al., 2014
genetic	suppressing	F59B2.8	wormbase
genetic	suppressing	fard-1	McCormick et al., 2012; Lapiere et al., 2011
genetic	suppressing	fasn-1	Amrit et al., 2016
genetic	suppressing	fat-6	Godeaux, 2011
genetic	suppressing	fat-7	Godeaux, 2011
genetic	suppressing	fbxa-121	Ghazi A, Henis-Korenblit S & Kenyon C, 2007
genetic	suppressing	ftt-2	McCormick et al., 2012; Lapiere et al., 2011
genetic	suppressing	hacd-1	Ratnappan et al., 2014; Mahanti et al., 2014
genetic	suppressing	hlh-30	Lapierre 2013
genetic	suppressing	hpk-1	Mack HID, Zhang P, Fonslow BR & Yates JR, 2017
genetic	suppressing	K04A8.5	Wang et al., 2008
genetic	suppressing	kri-1	Berman et al., 2006
genetic	suppressing	let-363	Lapiere et al., 2011
genetic	suppressing	lgg-1	Lapiere et al., 2011
genetic	suppressing	lin-23	Ghazi A, Henis-Korenblit S & Kenyon C, 2007
genetic	suppressing	lipl-4	Berman et al., 2006; Lapierre 2011
genetic	suppressing	lips-17	McCornick 2012; Lapiere et al., 2011
genetic	suppressing	mbk-1	Mack HID, Zhang P, Fonslow BR & Yates JR, 2017
genetic	suppressing	mdt-15	McCornick 2012
genetic	suppressing	mir-241	Shen Y, Wollam J, Magner D, Karalay O & Antebi A, 2012
genetic	suppressing	mir-71	Boulias et al., 2012
genetic	suppressing	mir-84	Shen Y, Wollam J, Magner D, Karalay O & Antebi A, 2012
genetic	suppressing	mlcd-1	Amrit et al., 2016; Ratnappan et al., 2014
genetic	suppressing	mml-1	Nakamura et al., 2016

genetic	suppressing	mxl-2	Nakamura et al., 2016; Shen et al., 2012
genetic	suppressing	nhr-49	Ratnappan et al., 2014, Wei et al., 2016
genetic	suppressing	nhr-80	Godeaux, 2011; Nakamura et al., 2016
genetic	suppressing	pas-5	Ghazi A, Henis-Korenblit S & Kenyon C, 2007
genetic	suppressing	pas-6	Ghazi A, Henis-Korenblit S & Kenyon C, 2007
genetic	suppressing	pbs-2	Ghazi A, Henis-Korenblit S & Kenyon C, 2007
genetic	suppressing	pbs-3	Ghazi A, Henis-Korenblit S & Kenyon C, 2007; Berman et al., 2006
genetic	suppressing	pbs-4	Ghazi A, Henis-Korenblit S & Kenyon C, 2007
genetic	suppressing	pbs-5	Ghazi A, Henis-Korenblit S & Kenyon C, 2007; Godeaux, 2011
genetic	suppressing	pbs-6	Ghazi A, Henis-Korenblit S & Kenyon C, 2007
genetic	suppressing	pbs-7	Ghazi A, Henis-Korenblit S & Kenyon C,
genetic	suppressing	pha-4	Lapierre LR, Gelino S, Melendez A & Hansen M, 2011, Lapiere et al., 2011
genetic	suppressing	pha-4	Lapiere et al., 2011
genetic	suppressing	phi-62	Mccornick 2012
genetic	suppressing	pod-2	Amrit et al., 2016
genetic	suppressing	rme-2	Steinbaugh et al., 2015; Lapiere et al., 2011
genetic	suppressing	rpn-1	Ghazi A, Henis-Korenblit S & Kenyon C, 2007; Mahanti et al., 2014
genetic	suppressing	rpn-10	Ghazi A, Henis-Korenblit S & Kenyon C, 2007
genetic	suppressing	rpn-11	Ghazi A, Henis-Korenblit S & Kenyon C, 2007
genetic	suppressing	rpn-12	Ghazi A, Henis-Korenblit S & Kenyon C, 2007
genetic	suppressing	rpn-2	Ghazi A, Henis-Korenblit S & Kenyon C, 2007
genetic	suppressing	rpn-3	Ghazi A, Henis-Korenblit S & Kenyon C, 2007
genetic	suppressing	rpn-6.1	Ghazi A, Henis-Korenblit S & Kenyon C, 2007; Lapiere et al., 2011
genetic	suppressing	rpn-7	Ghazi A, Henis-Korenblit S & Kenyon C, 2007
genetic	suppressing	rpn-8	Ghazi A, Henis-Korenblit S & Kenyon C, 2007
genetic	suppressing	rpn-9	Ghazi A, Henis-Korenblit S & Kenyon C, 2007
genetic	suppressing	rpt-1	Ghazi A, Henis-Korenblit S & Kenyon C, 2007
genetic	suppressing	rpt-2	Ghazi A, Henis-Korenblit S & Kenyon C, 2007; Wei et al., 2016
genetic	suppressing	rpt-3	Ghazi A, Henis-Korenblit S & Kenyon C, 2007; Nakamura et al., 2016
genetic	suppressing	rpt-4	Ghazi A, Henis-Korenblit S & Kenyon C, 2007
genetic	suppressing	rpt-5	Ghazi A, Henis-Korenblit S & Kenyon C, 2007
genetic	suppressing	rpt-6	Ghazi A, Henis-Korenblit S & Kenyon C, 2007
genetic	suppressing	skn-1	Ratnappan et al., 2014, Steinbaugh et al., 2015
genetic	suppressing	tcer-1	Berman et al., 2006, Ratnappan et al., 2016, Ghazi et al., 2009
genetic	suppressing	unc-51	Lapierre LR, Gelino S, Melendez A & Hansen M, 2011
genetic	suppressing	unc-51	Lapiere et al., 2011
genetic	enhancing	utx-1	Ni Z, Ebata A, Alipanahiramandi E & Lee SS, 2012
genetic	suppressing	vps-34	Lapierre LR, Gelino S, Melendez A & Hansen M, 2011
genetic	suppressing	vps-34	Lapiere et al., 2011

Table S13a. Orthologues New ageing genes

**Name**	**Molecular function**	**% lifespan change at D19**	**χ2-adjusted p-value**	**Human disease**	**Human orthologue**	**Mouse orthologue**	**Fly orthologue**
**attf-3**	C2H2-type domain- typical of HMG/ Chromatin remodelling	-45.96	1.65E-50		orthologous to high mobility group box 1	C2H2-type domain- typical of HM/ Chromatin remodelling	
**f33h1.4**		-45.96	9.11E-48		Uncharacterised		
**attf-6**	AT-hook typical of HMG/ Chromatin remodelling	-45.96	8.59E-44		orthologous to high mobility group box 1	C2H2-type domain- typical of HM/ Chromatin remodelling	Flybase gene name is dpy-PQ
**gei-3**	Transcriptional repressor	-44.38	2.38E-32	Human ortholog(s) of this gene are implicated in autosomal dominant mental retardation 45	Is an ortholog of human CIC (capicua transcriptional repressor).	Protein capicua homolog	Flybase gene name is cic-PG
**b0261.1**	TF	-43.33	5.64E-25	Human ortholog(s) of this gene is a potential model for autosomal	Is an ortholog of human BDP1 (B double prime 1,	Transcription factor TFIIIB component B"	Flybase gene name is Bdp1-PA

				recessive nonsyndromic deafness 112.	subunit of RNA polymerase III transcription initiation factor IIIB)	homolog	
**f23b12.7**	DNA binding	-42.07	2.59E-21	Human ortholog(s) of this gene may be correlated with occurrence of acute myeloid leukemia (AML).	is an ortholog of human CEBPZ (CCAAT enhancer binding protein zeta)		
**lin-13**	Zinc finger protein	-41.94	1.10E-29	Human ortholog(s) of this gene are implicated in nephronophthisis 14.	Is an ortholog of several human genes including ZNF423 (zinc finger protein 423); ZNF462 (zinc finger protein 462); and ZNF786 (zinc finger protein 786).	Myoneurin	Flybase gene name is CG1233-PB
**pqm-1**	TF	-41.9	1.14E-15		cDNA FLJ60868, highly similar to Sal-like protein 2		Flybase gene name is salm-PA
**hmg-1.2**	HMG	-41.53	1.10E-19	Human ortholog(s) of this gene are implicated in syndromic microphthalmia 13.	Is an ortholog of human HMGB1 (high mobility group box 1) and HMGB3 (high mobility group box 3).	C2H2-type domain- typical of HM/ Chromatin remodelling	Flybase gene name is Dsp1-PF
**taf-5**	TATA box TF	-41.06	1.04E-28		is an ortholog of human TAF5 (TATA-box binding protein associated factor 5).	Transcription initiation factor TFIID subunit 5	Flybase gene name is Taf5-PA
**b0336.3**	RNA binding	-39.53	3.92E-21		Is an ortholog of human RBM26 (RNA binding motif protein 26) and RBM27 (RNA binding motif protein 27).	RNA-binding protein 26	Flybase gene name is swm-PD
**lin-35**	Transcriptional corepressor	-35.8	3.89E-19	Human ortholog(s) of this gene are implicated in several diseases, including lung small cell carcinoma; osteosarcoma; and retinoblastoma.	Is an ortholog of human RBL1 (RB transcriptional corepressor like 1) and RBL2 (RB transcriptional corepressor like 2)	Retinoblastoma-like protein 2	Flybase gene name is Rbf-PA
**gei-17**	TF	-34.55	7.61E-16		is an ortholog of several human genes including PIAS2 (protein inhibitor of activated STAT 2); PIAS3 (protein inhibitor of activated STAT 3); and PIAS4 (protein inhibitor of activated STAT 4).	E3 SUMO-protein ligase PIAS1	Flybase gene name is Su(var)2-10-PI
**erm-1**	Ezrin-radixin-moesin protein	-34.52	1.01E-13	Human ortholog(s) of this gene is a potential model for autosomal recessive nonsyndromic deafness 24.	is an ortholog of human EZR (ezrin); MSN (moesin); and RDX (radixin).		
**c27a12.2**	Zinc finger protein	-33.3	3.46E-12	Human ortholog(s) of this gene are implicated in nephronophthisis 14.	Is an ortholog of human ZNF791 (zinc finger protein 791).		Flybase gene name is CG15269-PA
**sdc-3**	Carboxypeptidase	-32.63	2.98E-07	Human ortholog(s) of this gene are implicated in familial febrile seizures 11 and familial temporal lobe epilepsy 5	Is an ortholog of several human genes including CPA3 (carboxypeptidase A3); CPA4 (carboxypeptidase A4); and CPB2 (carboxypeptidase B2). Exhibits sequence-specific DNA binding activity.	Cpa familly	yes

**f26a10.2**	TF	-31.57	4.51E-14	Human ortholog(s) of this gene are implicated in autosomal dominant non-syndromic intellectual disability 22.	Is an ortholog of several human genes including AC008481.3; ZBTB32 (zinc finger and BTB domain containing 32); and ZFP91-CNTF (ZFP91-CNTF readthrough (NMD candidate)).	Zinc finger protein 668	Flybase gene name is Kah-PA
**isw-1**	Predicted to be TF	-29.18	5.04E-13		Is an ortholog of human SMARCA1 (SWI/SNF related, matrix associated, actin dependent regulator of chromatin, subfamily a, member 1)		Flybase gene name is Iswi-PC
**swsn-7**	Chromatin remodelling	-24.35	3.46E-07	Human ortholog(s) of this gene are implicated in Coffin-Siris syndrome 6	s an ortholog of human ARID2 (AT-rich interaction domain 2).	AT-rich interactive domain-containing protein 2	Flybase gene name is Bap170-PA
**nfyc-1**	TF	-15.5	1.79E-04		Is an ortholog of human NFYC (nuclear transcription factor Y subunit gamma).		Flybase gene name is Nf-YC-PA
**sup-37**		-13.74	1.32E-14		Uncharacterised		
**y56a3a.18**	Zinc finger protein	-7.531	2.19E-06		Is an ortholog of human ZNF593 (zinc finger protein 593).	Zinc finger protein 593	Flybase gene name is CG3224-PA
**zgpa-1**	Zinc finger protein	8.194	1.32E-02		Is an ortholog of human ZGPAT (zinc finger CCCH-type and G-patch domain containing)		Flybase gene name is CG4709-PB
**zip-3**	TF	14.23	6.06E-04		Is an ortholog of human ATF5 (activating transcription factor 5).		
**r12e2.7**		16.05	4.49E-02		Uncharacterised		
**ahcy-1**	Adenosyl-hydrolase	16.24	2.69E-02	Human ortholog(s) of this gene are implicated in hypermethioninemia with deficiency of S-adenosylhomocysteine hydrolase.	Is an ortholog of human AHCY		Flybase gene name is Ahcy-PC
**ztf-3**	Zinc finger protein	17.51	3.93E-06		Isoform 1 of Hypermethylated in cancer 2 protein (HIC2)	Zinc finger protein 394	Flybase gene name is gl-PC
**mgl-2**	Glutamate receptor	19.19	1.48E-05	Human ortholog(s) of this gene are implicated in autosomal recessive spinocerebellar ataxia 13 and spinocerebellar ataxia 44.	is an ortholog of human GRM1 (glutamate metabotropic receptor 1) and GRM5 (glutamate metabotropic receptor 5).	Metabotropic glutamate receptor 1	Flybase gene name is mGluR-PB
**d2005.6**	Phosphorylation site; Membrane-associating domain; and Marvel domain.	20.18	1.83E-07				
**ham-2**	Zinc finger protein	20.24	4.83E-03		Isoform 1 of Zinc finger and BTB domain-containing protein 40	Histone-lysine N-methyltransferase PRDM16 {ECO:0000305}	Flybase gene name is Oaz-PC
**lin-36**	DNA and metal ion binding	20.78	2.43E-03				
**swsn-4**	SWI/SNF-chromatin	21.05	5.00E-04	Human ortholog(s) of this gene are implicated	Is an ortholog of human SMARCA2		Flybase gene name is brm-

	remodelling			in Coffin-Siris syndrome 4	(SWI/SNF related, matrix associated, actin dependent regulator of chromatin, subfamily a, member 2).		PE
**c04f5.9**	Zinc finger protein	21.13	2.28E-03		Zinc finger protein 319	Zinc finger protein 319	Flybase gene name is shn-PF
**sox-4**	Sox protein	21.54	4.00E-02	Human ortholog(s) of this gene is a potential model for 46,XX sex reversal 1.	is an ortholog of human SRY		
**hmbx-1**	Homeobox TF	21.86	6.44E-04		Is an ortholog of human HMBOX1 (homeobox containing 1)	Homeobox-containing protein 1	
**nhr-120**	TF	22.97	4.05E-04	Human ortholog(s) of this gene are implicated in maturity-onset diabetes of the young type 1 and type 2 diabetes mellitus	Is an ortholog of several human genes including HNF4A (hepatocyte nuclear factor 4 alpha); NR2C2 (nuclear receptor subfamily 2 group C member 2); and RXRB (retinoid X receptor beta).		Flybase gene name is Hr39-PD
**c01f6.9**	Zinc finger protein	23.83	2.79E-04		Is an ortholog of human ZNF706 (zinc finger protein 706)		Flybase gene name is CG15715-PB
**dnj-11**	Hsp40	24.06	2.15E-03		Is an ortholog of human DNAJC2 (DnaJ heat shock protein family (Hsp40) member C2).		Flybase gene name is CG10565-PB
**dmd-9**	DNA binding TF	24.49	6.50E-04		Isoform 1 of Doublesex- and mab-3-related transcription factor 2		
**nhr-47**	TF	24.58	3.83E-02		Isoform HNF4-Alpha-8 of Hepatocyte nuclear factor 4-alpha		
**nhr-84**	TF	24.99	3.90E-03		Nuclear receptor subfamily 2 group E member 1	0	Flybase gene name is tll-PA
**sop-2**	Polycomb protein	25.22	1.85E-02				
**ztf-14**	Zinc finger protein	26.38	1.99E-09	Human ortholog(s) of this gene are implicated in neonatal diabetes mellitus with congenital hypothyroidism	Is an ortholog of several human genes including GLIS1 (GLIS family zinc finger 1); GLIS3 (GLIS family zinc finger 3); and ZXDA (zinc finger X-linked duplicated A).	Flybase gene name is ci-PA	Transcriptional activator GLI3
**somi-1**	Suppressor of overexpressed micro-RNA	26.79	5.76E-08				
**nra-2**	Nicalin	27.38	2.88E-03		is an ortholog of human Ncln		Flybase gene name is CG4972-PA
**nhr-7**	TF	27.39	2.93E-03		NR4A2; ENSEMBL:ENSP00000394671: Uncharacterised protein		Flybase gene name is Hr38-PB
**nhr-193**	TF	28.08	2.82E-06		Isoform HNF4-Alpha-2 of Hepatocyte nuclear factor 4-alpha		Flybase gene name is Hnf4-PB
**hmg-1.1**	Chromatin remodelling	30.68	1.58E-07		Is an ortholog of human HMGB1 (high mobility group box 1)	C2H2-type domain- typical of HM/ Chromatin	

					and HMGB3 (high mobility group box 3).	remodelling	
**nduf-6**	Ubiquinone oxidoreductase	31.34	7.48E-08		Is an ortholog of human NDUFS6 (NADH:ubiquinone oxidoreductase subunit S6)	NADH dehydrogenase [ubiquinone] iron-sulfur protein 6, mitochondrial	Flybase gene name is ND-13A-PC
**c34b2.8**	Ubiquinone oxidoreductase	33.87	2.02E-15	Human ortholog(s) of this gene are implicated in thyroid gland Hurthle cell carcinoma.	Is an ortholog of human NDUFA13 (NADH:ubiquinone oxidoreductase subunit A13).		Flybase gene name is ND-B16.6-PC

S13b Controls used in the screen.

**Control**	**Function**	**Effect observed?**	**% lifespan at D19**	**χ2-adjusted p-value**
**sbp-1**	Sterol regulatory binding protein	shortens lifespan	-20,72	0.0001128
**pha-4**	FOXOA TF	extends lifespan	18,62	3.75E-08
**nhr-49**	nuclear hormone recptor	shortens lifespan	-42,77	6.39E-44
**kri-1**	Krev interaction trapped/cerebral cavernous malformation 1) homolog)	shortens lifespan	-19,26	4.19E-05
**age-1**	ILS receptor	extends lifespan	30,07	1.05E-13
**daf-16**	FOXO TF	shortens lifespan	-40,09	4.21E-35
**tkt-1**	Transketolase	extends lifespan	21.01	1.09E-07

Table S20. Lifespan epistasis Raw data

CB4037: *glp-1(e2141)*<br>BIOLOGICAL REPLICATE 1			
Number of death	**RNAi treatment**		
Day	EV	sdc-3	sup-37
0	0	0	0
5	1	0	0
6	4	4	5
7	8	9	5
8	9	32	36
9	9	59	33
10	9	71	38
11	33	24	34
12	41	21	26
13	43	7	17
14	20		17
15	19		19
16	12		17

17	7		11
18	9		2
19	2		
**Total Number**	226	227	260
**Number of censored**	0	0	0
**Median lifespan**	13	10	13
**p value (logrank Mantel cox test**		<0.0001	0.0007
Temperature in celsius	25	25	25

CB4037: *glp-1(e2141)*<br>BIOLOGICAL REPLICATE 2			
Number of death	**RNAi treatment**		
Day	EV	sdc-3	sup-37
0	0	0	0
5	0	0	0
6	2	1	1
7	3	6	3
8	4	15	12
9	3	34	15
10	5	32	20
11	11	11	14
12	18	12	12
13	16	3	8
14	11		7
15	6		9
16	5		8
17	3		5
18	4		1
19	1		
**Total Number**	92	114	115
**Number of censored**			
**Median lifespan**	12	10	11
**p value (logrank Mantel cox test**		<0.0001	0.0306
**p value (Gehan-Breslow-Wilcoxon test)**		<0.0001	0.0068
Temperature in celsius	25	25	25

CB4037: *glp-1(e2141)*<br>BIOLOGICAL REPLICATE 3			
Number of death	**RNAi treatment**		
Day	EV	sdc-3	sup-37
0	0	0	0
5	0	0	0
6	1	2	2
7	4	4	1
8	2	14	14
9	4	26	11
10	6	28	13
11	17	11	10
12	20	8	7
13	21	4	5
14	11	1	7
15	7		6
16	6		4
17	0		3
18	5		1
19	0		
20	1		
**Total Number**	105	98	84
**Number of censored**			
**Median lifespan**	13	10	14
**p value (logrank Mantel cox test**		<0.0001	0.1948
**p value (Gehan-Breslow-Wilcoxon test)**		<0.0001	0.4121
Temperature in celsius	25	25	25

CF1880: *daf-16(mu86) ; glp-1(e2141)*<br>BIOLOGICAL REPLICATE 1			
Number of death			
Treatment	L4440	sdc-3	sup-37
0	0	0	0
5	0	0	0
6	0	8	0
7	16	102	14
8	45	138	85
9	27	85	53
10	93	16	95
11	83		47
12	36		10

13	21		4
14	6		
**Total Number**	327	349	308
**Number of censored**	0	0	0
**Median lifespan**	11	8	10
**p value (logrank Mantel cox test**		<0.0001	0.1124 (ns)

CF1880: *daf-16(mu86) ; glp-1(e2141)* BIOLOGICAL REPLICATE 2			
Number of death	Treatment		
Day	EV	sdc-3	sup-37
0	0	0	0
5	0	0	0
6	1	2	0
7	6	34	4
8	18	45	22
9	10	30	26
10	39	5	40
11	34	1	14
12	12		4
13	7		2
14	1		
Total Number	128	117	112
Number of censored			
Median lifespan	11	9	10
p value (logrank Mantel cox test		<0.0001	0.6(ns)

CF1880: *daf-16(mu86) ; glp-1(e2141)* BIOLOGICAL REPLICATE 3			
Number of death	Treatment		
Day	EV	sdc-3	sup-37
0	0	0	0
5	0	0	0
6	0	4	0
7	6	39	5
8	15	64	26
9	10	33	20
10	27	5	35
11	29		16
12	14		3

13	8		1
14	2		
Total Number	111	145	106
Number of censored			
Median lifespan	11	8	10
p value (logrank Mantel cox test		<0.0001	0.86(ns)

Supplementary Table guide

Table name	File name	Repository DOI
**Table S1. Datasets used as prior information**	TableS1_datasets_for_prior.csv	10.5281/zenodo.4382337
**Table S2. Physical priors from mechanistic inference**	TableS2_physical_priors.xlsx	10.5281/zenodo.4382337
**Table S3. List of WT gold standards**	TableS3_WT_functional_priors.csv	10.5281/zenodo.4382337
**Table S4. List of 50 inferred networks and their scores**	TableS4_inferred_networks.csv	10.5281/zenodo.5499464
**Table S5. List of the 50 network membership by the nine block consensus networks**	TableS5_consensus_network_member.xlsx	10.5281/zenodo.5499464
**Table S6. Final three selected networks**	TableS6a_max_AUFE_network_BlockconsensusSigNetwork.txt, TableS6b_max_PFE_network_BlockconsensusSigNetwork.txt, TableS6c_middle_PFE_AUFE_network_BlockconsensusSigNetwork.txt	10.5281/zenodo.5499464
**Table S7. qRTPCR results and correlations used to calculate network accuracy**	TableS7_qRTPCR_ddCt_network_accuracy.xlsx	10.5281/zenodo.5499464
**Table S8. SBM division into hierarchy of modules of each network**	TableS8a_SBM_max_AUFE_network.csv, TableS8b_SBM_max_PFE_network.csv, TableS8c_SBM_middle_PFE_AUFE_network.csv	10.5281/zenodo.5499464
**Table S9. Datasets used for glp-gold standards**	TableS9_glp_gs_datasets.pdf	10.5281/zenodo.5499464
**Table S10. First screen on 1,120 network regulators on *glp-1(e2144)ts;rrf-3(pk1426)*- RNAi from L1 to DAY 16 adulthood**	TableS10_first_screen_lifespan_assay.xlsx	10.5281/zenodo.5499464
**Table S11. Genes that are essential for glp longevity according to Wormbase Version: WS278**	TableS11_glp_essential_genes.csv	10.5281/zenodo.5499464

**Table S12. Second screen for 93 top genes on *glp-1(e2144)ts;rrf-3(pk1426)* detailed lifespan assays, worms treated from L1**	TableS12_second_screen_lifespan_assay.xlsx	10.5281/zenodo.5499464
**Table S13. List of orthologous genes in fly, mouse, and human for the novel ageing genes**	TableS13_orthologues_new_ageing_genes.xlsx	10.5281/zenodo.5499464
**Table S14. Survival data of *glp-1(e2144)ts;rrf-3(pk1426)* RNAi and fem-3(q20)ts RNAi**	TableS14a_glp_l1_vs_fem_l1_lifespan_assay.xlsx<br>TableS14b_glp_l1_vs_glp_l4_lifespan_assay.xlsx	10.5281/zenodo.5499464
**Table S15. in vivo fluorescent reporter data of g*lp-1(e2144)ts;rrf-3(pk1426)* and fem-3(q20)ts**	TableS15a_glp1_in_vivo_fluorescence_data.xlsx<br>TableS15b_fem3_in_vivo_fluorescence_data.xlsx	10.5281/zenodo.5499464
**Table S16. qRTPCR results for the gene interaction network of new ageing genes and targets and the weighted, directed gene-interaction network**	TableS16_qRTPCR_gene_interaction_network.xlsx	10.5281/zenodo.5499464
**Table S17. List of genes used as an input regulator and its category (GenAge, TF or High variability) and orthologues**	TableS17_input_regulators_annotated.csv	10.5281/zenodo.5499464
**Table S18. Strain and primers used in the study**	TableS18_strain_primer_list.xlsx	10.5281/zenodo.5499464
**Table S19. List of non-variable genes used for data normalisation.**	TableS19_nonvariable_genes.csv	10.5281/zenodo.5499464
**Table S20. Epistasis lifespan data of *glp-1(e2144)ts***	TableS20_epistasis_lifespan_data.xlsx	10.5281/zenodo.5499464
